# *Drosophila melanogaster* Models for Natural Product Discovery: Cross-Disease Conserved Signaling Networks and a Generalizable Translational Pipeline

**DOI:** 10.3390/biology15171447

**Published:** 2026-08-24

**Authors:** Ying Li, Nana He, Mingxiang Chang, Yiwen Wang

**Affiliations:** 1 School of Pharmaceutical Science and Technology, Faculty of Medicine, Tianjin University, Tianjin 300072, China; ying_li33@tju.edu.cn (Y.L.); henanawz@163.com (N.H.); 2 Hubei Key Laboratory of Resources and Chemistry of Chinese Medicine, School of Pharmacy, Hubei University of Chinese Medicine, Wuhan 430065, China; changmingx@163.com; 3 Neurocritical Care Medicine Innovation Center, Ministry of Education, Tianjin University, Tianjin 300072, China; 4 International Joint Research Centre for Molecular Sciences, Tianjin University, Tianjin 300072, China

**Keywords:** *Drosophila melanogaster*, natural drugs screening, diabetes, kidney stones, inflammatory bowel disease, cancer, neurodegenerative diseases

## Abstract

*Drosophila melanogaster* shares approximately 75% of human disease-related genes and possesses sophisticated genetic toolkits, including GAL4/UAS, CRISPR-Cas9, and RNA interference (RNAi), making it a rapid, cost-effective, and genetically tractable *in vivo* platform for natural products (NPs) discovery. This review systematically summarizes the modeling strategies, pathological mechanisms, and therapeutic applications of *Drosophila* models for six major human diseases, including type 2 diabetes, nephrolithiasis, inflammatory bowel disease, cancer, Alzheimer’s disease, and Parkinson’s disease. Cross-disease analysis identifies five evolutionarily conserved signaling networks—IIS/PI3K/Akt/FOXO, JNK/JAK/STAT, Nrf2/Keap1, mTOR/TORC1, and IMD/Toll—as common molecular targets of bioactive NPs, providing a unified mechanistic framework for understanding their multi-target pharmacological activities and broad therapeutic potential. Critically, we propose a generalizable integrated stepwise pipeline: high-throughput fly screening of crude extracts, bioassay-guided isolation of active monomers, genetic mechanistic dissection via RNAi and mutant rescue, and layered validation in human cells and selective mammalian models. This pipeline addresses key challenges in NPs research, including the identification of bioactive constituents and mechanistic validation, while improving screening efficiency and translational potential. Overall, this review establishes a multi-disease-applicable framework linking disease modeling, conserved signaling mechanisms, and translational pharmacology, providing practical guidance for future mechanism-driven NP discovery and preclinical development using *Drosophila*. By leveraging *Drosophila* genetics to bridge evolutionary conservation and human pathology, this framework offers a powerful, paradigm-shifting strategy to accelerate mechanism-driven NP discovery and preclinical development.

## Simple Summary

Natural products from plants and traditional medicines are rich sources of potential new drugs, but finding them is often slow, expensive, and plagued by uncertainty about which ingredients are active and how they work. This review tackles these problems using *Drosophila melanogaster*—a rapid, low-cost animal model that shares most human disease genes. We systematically compare fly models of six major diseases (diabetes, kidney stones, inflammatory bowel disease, cancer, and Alzheimer’s/Parkinson’s) and make two key highlights. First, effective natural products—ranging from polyphenols and terpenoids to alkaloids and bioactive peptides—are used across these distinct etiologies, yet they consistently act on five evolutionarily conserved cell-signaling pathways—IIS/PI3K/Akt/FOXO, JNK/JAK/STAT, Nrf2/Keap1, mTOR/TORC1, and IMD/Toll immune signaling. This explains why a single herbal medicine can treat multiple conditions. Second, we propose a practical step by step pipeline: screen crude extracts in flies, isolate pure active compounds, dissect their genetic targets using fly tools, and finally validate in human cells and mice. This workflow, which is exemplified by representative nephrolithiasis studies, directly solves the major bottlenecks of unclear active ingredients and vague mechanisms. Together, this framework provides a practical roadmap for accelerating natural product discovery while reducing costs and reliance on mammalian models.

## 1. Introduction

Chronic and complex human diseases, including diabetes, nephrolithiasis, inflammatory bowel disease (IBD), tumors, and neurodegenerative disorders, remain major global health challenges [1]. Although current pharmacotherapy has achieved substantial progress, its long-term efficacy is often limited by single-target mechanisms, adverse effects, drug resistance, and inadequate therapeutic responses to multifactorial diseases [2,3]. It urgently requires the exploration of novel drug sources and efficient preclinical screening strategies [4]. The six diseases discussed in this review exhibit distinct clinical manifestations. However, they share common pathological hallmarks. These include chronic oxidative stress, persistent low-grade inflammation, metabolic dysregulation, and aberrant cell proliferation or death. These interconnected processes are governed by both transcriptional and post-translational regulatory networks that are largely conserved between *Drosophila* and humans [5,6]. Epigenetic modifications—such as DNA methylation, histone acetylation, and non-coding RNA-mediated regulation—also contribute to disease susceptibility and progression [7].

Natural products (NPs), particularly those derived from traditional Chinese medicine (TCM), possess remarkable structural diversity, multi-target pharmacological activities, and low toxicity, making them promising candidates for treating these complex disorders [8]. However, their clinical translation remains hindered by low-throughput screening, unclear bioactive constituents, and insufficient mechanistic validation, highlighting the need for rapid and cost-effective preclinical screening platforms [9,10]. The NPs evaluated in this review encompass diverse heterogeneous substances. These range from crude herbal extracts and compound Chinese medicinal formulas to purified phytochemical monomers, bioactive food-derived peptides, and probiotics. Synthetic small-molecule drugs are used solely as positive controls. These agents differ greatly in chemical complexity, experimental reproducibility, mechanistic interpretability and translational potential [11]. Crude extracts and multi-herb formulas exert synergistic effects but hinder precise target identification, while purified phytochemicals and peptides enable clear target validation yet lack multi-pathway synergy; probiotics act indirectly by regulating gut microbiota, distinct from small-molecule compounds [12,13]. Therapeutically active NPs also cover diverse chemical classes with distinct bioactivities. Polyphenols (flavonoids, phenolic acids, stilbenes) show antioxidant, anti-inflammatory, and metabolic-regulatory effects. Terpenoids often display anti-tumor, neuroprotective, and anti-obesity activities [12]. Alkaloids modulate insulin signaling, suppress tumors, and alleviate renal injury. Plant- or fungus-derived peptides and polysaccharides confer prebiotic and immunomodulatory benefits [14]. Such chemical diversity underlies the multi-target pharmacological profiles of NPs, allowing them to target the multifactorial pathogenesis of complex diseases [15].

**Figure 1:**
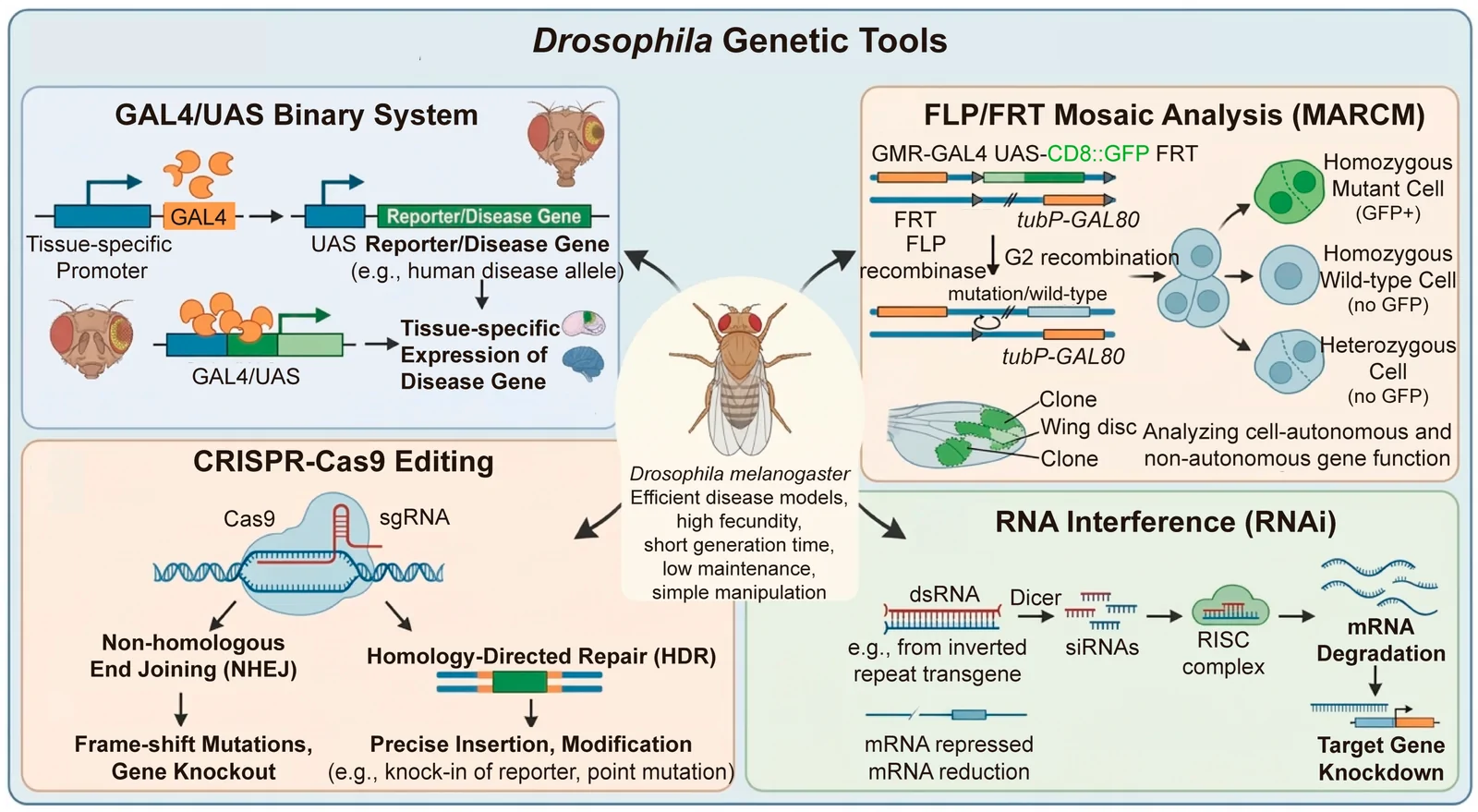
Major genetic tools for *Drosophila melanogaster* research. The central panel lists key merits of fruit fly as a disease model. Top-left: GAL4/UAS binary system for tissue-specific overexpression of target genes or reporters. Top-right: MARCM (FLP/FRT mosaic labeling) to trace homozygous mutant cell clones and distinguish cell-autonomous/non-autonomous gene phenotypes in wing discs or other tissues. Bottom-left: CRISPR-Cas9 editing via NHEJ-mediated knockout or HDR-mediated precise genome modification. Bottom-right: RNAi pathway mediates targeted mRNA degradation to induce gene knockdown. When combined with Gal4/UAS, it can achieve the knockdown of specific gene expression within specific time periods and specific tissues.

*Drosophila melanogaster* is a classic eukaryotic model species with a century-long research history. It belongs to the genus *Drosophila* of the *Diptera* order. Among the more than 1500 described *Drosophila* species, *D. melanogaster* is phylogenetically close to *D. simulans* and *D. suzukii*. Together with other congeneric species, it provides diverse and powerful invertebrate research platforms for biological and biomedical studies [16,17]. *Drosophila melanogaster*, as a classic and mature eukaryotic model organism, possesses unparalleled superiorities in preclinical drug screening and pathological mechanism research. Approximately 75% of human disease-related genes have homologous counterparts in the *Drosophila* genome, ensuring high conservation of core signaling pathways governing metabolism, inflammation, cell proliferation, apoptosis, and neural development [18,19,20].Coupled with its short life cycle, low maintenance cost, and powerful genetic toolkits—including the GAL4/UAS system, FLP/FRT-mediated mosaic analysis, CRISPR-Cas9 genome editing, and RNA interference (RNAi)—(Figure 1) *Drosophila* enables efficient disease modeling, high-throughput phenotypic screening, toxicity evaluation, and mechanistic investigations that complement conventional mammalian models [21,22,23]. Furthermore, *Drosophila melanogaster* provides a practical *in vivo* model. It can be used to study co-exposure and joint metabolism of natural compounds from distinct chemical classes. Flavonoids, terpenoids, phenolic acids, and alkaloids mutually regulate multiple pathways, altering the metabolic fate and bioactivity of individual components [11,24]. Its genetic tractability and high-throughput capacity enable efficient screening of synergistic or antagonistic combinatorial effects [24,25]. Although combined protective effects of multi-class phytochemicals have been demonstrated in *Drosophila* disease models, direct investigations into their joint metabolic crosstalk remain limited [24]. Moreover, the model has low cost, ease of use, and ability to recapitulate multiple comorbid phenotypes. These features make it highly suitable for studying compound metabolism under complex pathological conditions. It offers great potential for mechanism-driven natural product combination research [15,25].

In recent years, *Drosophila* disease models have been increasingly applied to explore the therapeutic potential of natural products against diabetes, nephrolithiasis, IBD, cancer, Alzheimer’s, and Parkinson’s diseases, with mature modeling strategies now available for all six conditions [10,26,27,28,29,30]. However, existing reviews are largely confined to single diseases and rarely integrate conserved pharmacological mechanisms or generalizable translational strategies across different pathologies [24,30,31].

The overarching goal of this review is threefold. First, we systematically summarize *Drosophila*-based natural product research across six major diseases as a comprehensive reference. Second, we identify five conserved signaling pathways commonly targeted by natural products, establishing a unified mechanistic framework for their multi-target and cross-disease efficacy. Third, we propose a translational pipeline using *Drosophila* to resolve key bottlenecks in natural product discovery, including active constituent identification, target validation, and mechanism elucidation. This framework links evolutionary conservation to human pathology, accelerating natural product translation and reducing reliance on costly mammalian models.

## 2. Research Progress of Natural Products in the Treatment of Six Major Diseases Based on *Drosophila* Models

### 2.1. Diabetes Models

Diabetes mellitus (DM) is a chronic metabolic disorder characterized by impaired glucose homeostasis and persistent hyperglycemia, leading to systemic metabolic dysfunction and multiple complications, including nephropathy, retinopathy, neuropathy, and cardiovascular disease [32,33,34,35,36,37,38]. DM comprises type 1 diabetes (T1DM), caused by insulin deficiency following pancreatic *β*-cell destruction, and type 2 diabetes (T2DM), which accounts for 90–95% of cases and is characterized by insulin resistance and *β*-cell dysfunction [39,40]. Despite considerable therapeutic advances, current antidiabetic drugs remain limited by adverse effects and incomplete long-term efficacy, highlighting the need for safer and more effective therapeutic agents [41].

The first *Drosophila* T1DM-like model was established in 2002 by genetic ablation of insulin-producing cells (IPCs), the functional counterparts of mammalian pancreatic β-cells [42]. This approach enables investigation of insulin deficiency phenotypes and screening of compounds that promote insulin-independent glucose uptake. However, studies investigating natural product interventions in T1DM *Drosophila* models remain extremely limited.

In *Drosophila*, T2DM is primarily modeled through dietary interventions. A high-sugar diet (HSD) for seven days induces peripheral insulin resistance, compensatory DILP over-secretion, elevated hemolymph glucose and trehalose, and fat-body triglyceride accumulation [22,43,44]. Prolonged HSD further impairs IPC function, mirroring human T2DM progression from early insulin resistance to late β-cell failure [45]. Similarly, a high-fat diet (HFD)—often composed of coconut oil or other saturated fats—triggers insulin resistance, ectopic fat storage, and persistent hyperglycemia [46,47]. These obesogenic substances adopted in HSD and HFD cover sucrose, fructose, as well as saturated lipids including coconut oil and lard. These diet-induced models are suitable for high-throughput drug screening, whereas genetic models facilitate mechanistic studies.

Although rodent models remain indispensable, their high cost and low throughput limit large-scale screening of natural products [48,49]. Owing to the remarkable conservation of insulin signaling, *Drosophila* has emerged as an attractive complementary model (Table S1) [50,51,52,53,54,55,56,57,58,59,60,61,62,63]. The fly insulin signaling pathway, including insulin-like peptides (DILPs), the insulin receptor (InR, orthologous to the human IR/IGF-1R), and the downstream Chico/PI3K/Akt/TOR/dFOXO cascades, is highly homologous to that of mammals and regulates glucose and lipid homeostasis [43,64]. Together with its functional fat body, which integrates liver- and adipose-like metabolic functions, *Drosophila* provides an efficient platform for investigating diabetic mechanisms and high-throughput screening of antidiabetic natural products [22].

#### 2.1.1. Common Diabetic Modeling Methods in *Drosophila*

The first *Drosophila* T1DM-like models were established in 2002 by genetic ablation of insulin-producing cells (IPCs), the functional counterparts of mammalian pancreatic *β*-cells that secrete DILP2/3/5 to regulate glucose homeostasis [42,65].

T2DM models are more widely used due to their higher clinical relevance. In wild-type flies, a high-sugar diet (HSD) for seven days induces peripheral insulin resistance, compensatory DILP over-secretion, elevates hemolymph glucose and trehalose, fat-body triglyceride accumulation, and disrupts glucose-lipid metabolism [22,43,44]. Prolonged HSD further impairs IPC function and responsiveness [45], mirroring human T2DM progression from early peripheral insulin resistance to late *β*-cell failure. Similarly, high-fat diet (HFD) feeding triggers insulin resistance, excess fat storage, and persistent hyperglycemia, reproducing a full spectrum of T2DM-like metabolic phenotypes [46,47], mirroring human T2DM progression from early peripheral insulin resistance to late *β*-cell failure [66,67]. Diet-induced T2DM models are suitable for drug screening, whereas genetic models facilitate mechanistic studies. Detailed modeling strategies and assessment indices are summarized in Table S2.

#### 2.1.2. Research Progress and Mechanism of NPs in Diabetes Treatment

**Figure 2:**
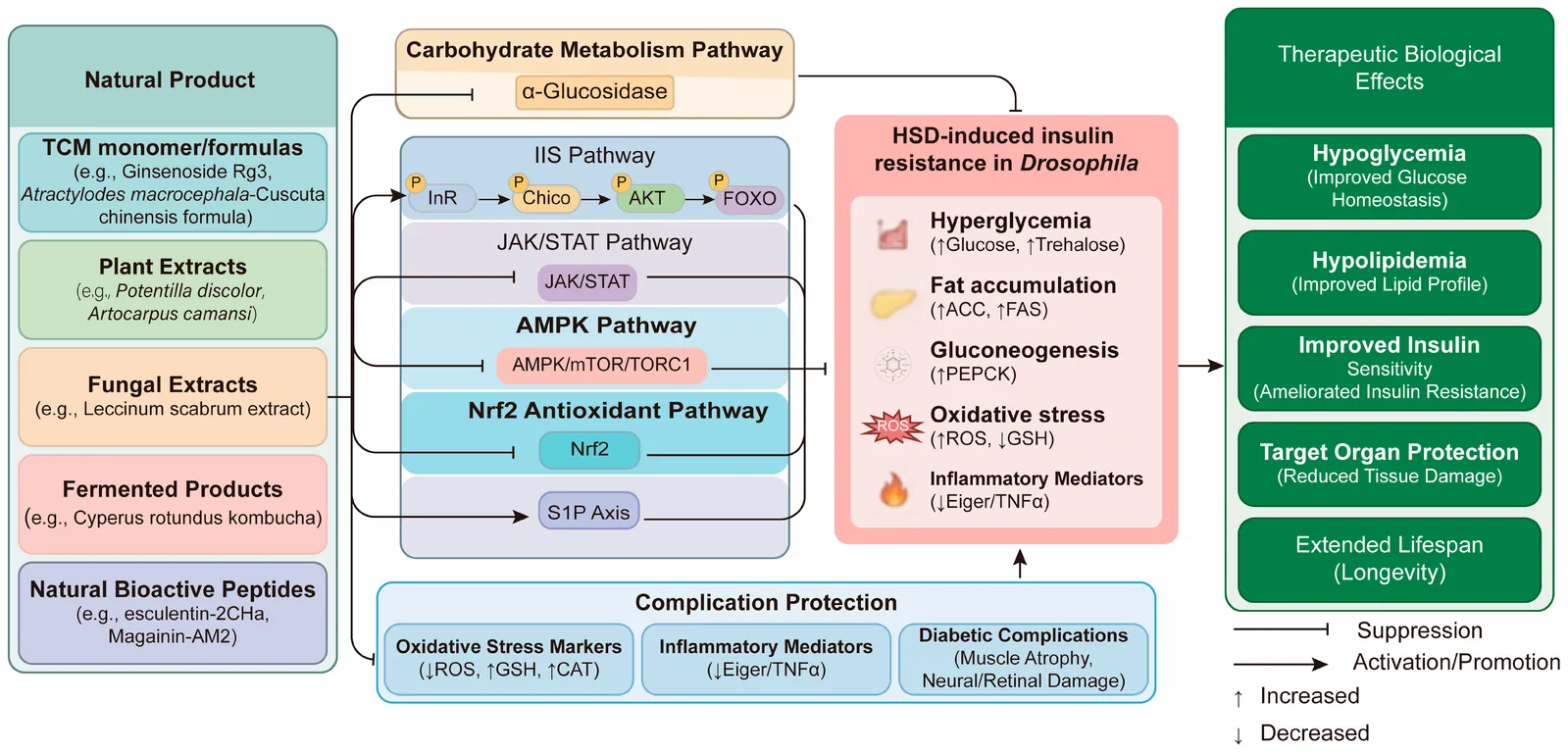
Mechanisms of natural products alleviating HSD-induced insulin resistance in *Drosophila*. Natural products including TCM monomers, plant/fungal extracts, bioactive peptides and probiotics exert multi-target effects via carbohydrate hydrolase inhibition and modulation of IIS, JAK/STAT, adenosine monophosphate activated protein kinase (AMPK) and Nrf2 pathways to suppress oxidative stress and inflammation, thereby restoring glycolipid homeostasis, insulin sensitivity and tissue integrity.

Studies using *Drosophila* diabetes models have demonstrated that numerous NPs exert anti-diabetic effects by maintaining glycolipid homeostasis in a synergistic, multi-target and multi-pathway manner. The central pathological mechanism underlying diabetic metabolic disorders is the dysregulation of the IIS-PI3K-Akt-FOXO axis. This axis interacts extensively with several key downstream or parallel pathways, including the c-Jun N terminal kinase-Janus kinase signal transducer and activator of transcription (JNK-JAK/STAT) inflammatory pathway, mTOR/TORC1 energy-sensing pathway, carbohydrate metabolism pathway, and oxidative stress-related CncC/Nrf2 pathway, forming a complex regulatory network (Figure 2).

(1) The IIS-PI3K-Akt-FOXO cascade is the core conserved glucose metabolism pathway in flies and humans, serving as a validated therapeutic target for metabolic disorders and the most frequently modulated signaling module of anti-diabetic natural products (NPs) in *Drosophila* (Figure 2). Physiologically, *Drosophila* brain IPCs secrete ILP2/3/5 to bind peripheral InR, triggering Chico phosphorylation and PI3K-Akt activation. Active Akt phosphorylates FOXO to sequester it in the cytoplasm and suppress gluconeogenic/lipogenic gene transcription [43,66]. HSD disrupts this homeostasis: elevated ILP2 alongside excess insulin antagonist Imp-L2 competitively block InR, blunting IIS signaling, driving sustained FOXO nuclear entry, and over-activating glucose/lipid synthesis pathways to induce systemic insulin resistance and hyperglycemia [44,45,68]. NPs and herbal medicines that boost IIS-PI3K-Akt-FOXO signaling restore insulin sensitivity and normalize disordered glycolipid metabolism in diabetic flies.

Notably, NPs regulate this axis via two distinct modes: direct core cascade targeting and indirect crosstalk through parallel pathways. Direct regulators act on IIS core components: *Atractylodes macrocephala-Cuscuta chinensis* extract increases PI3K/Akt phosphorylation, enhances glucose transporter 1/3-dependent glucose uptake and blocks FOXO nuclear translocation [69]. Sanghuang Tongxie Formula relieves insulin resistance via PI3K/Akt modulation [70]. *Artocarpus camansi* extract balances ILP2/InR/Imp-L2 expression to stabilize glycemia [71]. *Spondias mombin* stem bark extract modulates ILP-2/InR signaling [72]. Several soy isoflavone monomers, including genistein, daidzein, biochanin A, and glycitein, have been reported to exert insulin-sensitizing effects through modulation of the PI3K/AKT signaling pathway in various mammalian models [73,74,75]. In *Drosophila*, a soy extract rich in hydroxylated isoflavones has demonstrated antidiabetic properties [76], suggesting a potential conserved role of this insulin signaling axis in flies. Crocin can also reverse insulin resistance by regulating IIS/Akt metabolic signaling [77]. Indirect modulators act through parallel pathways. *Potentilla discolor* extract suppresses JAK/STAT signaling, downregulates Imp-L2 and Socs36E, and rescues PI3K/Akt activity [78,79]. These findings demonstrate the resolving power of *Drosophila* models for discriminating between direct insulin sensitization (e.g., soy isoflavones) and indirect pathway modulation (e.g., *Potentilla discolor* Bunge via JAK/STAT).

(2) JNK-JAK/STAT axis. HSD disrupts gut microbiota (reduced *Acetobacter*/*Lactobacillus*), activating intestinal JNK, which triggers Upd3 (IL-6 homolog) secretion and systemic JAK/STAT activation, driving Imp-L2/Socs36E expression and insulin resistance [68,78,80,81]. Direct supplementation with *Acetobacter* and *Lactobacillus* blocks JNK-JAK/STAT pathway activation at its source by restoring a healthy microbiota composition [80]. This gut-derived inflammatory cascade is evolutionarily conserved and represents a promising target for microbiota-modulating natural products. The water extract of *Potentilla discolor* can directly target and inhibit the JAK/STAT signaling pathway without altering the gut microbiota [78].

(3) AMPK/mTOR/TORC1 pathway. The mTOR/TORC1 axis is a core energy sensor integrating nutrients, insulin signals and metabolism, and converges with IIS-PI3K-Akt to couple insulin sensitivity and lipid synthesis. Chronic hyperglycemia and insulin resistance over-activate TORC1, which upregulates the lipogenic transcription factor HLH106 (*Drosophila* SREBP), lipogenic genes encoding acetyl CoA carboxylase (ACC) and fatty acid synthase, plus gluconeogenic phosphoenolpyruvate carboxykinase (PEPCK); collectively driving ectopic lipid deposition and whole-body metabolic dysfunction.

Many NPs replicate this metabolic modulation through the conserved AMPK-TORC1 cascade. Berberine (from *Rhizoma Coptidis*) acts as a classic AMPK agonist: AMPK activation inhibits TORC1, represses *pepck*/*acc* transcription, and relieves hyperglycemia, insulin resistance and excessive lipogenesis in diabetic flies [82]. Similarly, the ethyl acetate extract of *Atriplex halimus* L. modulates HLH106 to normalize glycolipid metabolism. Under insulin-sufficient states, Akt-dependent TORC1 boosts HLH106-driven lipogenesis, while nuclear FOXO represses HLH106 transcription during insulin resistance. This herbal extract blocks TORC1-mediated HLH106 activation and mitigates lipid-induced IIS dysfunction, coordinately correcting systemic insulin resistance and glycolipid disturbance [83].

Other auxiliary regulatory nodes have also been validated. Sphingosine-1-phosphate (S1P) signaling acts as an upstream lipid rheostat balancing mTORC1 and AMPK activity [84,85,86]. *Lanhuashen* extract improves glycolipid homeostasis via the S1P axis, illustrating an underexplored regulatory route [87]. DPP4 inhibition is another anti-T2DM mechanism for natural products. DPP4 degrades insulinotropic GLP-1 and GIP to restrict postprandial insulin release and impairs Akt signaling; DPP4 blockade restores Akt activity and rebalances anabolic mTORC1 and catabolic AMPK pathways [88]. Walnut (*Juglans sigillata*)-derived bioactive peptides represent a typical dual-functional natural DPP4 inhibitor: core peptide sequences (e.g., LPFA, FPAG, LPLLR) competitively occupy the catalytic pocket of DPP4 [89,90,91,92].

(4) Carbohydrate metabolism pathway. In the *Drosophila* T2DM model, excessive carbohydrate intake over-activates intestinal α-amylase and α-glucosidase. This accelerates starch and disaccharide hydrolysis, causes postprandial hyperglycemia, exacerbates fat-body glycolipid accumulation, and ultimately aggravates insulin resistance [93,94]. Multiple natural products suppress key carbohydrate hydrolases to alleviate diabetic abnormalities. Extracts of *Artocarpus camansi* and *Solanum anguivi*, as well as *Myrica rubra* proanthocyanidins, inhibit α-amylase and α-glucosidase and reduce fat-body lipid deposition [71,91,93]. *Carica papaya* leaf extract achieves hypoglycemic effects via dual regulation: it blocks α-amylase to limit glucose release and enhances IIS-PI3K-Akt signaling to promote glucose uptake and utilization [94]. Kombucha fermented from *Cyperus rotundus* L. improves diabetic phenotypes through antioxidant activity and carbohydrate hydrolase inhibition [95], with contributions from yeast-derived ergosterol (pro-vitamin D_2_) and microbial tryptophan-derived indolic antioxidants; these bioactive metabolites modulate redox homeostasis, and conserved metabolic cascades in *Drosophila* facilitate mechanistic exploration of their anti-hyperglycaemic actions [96,97].

This dual action, reducing postprandial glucose absorption while improving insulin sensitivity, is a common feature of many plant-derived extracts and may explain their efficacy in *Drosophila* HSD models. In addition, *in vitro* research indicated that flavonoids (luteolin-7-*O*-diglucuronide, apigenin-7-*O*-diglucuronide) and rosmarinic acid from *Perilla frutescens* var. acuta possess anti-adipogenic and thermogenic activities [98]. Notably, perillartine, a natural terpenoid sweetener approximately 2000-fold sweeter than sucrose, has been validated *in vivo* to alleviate high-fat diet-induced lipid metabolism disorders in broiler chickens [99]. Sweeteners exert divergent and hormetic effects on gut microbiota. They alter microbial composition, short-chain fatty acid production, and gut barrier function. Their impacts on sugar and fat metabolism have been mechanistically investigated in *Drosophila*. These investigations involve pathways related to energy sensing and metabolic gene regulation [100,101,102]. Yet whether perillartine regulates gut microbiota and modulates carbohydrate/lipid homeostasis through conserved metabolic signaling in *Drosophila* diabetic models remains unexplored, offering a mechanistically novel direction for future research.

(5) Nrf2/CncC antioxidant pathway. Chronic hyperglycemia elevates reactive oxygen species (ROS), malondialdehyde and nitric oxide, while suppressing antioxidant enzymes including superoxide dismutase (SOD) and catalase. Alleviating nutrient-induced oxidative stress relieves oxidative damage-mediated insulin resistance and halts diabetic complication progression. Multiple natural products exert anti-diabetic effects via activating the Nrf2/CncC antioxidant pathway. Sesamin upregulates SOD and catalase, reduces cardiac ROS, and ameliorates diabetic cardiomyopathy [103]. Caffeic acid and *Cyperus rotundus* L. Kombucha dual-functionally activate Nrf2-dependent antioxidant defenses and inhibit α-amylase/α-glucosidase, lowering postprandial glucose absorption [104]. Oxidative stress is a universal pathological hallmark of metabolic disorders and a common downstream ameliorative effect of natural products and herbal medicines, rather than a specific therapeutic target for diabetes. Thus, antioxidant regulation should be analyzed together with core glycolipid metabolic pathways to fully illustrate the holistic advantages of herbal treatments. The anti-diabetic mechanisms of natural products are summarized in Table S3.

In addition, certain terpenoid alkaloids such as solanines, typical glycoalkaloids from the *Solanaceae* family, also show clear hormetic profiles. Low doses may trigger protective antioxidant responses via Nrf2-mediated pathways and reduce genotoxicity, whereas high doses lead to overt cytotoxicity, genotoxicity, and general toxicity [105,106]. Systematic studies in *Drosophila* remain limited. However, this model has well-established antioxidant and DNA damage response pathways. Together with available genotoxicity assays, these features render *Drosophila* a highly suitable system for dissecting the dose-dependent biphasic effects of these terpenoid compounds. These effects can be studied in the context of carbohydrate metabolism and oxidative stress [107,108].

#### 2.1.3. Advantages and Limitations of *Drosophila* DM Models

Compared with rodent diabetic models, *Drosophila* T2DM models feature rapid modeling, short experimental cycles and low cost for high-throughput screening. However, flies lack complete mammalian endocrine axes and rely on trehalose as circulating sugar, failing to fully recapitulate human systemic endocrine complications. Moreover, the ad libitum feeding fails to achieve precise quantitative drug exposure, which brings large deviations to dose-effect analysis. Inconsistent results across studies are not uncommon, and many compounds effective in flies fail to reproduce efficacy in mammals, partly due to incompatible pharmacokinetics and uncertain cross-species dose translation. Future research needs to combine single-gene interference, pure monomer comparison and quantitative injection administration to clarify the hierarchical regulatory relationship of multi-target natural compounds.

### 2.2. Kidney Stone Models

*Drosophila* encounters calcium oxalate (CaC_2_O_4_) in its natural habitat, as needle-shaped CaC_2_O_4_ raphide crystals are widely distributed across numerous plant species; dietary oxalate from decaying fruits, leaves and plant sap constitutes a constant natural exposure source for wild flies [109]. This ecological background strengthens the biological relevance of deploying *Drosophila* as a kidney stone model [110]. Accordingly, this model enables experimental modulation not only of artificial lithogenic conditions, but also physiological exposure to oxalate ions as a naturally occurring environmental and dietary compound.

Calcium oxalate nephrolithiasis (CaOxN), the predominant form of kidney stones (~80%), is a recurrent urological disorder driven by CaOx crystal deposition and renal tubular injury, with poorly understood molecular mechanisms [111]. Because *Drosophila* Malpighian tubules are functionally analogous to mammalian renal tubules, flies faithfully recapitulate CaOx crystal deposition and renal tubular injury under lithogenic conditions [112]. Their transparent body and simple tubular architecture facilitate direct visualization of crystal formation, making *Drosophila* a powerful model for mechanistic studies and high-throughput screening of anti-urolithic natural products.

#### 2.2.1. Common Nephrolithiasis Modeling Methods in *Drosophila*

The most classic model is the high-oxalate diet-induced calcium oxalate (CaOx) stone model. Sodium oxalate feeding in female flies stably induces CaOx crystal deposition in Malpighian tubules without affecting lifespan, making it ideal for high-throughput screening of anti-lithic compounds. Additionally, dietary ethylene or hydroxyproline supplementation promotes endogenous oxalate overproduction and mimics hyperoxaluria pathology [113,114,115]. A hereditary CaOx stone model was established via Malpighian tubule-specific knockdown of *dAGXT*, the fly homolog of human primary hyperoxaluria type 1 gene *AGXT*. This intervention blocks glyoxylate transamination to trigger spontaneous endogenous oxalate accumulation and stable stone formation without exogenous lithogenic agents, supporting mechanistic research and precision drug development for hereditary nephrolithiasis [114]. The detailed modeling methods and evaluation indicators of *Drosophila* kidney stone models are presented in Table S4.

#### 2.2.2. Research Progress and Mechanism of NPs in Nephrolithiasis Treatment

**Figure 3:**
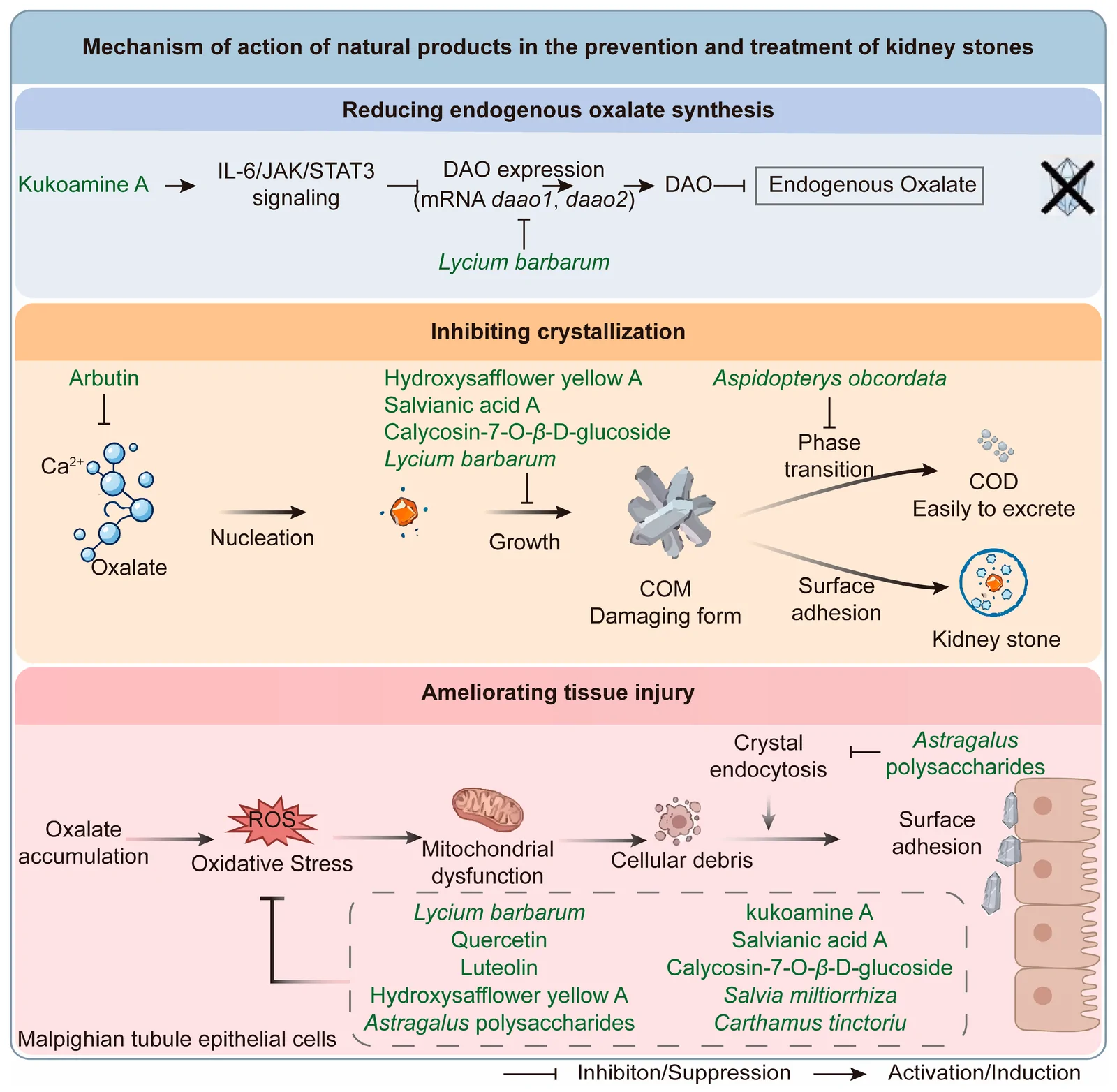
Schematic illustration of natural products-mediated anti-urolithiasis mechanisms in *Drosophila* nephrolithiasis models. CaOx stones, the most common clinical kidney stone subtype, develop via crystal nucleation, growth, aggregation and renal retention. Natural products relieve nephrolithiasis through three core strategies: limiting lithogenic substrate supply, suppressing crystal formation, and mitigating tubular tissue injury.

Evidence from *Drosophila* models indicates that the anti-urolithiasis strategies of natural products converge on three mechanistic axes: limiting substrate supply, directly inhibiting crystallization, and ameliorating tissue injury (Figure 3).

(1) Limiting Substrate Supply. CaOx crystal formation occurs when tubular oxalate and calcium concentrations exceed solubility thresholds, making ion depletion an upstream preventive strategy [116]. Endogenous synthesis produces over 80% of human oxalate, with D-amino acid oxidase (DAO) as the key rate-limiting enzyme [117]. Kukoamine A from *Lycium chinense* downregulates DAO expression via IL-6/JAK/STAT3 activation [118]. Consistently, *Lycium barbarum* aqueous extract markedly suppresses *daao1* and *daao2* transcription and eliminates crystal deposition in most Malpighian tubules [119]. Since humans cannot degrade oxalate intrinsically, dietary oxalate relies on exogenous clearance pathways [120]. Additionally, gut microbiota-modulated unconjugated bilirubin regulates renal CaOx deposition, representing a novel therapeutic target for oxalate-related nephropathy [121].

(2) Inhibiting Crystallization. Multiple stages of CaOx crystallization, including nucleation, growth, phase transition and surface adhesion, can be therapeutically intervened [122]. A screening of 360 natural compounds identified arbutin, a small molecule that chelates both Ca^2+^ and oxalate. At 1 mM, arbutin nearly abolishes CaOx crystallization and disrupts crystal morphology. Notably, arbutin exerts stable oxalate-binding activity independent of pH, overcoming the pH-dependent limitation of potassium citrate [123]. Regulation of crystal phase transformation also alleviates nephrolithiasis. Damaging large calcium oxalate monohydrate (COM) crystals can convert them into easily excretable calcium oxalate dihydrate (COD) crystals [118,124]. An insulin-type fructan AOFOS purified from *Aspidopterys obcordata* inhibits large crystals (>200 μm^2^) and increases microcrystals (<10 μm^2^) in fly Malpighian tubules, reducing crystal size and promoting COM-to-COD transformation [125]. In addition, the anti-crystallization effect of phenolic compounds may also arise from their strong calcium-chelating ability. Phenolic acids such as chlorogenic acid and rosmarinic acid contain carboxylate and hydroxyl groups that bind Ca^2+^ more tightly than oxalate [126]. By sequestering free calcium ions, they reduce Ca^2+^ availability for CaOx nucleation and growth, representing an additional mechanism by which polyphenol-rich natural products inhibit urolithiasis [127]. For example, hydroxysafflower yellow A, salvianic acid A, and calycosin-7-O-*β*-D-glucoside can suppress CaOx formation and modulate crystal growth [126,128].

(3) Ameliorating Tissue Injury. Improving renal tissue tolerance and repair can mitigate stone damage even when crystallization cannot be completely blocked. Oxalate toxicity induces mitochondrial dysfunction and excessive ROS accumulation, and resulting cellular debris further facilitates crystal adhesion and deposition [129,130]. *Astragalus membranaceus* extract reduces EG-induced CaOx crystallization and significantly rescues the shortened lifespan of model flies; ex vivo assays exclude direct crystal dissolution, verifying tissue protection as its dominant mechanism [131]. *Astragalus* polysaccharides exert comprehensive protective effects by inhibiting crystal endocytosis, reducing crystal adhesion, and eliminating free radicals [132,133,134]. Similarly, *Lycium barbarum* lowers ROS levels and normalizes the expression of antioxidant genes *sod1*, *sod2* and *cat* [119]. The herbal flavonoids quercetin and luteolin also reverse oxalate-induced ROS overproduction and restore cellular redox homeostasis in Malpighian tubules [135].

Natural products treat nephrolithiasis via typical multi-target mechanisms. Kukoamine A reduces endogenous oxalate synthesis by downregulating the rate-limiting enzyme DAO and relieves oxidative tissue injury. Kukoamine A, hydroxysafflower yellow A, salvianic acid A, and calycosin-7-O-*β*-D-glucoside exert their anti-nephrolithiasis effects in *Drosophila* mainly by alleviating oxidative stress, and their efficacy has also been validated in mouse models. This supports the clinical translational potential of the *Drosophila* nephrolithiasis model [118,128]. Cumulative evidence demonstrates that herbal compound combinations exert superior efficacy over single monomers. For example, mixed aqueous extracts of *Astragalus membranaceus*, *Salvia miltiorrhiza* and *Carthamus tinctorius* nearly completely inhibit crystal formation in *Drosophila* models, with stronger effects than single or combined use of quercetin and luteolin [135]. The anti-nephrolithiasis mechanisms of natural products are summarized in Table S5.

#### 2.2.3. Advantages and Limitations of *Drosophila* Nephrolithiasis Models

*Drosophila* Malpighian tubules contain merely two cell types and are optically transparent, enabling direct real-time observation of crystal deposition without complex tissue sectioning. Still, this simple renal structure lacks glomeruli and complete tubular segmentation distinct from mammalian kidneys. In addition, most studies merely focus on crystal inhibition but overlook oxalate-triggered oxidative stress and inflammation. Moreover, existing assays generally evaluate single kidney stone subtypes without cross-comparisons across calcium oxalate, uric acid and calcium phosphate models, restricting subtype-specific translational application, highlighting the demand for multi-index quantitative systems in future screening.

### 2.3. IBD Models

Inflammatory bowel disease (IBD), including ulcerative colitis and Crohn’s disease, is a chronic relapsing inflammatory disorder driven by complex interactions among genetic, environmental, immune, and microbial factors, while current therapies remain limited by incomplete efficacy, adverse effects, and high costs [136,137]. In humans, nucleotide-binding oligomerization domain-containing protein 2 (NOD2) mutations confer the strongest genetic risk for Crohn’s disease [138]. NOD2 acts as an intracellular sensor that detects bacterial muramyl dipeptide and initiates innate immune responses [138]. Although *Drosophila* lacks a direct NOD2 ortholog, Imd signaling triggered by peptidoglycan receptors (PGRP-LC/PGRP-LE) recapitulates core features of NOD2-mediated intestinal immune surveillance, establishing flies as a valuable model for bacteria-induced intestinal inflammation [30]. The *Drosophila* midgut shares conserved epithelial structure, stem cell regulation, and innate immune pathways with the human intestine. It serves as a valuable IBD model. It enables focused investigation of innate immunity and high-throughput screening of anti-inflammatory compounds. However, it lacks adaptive immunity and T/B cells [28].

#### 2.3.1. Common IBD Modeling Methods in *Drosophila*

*Drosophila* IBD models are mainly established via four approaches: chemical induction, chemotherapy-induced intestinal damage, gene editing, and microbiota manipulation [28,32].

Chemical induction is the most prevalent method for *Drosophila* IBD modeling. It disrupts intestinal epithelial barrier integrity via toxicants to trigger inflammatory cascades and repair disorders, primarily mimicking acute ulcerative colitis [30,139]. The classic inducers, DSS and sodium dodecyl sulfate (SDS), are administered to adult flies for 48–72 h, stably recapitulating key pathological features, including intestinal edema, epithelial apoptosis, intestinal shortening, imbalance of intestinal flora, and barrier leakage [140,141]. Combined Epstein–Barr virus (EBV) DNA treatment aggravates hindgut inflammation by activating innate immune pathways, simulating virus-induced IBD exacerbation and enriching model pathological phenotypes [28,140,142]. Additionally, paraquat feeding induces intestinal reactive oxygen species (ROS) overaccumulation to establish an oxidative stress-dependent IBD model, supporting mechanistic research on antioxidant natural products [28].

Chemotherapy-induced intestinal injury models simulate clinical chemotherapy-associated intestinal mucositis and barrier damage, expanding the application scope of *Drosophila* IBD models [143]. The commonly used cytarabine (Ara-C) triggers intestinal ROS burst, innate immune activation and epithelial apoptosis, leading to typical IBD-like symptoms such as intestinal shortening, imbalance of intestinal flora and barrier dysfunction [143].

Gene editing models utilize CRISPR/Cas9 and RNA interference (RNAi) to modify human IBD-risk homologous genes and inflammatory pathways, precisely simulating hereditary IBD [32,139]. Knockout of autophagy-related genes *Atg16* (WD40 domain mutants *Atg16MI* and *Atg16d67*) or *Rab19* impairs EE cell differentiation and downregulates Slit/Robo signaling, inducing spontaneous chronic intestinal inflammation that mimics core phenotypes of human ATG16L1 mutation-related Crohn’s disease [144]. Targeted activation of the immune deficiency (IMD) pathway simulates Gram-negative bacterial infection-mediated intestinal inflammation, while *GlcAT-S* silencing disrupts the intestinal mucus barrier and causes spontaneous epithelial damage [140].

Gut microbiota dysbiosis models recapitulate the “microbiota imbalance-inflammation amplification” cycle of human IBD via directional regulation of intestinal microbial composition [28]. One strategy eliminates fly intrinsic microbiota with broad-spectrum antibiotics, followed by colonization with pathogenic bacteria including *Pseudomonas aeruginosa*, *Enterococcus faecalis* and *Erwinia carotovora* subsp. *carotovora* 15 (Ecc15), inducing microbial dysbiosis, abnormal lipid droplet accumulation, excessive antimicrobial peptide production and sustained chronic intestinal inflammation [145]. Genetically, *Atg16* WD40 domain mutation genetically induces Enterobacteriaceae overproliferation, disrupts microbial homeostasis and triggers inflammation, which faithfully mimics mucosal dysbiosis in human IBD patients [144]. Detailed modeling protocols and evaluation indexes are summarized in Table S6.

#### 2.3.2. Research Progress and Mechanism of NPs in IBD Treatment

**Figure 4:**
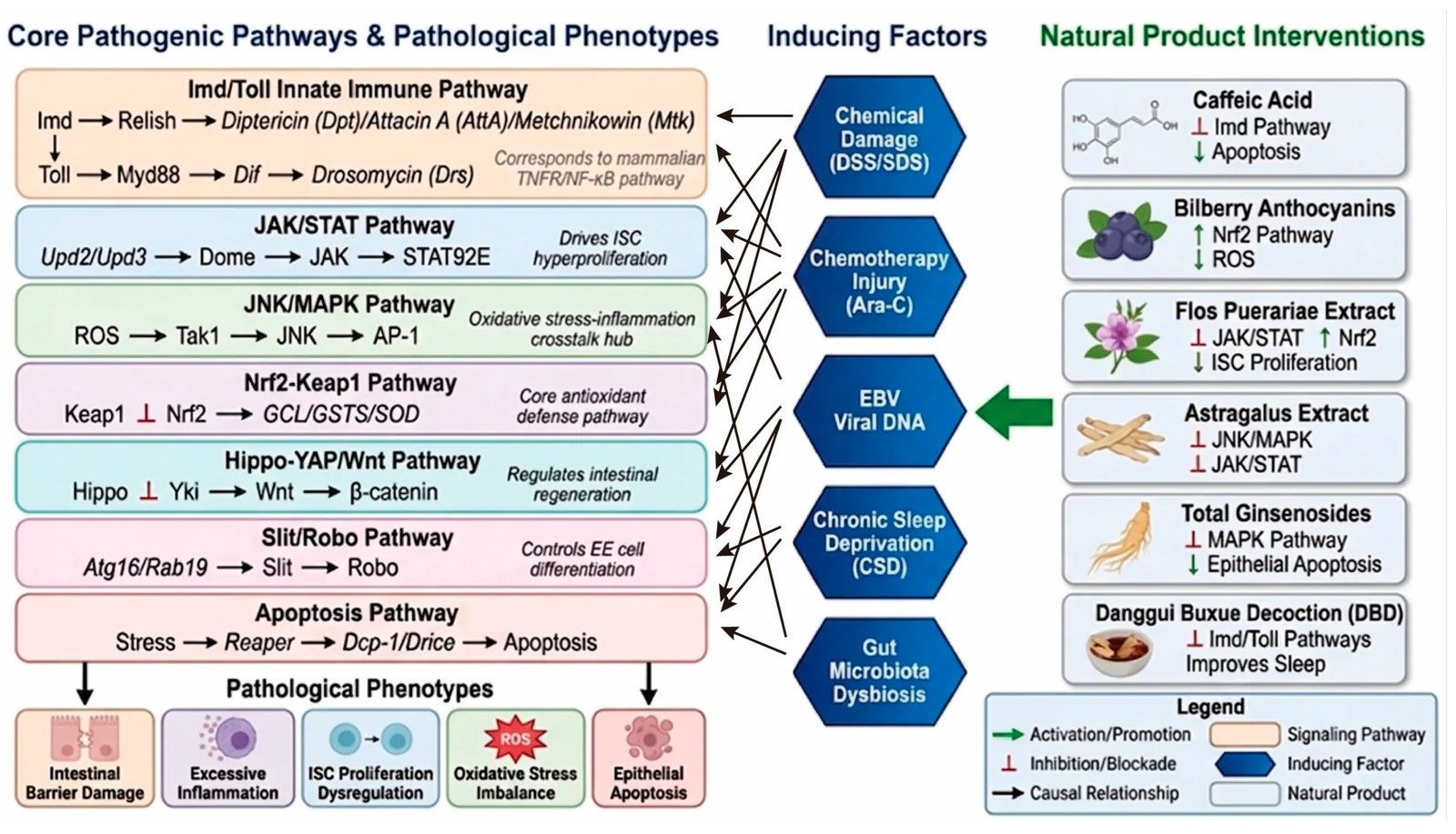
Flies IBD core regulatory mechanism and the natural medicine intervention pathway chart. On the left side are the core pathogenic pathways (Imd/Toll innate immune pathway, JAK/STAT, JNK/MAPK, Nrf2-Keap1, Hipo-YAP/Wnt, Slit/Robo, apoptosis pathway) and pathological phenotypes of *Drosophila* IBD. On the right side are the intervention targets and pathways of natural medicines (such as caffeic acid, bilberry anthocyanins, kudzu flower extract, astragalus extract, total ginsenosides of ginseng, etc.). The causes in the middle include dysbiosis, sleep deprivation, chemical/chemotherapy damage, etc.

*Drosophila* IBD pathogenesis stems from five key events: dysregulated intestinal stem cells (ISCs) proliferation and differentiation, aberrant innate immune activation, oxidative stress imbalance, enhanced epithelial apoptosis, and disrupted microbiota-inflammation crosstalk. Natural products can ameliorate these pathological alterations via multi-target and multi-pathway synergistic regulation (Figure 4).

(1) IMD/NF-κB pathway. The IMD pathway, homologous to mammalian TNFR/NF-κB signaling, serves as the core intestinal pro-inflammatory pathway. Multiple IBD inducers (DSS, EBV-DNA, chronic sleep deprivation, Ara-C) strongly activate IMD signaling and upregulate antimicrobial peptides including PGRP-SB1, Diptericin, AttA, AttB and Mtk [142,143,146]. Numerous natural products and herbal formulas exert anti-inflammatory effects by targeting this pathway. Caffeic acid suppresses excessive IMD activation, downregulates related antimicrobial peptides, and alleviates CSD- and DSS-induced intestinal damage by inhibiting abnormal ISC proliferation and epithelial apoptosis [147]. Luteolin blocks IMD/NF-κB cascades to reduce antimicrobial peptide levels, while ursolic acid inhibits NF-κB signaling and normalizes ISC proliferation. Safranal dually inhibits the IMD pathway and activates the antioxidant Nrf2 pathway [30]. Additionally, Danggui Buxue Decoction and its active components (resveratrol, magnolol) co-suppress Toll and IMD pathways to relieve intestinal inflammation [147]. Xuanfei Baidu Decoction (XFBD, detailed herbal composition of this formulation is provided in the Table S7), consisting of 13 herbal materials, ameliorates DSS-induced intestinal injury, preserves barrier integrity, reduces ROS, remodels microbiota, and inhibits IMD, Toll, JNK and JAK/STAT pathways to prolong fly lifespan [148].

(2) Nrf2/Keap1 antioxidant pathway. Common IBD inducers (DSS, SDS, Ara-C) suppress Nrf2-Keap1 pathway activity, causing excessive ROS accumulation, aggravated lipid peroxidation and intestinal oxidative damage [143]. Multiple natural products, including *Flos Puerariae*, *Astragalus membranaceus* extracts, bilberry anthocyanins and *Hylotelephium erythrostictum* extracts, alleviate DSS-induced or spontaneous intestinal inflammation by activating Nrf2-Keap1 signaling and eliminating intracellular ROS [140,145,149]. Notably, Nrf2 activation is a common protective endpoint of natural product intervention, yet it remains unclear whether such activation results from direct pathway agonism or indirect ROS scavenging in *Drosophila* IBD models.

(3) JNK/JAK/STAT pathway. The JAK/STAT pathway is a central regulator of ISC proliferation; Upd2/Upd3 (mammalian IL-6 homologs) drive aberrant ISC overgrowth via JAK/STAT activation [141]. Excessive ROS triggers JNK/MAPK overactivation, forming a self-amplifying “oxidative stress-JNK-inflammation” vicious cycle that worsens intestinal damage [150]. Activated JNK signaling stimulates intestinal epithelial cells to secrete Upd2 and Upd3, the *Drosophila* homologs of mammalian IL-6. These cytokines further trigger the JAK/STAT pathway in intestinal stem cells (ISCs) and promote ISC proliferation during intestinal inflammatory stress. *Flos Puerariae* extract concurrently regulates Nrf2/Keap1, JAK/STAT and Wnt pathways to inhibit ISC hyperproliferation [141]. Total ginsenosides suppress MAPK overactivation by downregulating p-JNK/p-ERK, relieving SDS-induced intestinal inflammation [151]. *Astragalus membranaceus* and its active compounds (formononetin, isoliquiritigenin, astragalosides, caffeic acid, etc.) synergistically inhibit JNK and JAK-STAT signaling [148]. *Astragalus membranaceus* extract and its bioactive compounds, as well as XFBD inhibit both JNK and JAK/STAT [148,149].

(4) Intestinal protection and barrier function. Intestinal barrier disruption is the hallmark of *Drosophila* IBD, attributed to disordered ISC differentiation, excessive epithelial apoptosis and chronic inflammation. The Slit/Robo pathway governs ISC differentiation into enteroendocrine cells (EE). *Atg16* or *Rab19* deficiency reduces Slit secretion, blocks EE cell maturation, disrupts intestinal cell homeostasis and triggers chronic inflammation [144]. Excessive epithelial apoptosis further destroys barrier integrity. The chemotherapeutic agent Ara-C upregulates pro-apoptotic genes (*reaper*, *dcp-1*, *drice*) to induce massive epithelial apoptosis and barrier leakage [145]. Inflammatory factors and ROS also promote epithelial apoptosis via mitochondrial and death receptor pathways, and the imbalance of epithelial proliferation and apoptosis ultimately collapses intestinal mucosal structure and function [150]. Multiple natural products protect intestinal cells. Quercetin reduces ROS, restores Atg16 expression and Slit secretion, and stabilizes ISC lineage differentiation to relieve inflammation [152]. Caffeic acid and silibinin inhibit JNK-mediated inflammation, oxidative stress and epithelial apoptosis [153,154]. Curcumin and thymoquinone downregulate pro-apoptotic genes to mitigate chemotherapy-induced intestinal barrier damage [155,156]. These compounds target both *slit/robo*-mediated cell differentiation and apoptosis-proliferation balance, showing great intestinal protective potential.

(5) Intestinal flora. *Drosophila* has a simple, well-defined gut microbiota dominated by *Acetobacter* and *Lactobacillus* [32,157]. IBD-model flies exhibit reduced microbial α-diversity, depletion of core beneficial strains (*Lactobacillus plantarum*, *Acetobacter pomorum*), and overproliferation of harmful Proteobacteria and opportunistic pathogens [30,158]. Such microbial dysbiosis impairs intestinal immune barrier and ISC homeostasis, further amplifying midgut inflammatory cascades. Herbal medicines and their active metabolites effectively reverse IBD-associated microbial disorders. Polysaccharides from *Acanthopanax senticosus* and *Astragalus membranaceus* exert prebiotic effects by restoring beneficial *Lactobacillus* and *Acetobacter* abundance and suppressing pathogenic *Serratia* and *Pseudomonas* colonization [159,160,161]. Flavonoids and phenolic acids including quercetin and curcumin restructure gut microbial composition, reduce harmful bacteria load and improve microbial community evenness in inflamed flies [162,163]. The regulatory mechanisms of natural products against *Drosophila* IBD are summarized in Table S8.

#### 2.3.3. Advantages and Limitations of *Drosophila* IBD Models

The fly midgut has a simple, well-defined epithelial architecture with specific cell lineage markers, supporting efficient observation of intestinal stem cell homeostasis without complicated pathological slicing. However, *Drosophila* lacks adaptive immune cells such as T cells, so the screening results cannot reflect the regulatory effect of natural products on human adaptive intestinal immunity, which creates a translational gap.

### 2.4. Tumor Models

#### 2.4.1. Common Tumor Modeling Methods in *Drosophila*

From the perspective of tumor pathogenesis, malignant tumors are typical gene-driven diseases, primarily caused by acquired functional mutations that activate oncogenes and loss-of-function mutations that inactivate tumor suppressor genes [164]. Accordingly, *Drosophila* tumor models are constructed based on two core genetic alterations: oncogene overactivation and tumor suppressor gene inactivation [165], utilizing three classic genetic tools for targeted genomic modification [166].

The Gal4/UAS binary system serves as the fundamental genetic tool for *Drosophila* tumorigenesis. Through tissue-specific strain crossing, it mediates spatiotemporal overexpression of proto-oncogenes (*ras*^V12^, *egfr*, *pi3k*, *ret*, *braf*, etc.) and transcriptional silencing of endogenous tumor suppressors (p53, Pten, Apc, lgl, scrib) [167,168]. Featuring high tissue specificity and simple operation, this system is mainly used for benign tumor construction and preliminary signaling pathway verification. However, its non-selective genomic modification in target tissues cannot recapitulate the monoclonal tumor niche and tumor-stroma interactive microenvironment of human malignant tumors [169], limiting its application in research on malignant tumor progression and cell competition [170].

The FLP/FRT-MARCM mosaic clonal system is the gold-standard platform for constructing invasive malignant *Drosophila* tumors and is widely applied in oncology and developmental biology research [171,172]. This recombination-dependent system generates single-cell-derived mutant clones in wild-type epithelial tissues, accurately recapitulating the dual-hit oncogenic mechanism involving Ras proto-oncogene activation and biallelic deletion of the *scrib/lgl* polarity tumor suppressor genes [173,174]. Fluorescently labeled mutant clones simulate the monoclonal origin and stromal encapsulation of human primary tumors, along with spontaneous EMT activation and distant organ metastasis [170,175]. Compared with the ubiquitous modification of the Gal4/UAS model, MARCM chimeric tumors exhibit higher pathological consistency with human solid tumors, making them the core model for studying malignant tumor initiation, metastasis and tumor-stroma crosstalk [165].

The optimized CRISPR-Cas9 genome-editing system enables high-precision *Drosophila* tumor modeling and overcomes the inherent defects of traditional hybridization-based genetic modification [23,176]. It supports precise knockout of endogenous tumor suppressor genes (*apc, scrib*, etc.) and site-specific knock-in of clinically hotspot mutant proto-oncogenes such as human KRAS and BRAF [165]. This system eliminates the genetic background interference caused by traditional balancer chromosome strains and accelerates the construction of multi-mutant and humanized tumor models [177]. These gene-edited models closely match the somatic mutation characteristics of clinical cancers, reduce false-positive results in anti-tumor drug screening, and facilitate precision oncology research and high-throughput screening of driver genes [178]. Detailed modeling protocols and evaluation indexes of *Drosophila* tumor models are summarized in Table S9.

Beyond transgenic and dietary models, genotoxic compounds have also been used to investigate genotoxic and oncogenic responses as well as DNA damage repair in *Drosophila melanogaster*. Streptonigrin, a reactive oxygen species inducer, and 4-nitroquinoline-N-oxide (4NQO), a potent mutagen, are widely employed in the somatic mutation and recombination test (SMART) to evaluate genotoxic and antigenotoxic activities [179,180,181]. These compounds induce somatic mutations and chromosomal rearrangements in fly tissues, allowing for the rapid assessment of the genotoxic potential of environmental agents and the antigenotoxic effects of natural products.

#### 2.4.2. Research Progress and Mechanism of Natural Products in Tumor Treatment

**Figure 5:**
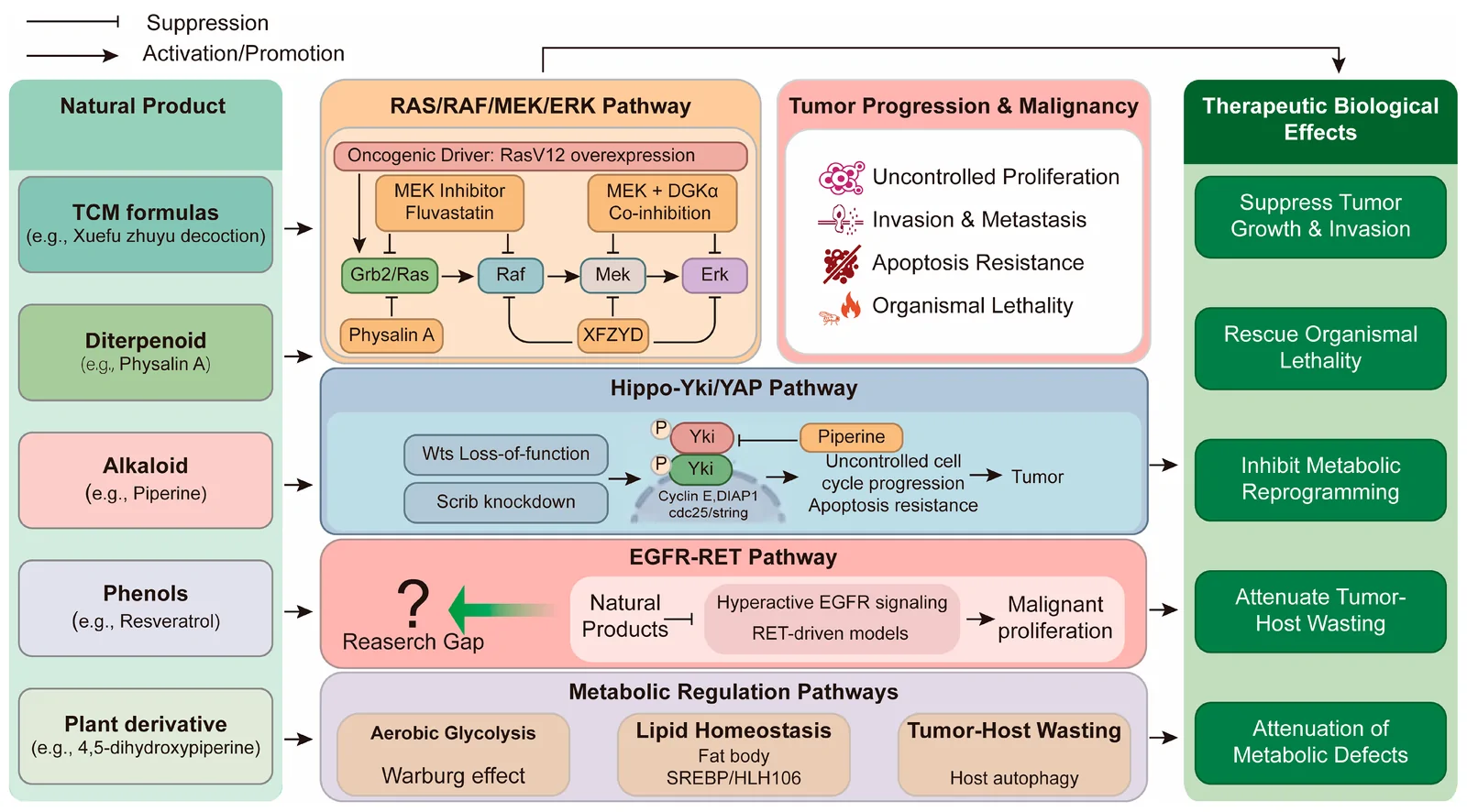
Multi-pathway molecular targets and anti-tumor effects of natural products in *Drosophila* tumor models. Herbal extracts and phytochemicals exert anti-malignant activities by repressing RAS/RAF/MEK/ERK, restoring disrupted Hippo/Yki signaling, targeting EGFR/RET kinase cascades, and correcting tumor metabolic defects including glycolysis dysregulation and cancer cachexia. The comprehensive therapeutic benefits of natural interventions are summarized on the right panel.

*Drosophila* tumor pathogenesis involves oncogene activation, tumor suppressor inactivation, metabolic reprogramming, and tumor-host crosstalk. Existing studies demonstrate a consistent mechanistic pattern. Different NPs act through distinct upstream pathways. However, they ultimately converge on a small set of conserved signaling hubs. These hubs include RAS/MAPK, Hippo/YAP, EGFR/RET, and metabolic stress response pathways (Figure 5).

(1) RAS/RAF/MEK/ERK pathway. Oncogenic Ras^V12^ overexpression is the primary driver activating the RAS/RAF/MEK/ERK cascade in *Drosophila* tumor models, and *Drosophila*-based drug screens support dual-targeting strategies for Ras-driven malignancies. The combination of MEK inhibitor trametinib and fluvastatin exerts synergistic anti-tumor effects and rescues lethality in Ras/PTEN-deficient *Drosophila* lung tumors, with consistent efficacy in human A549 cells [168]. Co-inhibition of MEK and diacylglycerol kinase alpha (DGKα) also produces robust synergism by elevating diacylglycerol levels and epigenetically inducing p38-mediated tumor quiescence, validating the rationale for dual targeting of the RAS-MAPK and DGKα pathways [182,183]. Additionally, vitamin B3 inhibits the progression of *ras*^V12^/*scrib*^−^/^−^ invasive tumors via regulating redox balance, mitochondrial function and autophagy [184].

Numerous natural products and herbal medicines suppress Ras-driven tumorigenesis by blocking hyperactivated RAS/MAPK signaling. Xuefu Zhuyu Decoction reduces Ras activity and the phosphorylation of Raf, Mek and Erk, thereby inhibiting overgrowth and invasion in *ras*^V12^/*lgl*^−^/^−^ *Drosophila* malignant tumors [178]. Physalin A, a diterpenoid from *Physalis angulata* L., blocks the upstream Grb2/RAS node of MAPK signaling, suppresses PI3K/Akt, MMP/uPA and JNK/p38/AP1 pathways, and dose-dependently inhibits tumor formation and distant metastasis in Ras^V12^/scrib^−^/^−^ *Drosophila* models [185]. Moreover, the optimized 4H-chromene derivative 5f acts as a dual Raf1/JNK1 inhibitor via competitive binds to the ATP pockets of both kinases. Dual blockade of RAS-Raf1 and JNK/MAPK signaling confers superior anti-metastatic efficacy to sorafenib in *scrib*-deficient tumors with favorable druggability [186].

(2) Hippo/Yki/YAP pathway. The Hippo pathway is an evolutionarily conserved regulator of tissue growth and apoptosis. The pathway derives its name from the Hippo (Hpo) kinase—mutations in the *hpo* gene result in excessive tissue overgrowth, producing a “hippopotamus”-like phenotype [187,188]. In *Drosophila*, the core tumor suppressor kinase Warts (Wts) phosphorylates the transcriptional co-activator Yorkie (Yki, mammalian YAP orthologue), retaining Yki in the cytoplasm and inhibiting the transcription of pro-proliferative and anti-apoptotic genes [189]. Loss-of-function *wts* mutations abolish this inhibitory phosphorylation, triggering excessive nuclear translocation of Yki. Activated Yki upregulates *cyclin* E, *cdc*25/string and *diap*1, driving uncontrolled cell cycle progression and apoptosis resistance to initiate epithelial tumor formation.

Disrupted epithelial polarity is a key cooperative event triggering Hippo pathway inactivation and tumor progression. Knockdown of the polarity determinant Scrib disrupts epithelial structure and promotes tumor invasion through two parallel mechanisms: activating the JNK/MMP1 axis to enhance cell motility and extracellular matrix degradation, and suppressing Hippo signaling to potentiate Yki-driven proliferation [88]. Notably, Scrib deficiency cooperates with oncogenic RAS signaling, making cell polarity a critical node bridging RAS/MAPK and Hippo pathways.

Piperine and its derivative 4,5-dihydroxypiperine suppress clonal tumor expansion in *wts*-mutant *Drosophila* epithelia by antagonizing aberrant Yki activity, which reduces the expression of anti-apoptotic DIAP1 and cell cycle regulator stg (string). These findings confirm that natural products can target dysregulated Hippo-Yki signaling *in vivo* and validate Yki as a druggable target for phytochemical anticancer therapy [190].

(3) EGFR-RET pathway. Oncogenic activation of EGFR and RET tyrosine kinases drives malignant proliferation and developmental disorders in *Drosophila* tumor models. Vandetanib, a multi-target tyrosine kinase inhibitor (TKI) against RET, VEGFR and EGFR, dose-dependently rescues MEN2B-related developmental defects and organismal lethality in *Drosophila* models [176]. *In vivo* compound screening based on EGFR-driven *Drosophila* lung tumors efficiently identifies synergistic agents for clinical TKI combination therapy. A low-dose afatinib screening platform targeting hyperactive EGFR signaling successfully identified bazedoxifene, which alleviates EGFR-induced lethality via selective inhibition of the JAK/STAT cascade [168]. Overall, *Drosophila in vivo* screening systems enable the discovery of both single-target EGFR inhibitors and adjuvants that enhance first-line TKI efficacy.

Nevertheless, a notable research gap exists in natural products that concurrently target EGFR and RET. A 2024 study reported psoralen (from *Psoralea Fructus*) as an EGFR-targeting compound but lacked RET-related validation and *Drosophila* model verification [191]. Conversely, a 2012 study validated RET-driven *Drosophila* models for drug screening but only identified synthetic compounds [192]. Therefore, *Drosophila* models hold great potential for screening natural products that dually modulate EGFR and RET pathways, representing an underexplored direction for future herbal anti-tumor research.

(4) Metabolic regulation pathways. Oncogenic mutations induce extensive metabolic reprogramming to sustain unlimited tumor proliferation, including enhanced aerobic glycolysis (Warburg effect), disrupted lipid homeostasis, altered amino acid metabolism, and dysregulated TOR/Insulin-PI3K nutrient-sensing signaling [22,193].

In Ras/scrib-deficient *Drosophila* tumors, Myc activation and HIF-1α stabilization upregulate glycolytic enzymes and suppress oxidative phosphorylation, leading to prominent Warburg effect activation. Multiple plant-derived natural products, including resveratrol, EGCG and gossypol, target key glycolytic regulators (HK II, PKM2) in mammalian cancer models [194,195,196,197].

*Drosophila* tumor models also recapitulate tumor-induced systemic metabolic disorders, including lipid remodeling, abnormal iron metabolism and host cachexia. Tumor-derived signals remotely regulate fat body lipid metabolism via JAK/STAT and insulin signaling, with SREBP/HLH106 serving as the core regulatory hub [198]. Tumor cachexia in *Drosophila* is driven by abnormal extracellular matrix deposition in the fat body. This deposition is mediated by insulin and TGF-βsignaling. Meanwhile, host autophagy mediates systemic nutrient mobilization to support tumor growth. This acts as a critical non-cell-autonomous pro-tumor mechanism [199,200].

Given the high conservation of metabolic pathways and convenient genetic manipulation, *Drosophila* serves as a powerful *in vivo* platform to screen natural products that block tumor metabolic reprogramming, inhibit tumor growth and relieve cancer cachexia. It enables effective validation of compound metabolic targets via combined gene knockdown and pharmacological intervention, facilitating the development of novel metabolism-targeted anticancer drugs. However, high-quality relevant studies remain insufficient. The mechanisms of natural products against tumors are summarized in Table S10.

Beyond direct modulation of signaling pathways, phenolic compounds have emerged as epigenetic modulators in oncogenesis. Polyphenols such as curcumin, resveratrol, and epigallocatechin gallate (EGCG) can influence DNA methylation patterns, histone acetylation, and non-coding RNA expression, thereby reprogramming the cancer epigenome [201]. Singaravelan and Tollefsbol systematically summarized the core epigenetic regulatory axes of dietary polyphenols against malignancies, and Altomare (2025) further supplemented *in vivo* evidence verifying these epigenetic effects in nutrient intervention models [201,202]. While these epigenetic actions are increasingly recognized, their validation in *Drosophila* models remains an emerging area of investigation.

#### 2.4.3. Advantages and Limitations of *Drosophila* Tumor Models

*Drosophila* tumor models are easy to construct, and fluorescent genetic labeling tracks mutant clones and transplanted tumor cells conveniently. However, current *Drosophila* anti-tumor screening heavily relies on widespread GAL4 overexpression systems rather than metastatic MARCM mosaic clones, yielding limited data on compound anti-invasion capacity. A notable research gap lies in natural candidates concurrently targeting EGFR and RET kinases, and the absence of adaptive immune cells including T cells in flies disables assessments of immunomodulatory and immune checkpoint-targeted agents. The epigenetic regulation studies using natural products in the fruit fly model need to be strengthened.

### 2.5. Neurodegenerative Disease Models

Neurodegenerative diseases, including Alzheimer’s disease (AD) and Parkinson’s disease (PD), are characterized by progressive neuronal loss and functional decline. Their complex pathogenesis and multifactorial molecular mechanisms limit the utility of conventional cell models, while mammalian models are costly, time-consuming, and unsuitable for high-throughput screening of natural products. With high nervous system conservation, mature genetic tools and quantifiable phenotypes, *Drosophila* has become a key *in vivo* system for clarifying AD/PD mechanisms, identifying therapeutic targets and assessing neuroprotective effects of natural products. Various *Drosophila* AD and PD models have been established, including pathogenic protein transgenic overexpression, disease-related gene mutation and neurotoxin-induced models. These models recapitulate core pathologies such as abnormal protein aggregation, mitochondrial dysfunction, oxidative stress and neuroinflammation, providing a reliable platform for studying multi-target and multi-pathway neuroprotective mechanisms of natural products.

#### 2.5.1. Common AD/PD Modeling Methods in *Drosophila*

The *Drosophila* brain contains ~100,000 neurons and highly conserved core neurotransmitter systems (dopamine, serotonin, acetylcholine) homologous to humans, enabling recapitulation of key pathological phenotypes such as locomotor dysfunction, shortened lifespan, neuronal loss, and amyloid deposition, supporting mechanistic research and drug screening [20,31]. Below we summarize AD (A*β*/tau-based) and PD (genetic/neurotoxin-induced) models.

*Drosophila* AD models center on A*β* amyloidosis and tau hyperphosphorylation. The A*β* toxicity model is the most common: human A*β*1-42/A*β*1-40 expressed via GAL4/UAS causes age-dependent amyloid deposition, locomotor deficits, shortened lifespan, and rough-eye phenotype. Arctic (E22G) or tandem A*β*42 constructs enhance proteotoxicity and drug sensitivity [203]. Recently, the development of intestine-specific A*β*42 expression models has expanded new avenues for studying gut–brain axis crosstalk and peripheral proteotoxicity in AD [204]. Lacking endogenous tau, *Drosophila* enables accurate modeling of tau hyperphosphorylation, microtubule instability, and axonal transport defects [205,206]. A*β*42/tau double models recapitulate both pathologies and are suitable for multi-target drug screening [207].

*Drosophila* PD models include genetic and neurotoxin-induced types, focusing on α-synuclein aggregation, mitophagy disorders, and oxidative stress. α-synuclein overexpression in dopaminergic neurons replicates Lewy body-like inclusions and progressive neuronal loss; A30P/G51D mutations accelerate phenotypes [208,209]. Mutants of *lrrk2*, *pink1*, *parkin*, *dj-1* recapitulate kinase dysregulation, mitophagy defects, and oxidative stress, aiding pathway dissection [210,211,212,213]. Among them, PINK1^B9^ mutants are ideal for screening mitochondria-targeted natural products. Plant compounds reportedly modulate PD-related kinases (LRRK2, PINK1, GSK-3*β*, CDK5); e.g., ginseng total protein protects PINK1^B9^ flies by improving mitochondrial function [214,215]. Rotenone and paraquat induce stable PD models via mitochondrial complex I inhibition or dopaminergic neuron damage, supporting rapid preliminary screening of neuroprotective natural products [216,217,218]. Detailed modeling and assessment indices are listed in Tables S11 and S12.

#### 2.5.2. Research Progress and Mechanism of NPs in AD/PD Treatment

**Figure 6:**
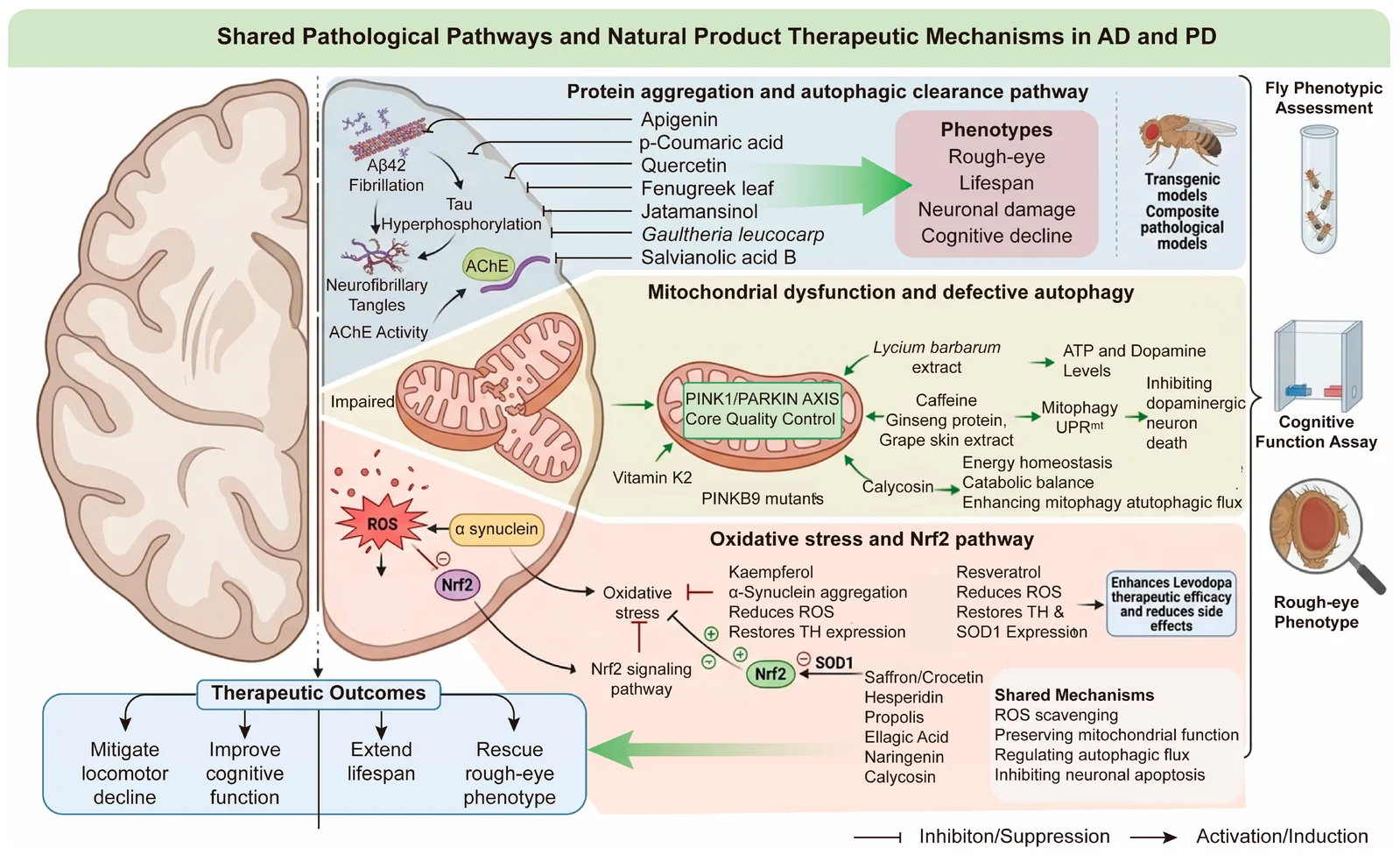
Pathological signaling cascades of AD/PD and multi-target neuroprotective mechanisms of natural products in *Drosophila* neurodegenerative models. Natural phytochemicals and herbal extracts alleviate neurodegeneration via suppressing toxic protein aggregation, restoring PINK1/PARKIN mitophagy, and activating Nrf2 antioxidant pathway. *Drosophila* phenotypic assessment systems and core therapeutic outcomes of natural interventions are summarized on the right and bottom panels.

Despite distinct etiological triggers and affected brain regions, AD and PD exhibit highly conserved pathological pathways in *Drosophila*, mainly including protein misfolding and aggregation, mitochondrial dysfunction, oxidative stress imbalance, and neuroinflammation (Figure 6).

(1) Protein aggregation and autophagic clearance pathway. A*β*42 fibrillation and tau hyperphosphorylation jointly drive neuronal damage and cognitive decline. Apigenin dose-dependently suppresses A*β*42 aggregation, reduces acetylcholinesterase (AChE) activity, relieves oxidative stress, and markedly mitigates locomotor decline in AD flies [137]. p-Coumaric acid blocks A*β*42 fibrillation *in vitro* and mitigates toxicity while extending lifespan in *Drosophila* A*β*42 models [219]. Ethyl caffeate ameliorates rough-eye phenotype, prolongs lifespan, and attenuates A*β*42-induced PC12 cell death [220]. Fenugreek leaf extract enhances locomotor and cognitive function by inhibiting AChE, monoamine oxidase, and apoptotic pathways, as well as regulating metabolism [221]. Quercetin relieves A*β* toxicity via regulating Cyclin B expression [222]. Methylene blue reduces tau phosphorylation by inhibiting MARK4/PAR1 [223], and jatamansinol protects against both A*β*- and tau-mediated neurotoxicity, suitable for composite pathological models [224]. *Gaultheria leucocarpa* extract inhibits A*β* fibrillization, promotes mitophagy via AMPK/ULK1, and suppresses PI3K/AKT/mTOR [225]. Salvianolic acid B alleviates A*β*42 toxicity in *Drosophila*, with transcriptomic changes in multiple stress-response pathways [226]. Notably, dietary polyphenols including gallic acid, 3-hydroxytyrosol, and quercetin have been shown to modulate cholinesterase activity in wild-type *Drosophila*, with distinct structure-dependent inhibitory profiles and hormetic dose responses observed *in vivo* [227].

(2) Mitochondrial dysfunction and defective autophagy. Mitochondrial damage and insufficient autophagy contribute critically to AD/PD pathogenesis, with the PINK1/Parkin pathway as a core mitochondrial quality-control regulator. Vitamin K2 attenuates A*β*42 neurotoxicity by activating autophagy (upregulating Atg8a and Atg6a) and improving mitochondrial respiratory function [228]. *Lycium barbarum* extract repairs mitochondrial injury, elevates ATP and dopamine, and rescues locomotor and muscular atrophy in Pink1^B9^ mutant flies [229]. Ginseng protein activates the mitochondrial unfolded protein response (UPRmt), restores ATP and dopamine levels, and improves behavioral abnormalities in Pink1^B9^ mutants [230]. Caffeine, ginseng protein, and grape skin extract promote neuronal survival by enhancing mitochondrial complex activity, inducing UPRmt, and facilitating mitophagy [231,232,233]. In paraquat-induced Parkinson’s disease (PD) oxidative injury, calycosin inhibits dopaminergic neuron death by enhancing mitophagy and autophagic flux, suppressing mTOR/S6K/4EBP1 signaling, and restoring energy and catabolic homeostasis [234]. Furthermore, combined intervention with polyphenol supplementation and moderate exercise has been demonstrated to synergistically alleviate Aβ-induced neurotoxicity and locomotor impairment in transgenic AD *Drosophila*, although excessive polyphenol doses can paradoxically exacerbate neurodegeneration [235].

(3) Oxidative stress. Oxidative stress is an early common pathological feature of AD and PD. Kaempferol binds α-synuclein, inhibits its aggregation, reduces oxidative stress, restores tyrosine hydroxylase (TH) expression, and improves locomotor deficits in PD flies [146]. Resveratrol lowers oxidative stress products, restores TH and SOD1 expression in parkin mutants, and enhances levodopa efficacy while reducing side effects [236]. Saffron/crocetin, hesperidin, propolis, ellagic acid, naringenin, and calycosin exert broad neuroprotection in transgenic and toxin-induced models, mainly via ROS scavenging, maintaining mitochondrial function, regulating autophagic flux, and inhibiting neuronal apoptosis [218,234,237,238,239,240]. The mechanisms of NPs against AD/PD are summarized in Tables S13 and S14. Carotenoids, have been investigated primarily in PD models, where β-carotene and astaxanthin show neuroprotective effects against rotenone- and MPTP-induced oxidative damage [241,242,243]. However, their application to other *Drosophila* disease models such as nephrolithiasis, IBD, and cancer remains largely unexplored.

Notably, accumulating mammalian evidence indicates that several of these natural compounds may also act at the epigenetic level to modulate dopaminergic pathway activity and neuronal resilience [244,245]. While environmental stressors are confirmed to reshape the epigenetic landscape of *Drosophila* dopaminergic neurons [246], investigations exploring whether natural phytochemicals reverse stress-induced epigenetic dysregulation in fly neurodegeneration models remain limited, representing a promising but still under-explored regulatory layer for future mechanistic validation.

#### 2.5.3. Advantages and Limitations of Neurodegenerative Disease Models

Multiple stable AD/PD fly lines recapitulate diverse disease triggers; rough eye phenotype serves as a straightforward readout of neuronal degeneration. Nevertheless, most studies rely on single transgenic models (Aβ or α-synuclein) without complex pathological models mimicking mixed lesions in clinical AD/PD. Few have investigated gut–brain axis regulation by oral natural products in flies. Future work should use dual-transgenic flies and tissue-specific gene silencing to validate direct neuroprotective targets of candidate natural products.

## 3. Shared Pharmacological Characteristics of Natural Products Across *Drosophila* Disease Models

**Figure 7:**
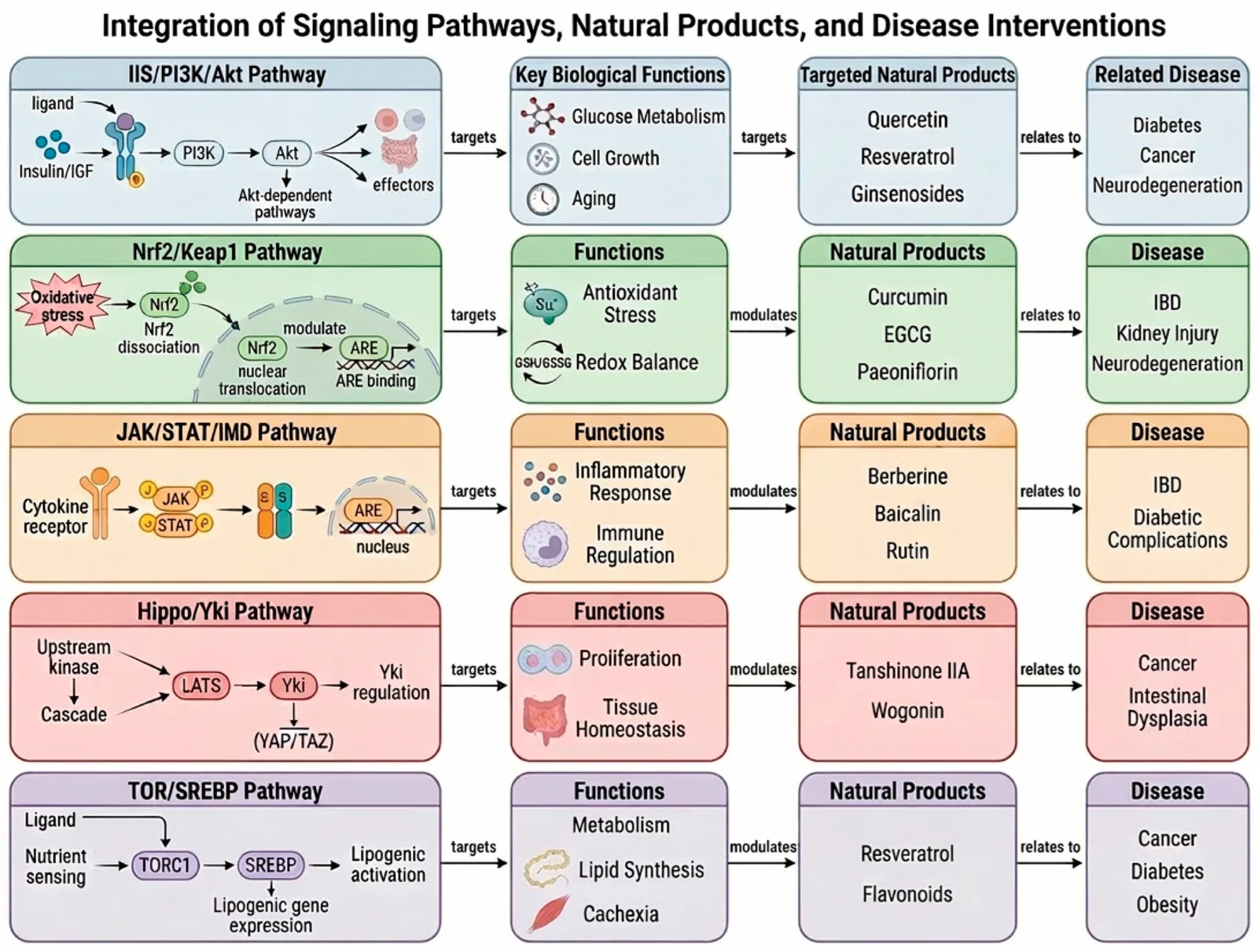
Conserved signaling pathways targeted by natural products across disease models. Related natural compounds are detailed in Table S15.

Across the six *Drosophila* disease models, this review establishes a unified mechanistic framework for the cross-disease bioactivities of NPs, highlighting five evolutionarily conserved signaling axes as core therapeutic targets: IIS/PI3K/Akt/FOXO, JNK/JAK/STAT, Nrf2/Keap1, mTOR/TORC1, and IMD/Toll immune signaling (Figure 7). These pathways were selected for their deep *Drosophila*-human conservation to ensure translational value and extensive experimental support across multiple diseases; nearly all bioactive NPs against metabolic, inflammatory, cancer, and neurodegenerative disorders modulate at least one of them. Beyond modulating specific signaling cascades, alleviation of oxidative stress—via direct ROS scavenging or activation of Nrf2/Keap1—is a universal mechanism of many natural products. Notably, antioxidants often exhibit hormetic dose–response effects in *Drosophila*: low doses trigger adaptive protection while high doses become pro-oxidant [247]. Although systematic studies of co-exposure to natural and synthetic antioxidants in disease-specific fly models remain limited, the fly’s genetic tractability makes it an ideal platform for future investigation of such combinatorial effects—a direction highly relevant to human exposure scenarios [248,249]. The natural products and herbal medicines exhibit two consistent, *in vivo* validated characteristics.

First, they simultaneously modulate multiple signaling nodes. For example, caffeic acid inhibits intestinal α-amylase, activates Nrf2, blocks JNK-mediated inflammation and apoptosis, and alleviates metabolic and intestinal barrier damage. Quercetin regulates Slit/Robo to normalize intestinal stem cell differentiation, reduces neuronal ROS, and ameliorates hyperglycemia-induced lipid accumulation. Resveratrol targets AMPK-TOR to rebalance glycolipid metabolism, suppresses tumor Warburg glycolysis, and relieves oxidative neuronal injury across metabolic, cancer, and neurodegenerative models. It is worth noting that these cascades act as universal stress-response modules, and their activation in fly models reflects shared cellular stress responses rather than uniform NP actions. Different herbal compounds may target distinct nodes of the same pathway or trigger unique crosstalk, resulting in disease-specific effects. Oxidative stress represents a highly conserved and widespread trigger of such stress responses across species and cell types. This accounts for why regulating oxidative stress commonly contributes to the broad yet context-dependent bioactivities of natural products.

Second, diverse disease phenotypes can be improved by targeting the same evolutionarily conserved signaling pathways, including IIS/PI3K/Akt, Nrf2/Keap1, JNK/JAK/STAT, and mTOR. Dysregulation of these cascades leads to shared pathological features—ROS accumulation, chronic inflammation, mitochondrial dysfunction, and disrupted proliferation/apoptosis—regardless of tissue or lesion type. Compounds such as curcumin, berberine, and astragalus polysaccharides restore homeostasis in these core pathways, attenuating renal injury, intestinal inflammation, neuronal loss, and tumor overgrowth in corresponding fly models.

These two features enable *Drosophila* multi-disease models to support rapid phenotypic screening and mechanistic dissection of herbal ingredients. Combined with genetic tools, these platforms efficiently identify multiple molecular targets per compound and characterize target synergies. Linking herbal compounds to specific targets allows rational prediction of expanded therapeutic indications and *in vivo* validation of cross-disease efficacy.

## 4. Common Deficiencies of Current *Drosophila*-Based Natural Product Research

By summarizing current research on natural product pharmacology in *Drosophila* disease models, we identified common limitations in this field.

First, metabolic differences between flies and mammals prevent quantitative translation of pharmacokinetic data. Flies use trehalose as the main circulating sugar, have limited overlap in cytochrome P450 orthologs with humans (Table S16) [250,251,252,253,254,255,256,257], and dietary administration prevents accurate measurement of dose–response relationships and half-lives. Moreover, *Drosophila* lacks a closed circulation and a vertebrate-like blood–brain barrier. Its hemolymph–brain barrier is glia-derived rather than endothelial (Table S17) [258,259,260,261]. This results in distinct CNS penetrance and neuroprotective responses compared with mammals. Together, these interspecies differences explain why many compounds showing promise in flies frequently fail to reproduce activity in higher models. To mitigate such translational bias, recent advances have established humanized *Drosophila* lines expressing key human metabolic enzymes, enabling more faithful prediction of *in vivo* drug metabolism and efficacy prior to mammalian testing.

Anatomical simplifications—such as Malpighian tubules lacking glomeruli, a tubular dorsal vessel, and an unstructured midgut—mean processes involving glomerular filtration, ventricular remodeling, or mucus-dependent barrier function cannot be faithfully modeled. Immunologically, flies only possess innate Toll/IMD pathways without adaptive immune cells or microglia, limiting studies of adaptive immunity, immune-checkpoint cancer therapy, and neurodegenerative neuroinflammation. Genetically, the smaller genome and short lifespan cannot recapitulate polygenic interactions or chronic progressive human diseases, while transgenic overexpression often fails to mimic physiological, age-dependent expression patterns. A simple gut microbiota and limited behaviors also restrict microbiome and cognitive studies.

*Drosophila*-based natural product screening is plagued by critical confounders. ad libitum feeding causes compound degradation, variable intake and uneven exposure, while microinjection is unsuitable for large cohorts. Gut flora and sex differences introduce metabolic and bioactive variation. Fast larval growth limits synchronization, favoring adult flies unless development is studied. Genetic backgrounds alter pathway sensitivity. Solvents and crude extracts may cause off-target or nonspecific toxicity, which must be distinguished from true therapeutic effects.

Beyond the intrinsic and methodological limitations, research on natural products and herbal medicines carried out in fly models faces several widespread drawbacks that also persist in mammalian experiments. Most studies only evaluate crude mixed extracts without further separation and identification of core active monomers [173]. Mechanistic conclusions are simply drawn from changes in gene and protein expression, while genetic rescue tests with RNAi or mutant strains are rarely applied to confirm causal signaling links [249]. Additionally, there is a lack of research into synergistic interactions between different bioactive ingredients and their joint regulation of multiple pathways. Nevertheless, these research bottlenecks can be efficiently addressed relying on *Drosophila*’s accessible and well-established genetic tools. Accordingly, we propose a generalizable stepwise pipeline to address these current limitations.

## 5. Future Research Strategies

Despite the inherent and methodological limitations of *Drosophila*-based natural product research elaborated above, the unique strengths of *Drosophila* disease models render them an irreplaceable translational platform for natural product drug discovery and validation. To address the aforementioned research bottlenecks in including insufficient quantitative pharmacokinetic translation, incomplete pathological simulation, vague causal mechanism verification, and lack of in-depth exploration of compound synergistic effects, this section proposes a generalizable, optimized research framework for NPs.

(1) Screening, isolation of bioactive fractions and structural identification of active compounds. Crude herbal extracts with confirmed bioactivity in initial *Drosophila* screening were subjected to fractionation. Individual fractions were re-administered to the corresponding disease model flies to verify and confirm the active fractions. Those fractions with consistent pharmacological effects were further purified and analyzed by high-performance liquid chromatography-mass spectrometry (HPLC-MS) and nuclear magnetic resonance (NMR) spectroscopy. The chemical structures of the major bioactive constituents were then elucidated, and these individual compounds will be further validated using the *Drosophila* nephrolithiasis model.

(2) Signaling pathway analysis and multi-component synergy evaluation using *Drosophila* genetic tools. Key disease-related signaling pathway genes were regulated via classic *Drosophila* genetic methods (Figure 1). Changes in pathological phenotypes before and after compound treatment were compared to identify the target genes and signaling pathways modulated by active compounds. To evaluate synergistic effects, both single-fraction and multi-component combination treatments were applied, and phenotypic improvements were quantified. Candidate compounds with well-defined mechanisms were selected for further study. Additionally, mechanistic investigations should ideally integrate multiple regulatory layers—including transcriptional, post-translational, epigenetic, and gut microbiota-level changes—whenever experimental evidence permits, as these interconnected dimensions collectively shape the pharmacological outcomes of natural products *in vivo*.

(3) Stepwise translational validation from *Drosophila* to mammals. First, silico simulation was performed to predict the binding affinity between compounds and target proteins. In parallel with *in vivo* phenotypic screening, early programs like PASS and modern deep-learning platforms (e.g., DeepPurpose, ChemProp, graph neural networks) enable accurate, large-scale prediction of candidate targets and bioactivity profiles, effectively narrowing down compound libraries to complement Drosophila-based screening.

Before proceeding to mammalian validation, it is essential to evaluate the evolutionary conservation and homology of the targets and pathways identified in *Drosophila*. This assessment can be achieved through sequence alignment, pathway mapping, and functional validation in mammalian or human cell lines, supported by literature mining and molecular simulation methods. Prioritizing mechanisms and targets with high conservation increases the likelihood of successful translation and optimizes resource allocation in subsequent experiments. Second, the regulatory effects of compounds on human homologous signaling pathways were validated in human cell lines. Third, comprehensive preclinical assessments were conducted in mammal models, including pharmacokinetic profiling, long-term chronic toxicity testing, and adaptive immune response analysis. Multi-dimensional safety and efficacy evaluations were performed to identify molecules suitable for further preclinical development.

By using the established *Drosophila* nephrolithiasis model, our team successfully screened and identified core bioactive substances, including Kukoamine A, hydroxysafflower yellow A, salvianic acid A and calycosin-7-O-*β*-D-glucoside, which exert anti-urolithiasis effects through multiple distinct signaling pathways [111,114]. Mechanistic investigation of Kukoamine A further revealed that JAK/STAT inhibition suppresses DAO expression and oxalate synthesis, thereby identifying a novel therapeutic target for nephrolithiasis. The therapeutic efficacy of these active ingredients has been further verified in mouse models, strongly demonstrating the translational reliability and preclinical application potential of *Drosophila*-based screening systems. With the accumulation of completed cases, the established pipeline will be iterated to achieve higher standardization, and the evaluation system for translational potential will also be further refined.

**Figure 8:**
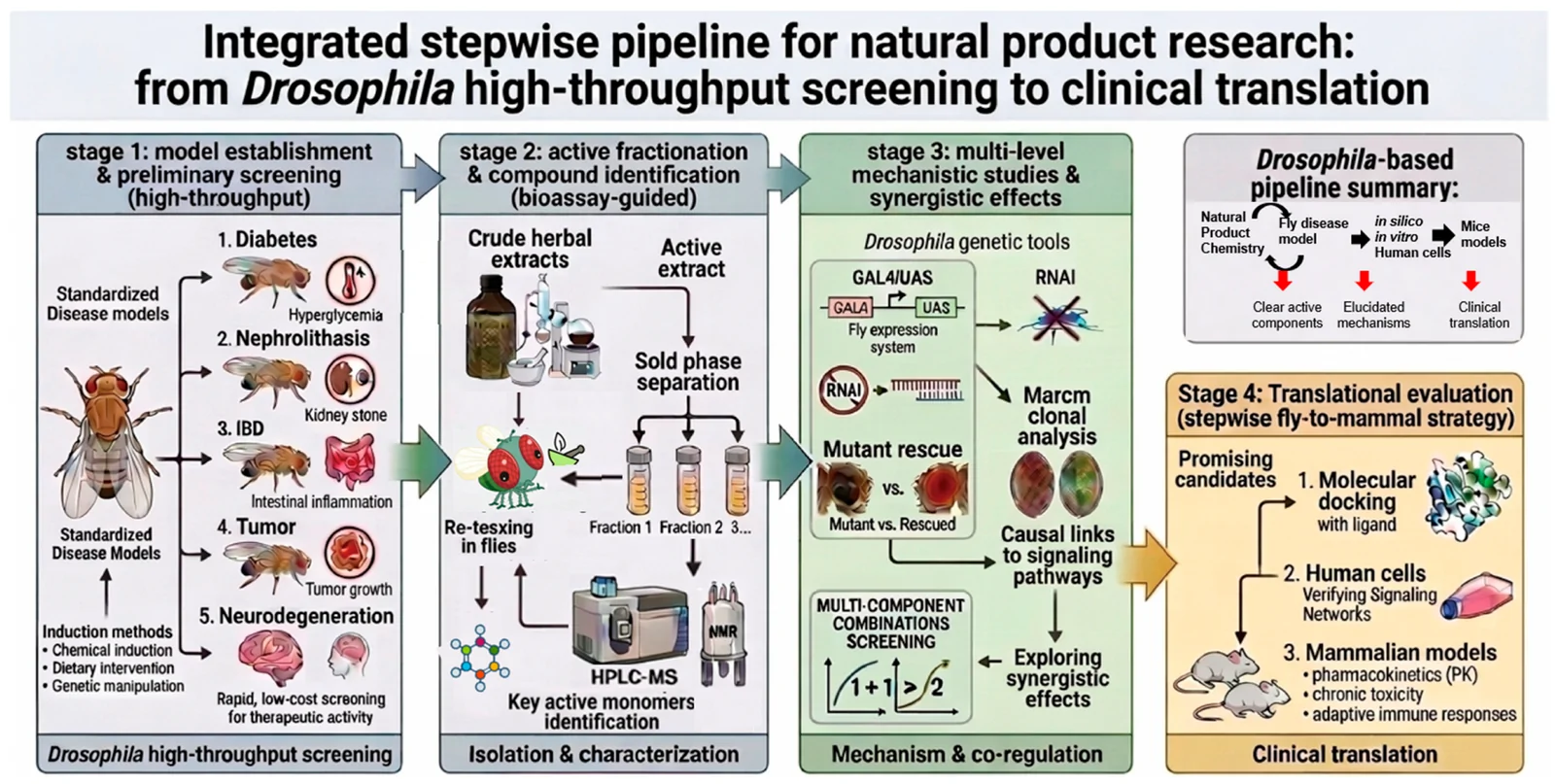
Stepwise integrated translational pipeline for natural product discovery, featuring a progressive *Drosophila*-to-mammal research strategy. The workflow includes four phases: high-throughput disease model screening in flies, bioassay-guided isolation of active monomers, genetic mechanistic dissection and synergistic combination evaluation, and layered mammalian translational verification. This pipeline overcomes major research bottlenecks by balancing screening efficiency and clinical translatability.

Overall, this pipeline leverages the high-throughput and genetic flexibility of *Drosophila* to overcome key bottlenecks in natural product research. The progressive fly-to-mammal strategy balances screening efficiency and clinical translatability (Figure 8).

Our fly-to-mammal pipeline has clear stepwise logic yet faces prominent cross-stage translational hurdles. Interspecies metabolic gaps lead to mismatched pharmacokinetics, low bioavailability and failed efficacy in mammals. Crude herbal mixtures suffer poor batch reproducibility due to variable raw materials and extraction methods. Early fly screening cannot rapidly confirm target engagement or chronic toxicity, delaying safety detection to expensive rodent tests. Moreover, natural product candidates meet strict clinical regulatory demands for purity, marker compounds and full safety records. These barriers disrupt linear translational workflows; future pipeline upgrades should integrate early PK prediction, standardized extracts and fly-based toxicity assays to reduce risks. These challenges underscore the need for a robust, multi-model system involving cell-based assays and mammalian models to ensure reliable translation. *Drosophila* alone cannot complete the entire drug development process, but it can effectively improve efficiency and reduce costs in early-stage discovery.

## 6. Conclusions

This review systematically summarizes recent advances in *Drosophila* models for natural product discovery against diabetes, nephrolithiasis, IBD, cancer, and neurodegenerative diseases. A key finding of this review is the identification of five evolutionarily conserved signaling pathways—IIS/PI3K/Akt/FOXO, JNK/JAK/STAT, Nrf2/Keap1, mTOR/TORC1, and IMD/Toll—as common molecular targets of natural products across diverse diseases. This convergence explains the multi-target and cross-disease efficacy of herbal medicines and provides a unified framework for understanding their pharmacological activities. Despite these advances, several challenges remain, including pharmacokinetic discrepancies, absence of adaptive immunity in flies, and the prevalence of studies using crude extracts over purified compounds. The stepwise translational pipeline proposed in this review—from fly screening to genetic dissection to mammalian validation—offers a practical strategy to address these bottlenecks, ultimately reducing the translational gap and accelerating natural product-based drug development.

## Data Availability

No new data were created or analyzed in this study. Data sharing is not applicable to this article.

## Supplementary Materials

The following supporting information can be downloaded at: https://www.mdpi.com/article/10.3390/biology15171447/s1, Table S1: Comparison of Carbohydrate and Lipid Metabolism Between Mammals (Human/Murine) and *Drosophila melanogaster*; Table S2: Construction methods, strains, and evaluation indicators of *Drosophila* diabetes models; Table S3: Targets, molecular mechanisms, and experimental results of representative natural products in *Drosophila* diabetes models; Table S4: Construction methods, strains, and evaluation indicators of *Drosophila* kidney stone models; Table S5: Targets, molecular mechanisms, and experimental results of representative NPs in *Drosophila* kidney stone models; Table S6: Construction methods, strains, and evaluation indicators of *Drosophila* IBD models; Table S7: Targets, molecular mechanisms, and experimental results of representative NPs in *Drosophila* IBD Models; Table S8: Herbal Composition of Traditional Chinese Medicine Compound Formulas in This Review; Table S9: Construction methods, strains, and evaluation indicators of *Drosophila* cancer models; Table S10: Targets, molecular mechanisms, and experimental results of representative NPs in *Drosophila* cancer models; Table S11: Construction methods, strains, and evaluation indicators of *Drosophila* AD models; Table S12: Construction methods, strains, and evaluation indicators of *Drosophila* PD models; Table S13: Targets, molecular mechanisms, and experimental results of representative NPs in *Drosophila* AD models; Table S14: Targets, molecular mechanisms, and experimental results of representative NPs in *Drosophila* PD models; Table S15: Comprehensive classification of natural products cited in this review: chemical classes, mechanisms of action, and disease associations; Table S16: Comparison of the Cytochrome P450 (CYP) Drug-Metabolism System Between *Drosophila melanogaster* and Humans; Table S17: Comparison of Blood–Brain Barrier (BBB) Structure and Permeability Between *Drosophila melanogaster* and Humans.

## Abbreviations

The following abbreviations are used in this manuscript:

A*β*	amyloid beta
ACC	acetyl CoA carboxylase
AChE	acetylcholinesterase
AD	Alzheimer disease
AMPK	adenosine monophosphate activated protein kinase
Ara C	cytarabine
CaOx	calcium oxalate
DGK α	diacylglycerol kinase alpha
DILPs	*Drosophila* insulin like peptides
DSS	dextran sulfate sodium
EE	enteroendocrine cell
EGFR	epidermal growth factor receptor
ERK	extracellular signal regulated kinase
FOXO	forkhead box O
HFD	high fat diet
HSD	high sugar diet
IBD	inflammatory bowel disease
IIS	insulin IGF 1 signaling
IMD	immune deficiency
ImpL2	Imaginal morphogenesis protein-Late 2
InR	insulin receptor
IPCs	insulin producing cells
ISC	intestinal stem cell
JAK/STAT	Janus kinase signal transducer and activator of transcription
JNK	c Jun N terminal kinase
Keap1	Kelch like ECH associated protein 1
MAPK	mitogen activated protein kinase
MEK	mitogen activated protein kinase kinase
MFS2	major facilitator superfamily 2
MMP1	matrix metalloproteinase 1
MPTP	1 methyl 4 phenyl 1,2,3,6 tetrahydropyridine
mTOR	mammalian target of rapamycin
NF κB	nuclear factor kappa B
Nrf2	nuclear factor erythroid 2 related factor 2
PD	Parkinson’s disease
PEPCK	phosphoenolpyruvate carboxykinase
PI3K	phosphatidylinositol 3 kinase
PINK1	PTEN induced kinase 1
PTEN	phosphatase and tensin homolog
Rab19	Ras related protein Rab 19
RNAi	RNA interference
ROS	reactive oxygen species
S1P	sphingosine 1 phosphate
Sip1	sodium hydrogen exchanger regulatory factor 1 homolog
Slit Robo	Slit roundabout
SOD	superoxide dismutase
Socs36E	suppressor of cytokine signaling 36E
SREBP	sterol regulatory element binding protein
SDS	sodium dodecyl sulfate
T2DM	type 2 diabetes mellitus
TCM	traditional Chinese medicine
TH	tyrosine hydroxylase
TKI	tyrosine kinase inhibitor
TORC1	target of rapamycin complex 1
TRF	time restricted feeding
Upd3	unpaired 3
YAP	Yes associated protein
Yki	Yorkie
