# Supplementary material for: Drosophila melanogaster Models for Natural Product Discovery: Cross-Disease Conserved Signaling Networks and a Generalizable Translational Pipeline
Source: Biology (Basel). 2026 Aug 24;15(17):1447. doi: 10.3390/biology15171447 (PMC13564642; doi:10.3390/biology15171447)
Supplement: Supplementary file 1 [file biology-15-01447-s001.zip › biology-4454386-supplementary.pdf]

Table S1. Comparison of Carbohydrate and Lipid Metabolism Between Mammals (Human/Murine) and *Drosophila melanogaster*

Feature	Mammals (Human / Murine)	*Drosophila melanogaster*	Mechanistic Notes	Refs
**Key metabolic organs**	Liver, white adipose tissue (WAT), skeletal muscle, pancreas	Fat body (combined liver + adipose functions), oenocytes (hepatocyte-like), gut	The fat body integrates hepatic and adipocyte functions in a single organ — a key anatomical difference — yet core metabolic programs are conserved.	[50-53]
**Major circulating carbohydrate**	Glucose (monosaccharide)	Trehalose (non-reducing disaccharide of glucose)	Different carrier sugars, but both are tightly regulated by IIS signaling; trehalose is synthesized from glucose and serves the same energetic role.	[54-56]
**Glycogen storage**	Liver, skeletal muscle	Fat body, body-wall muscle	Glycogen synthase (GS) and glycogen phosphorylase (GP) are conserved; hormonal regulation by insulin/glucagon (mammals) and DILPs/AKH (fly) follows the same logic.	[50]
**Lipid storage form**	Triacylglycerol (TAG) stored	Triacylglycerol (TAG) stored	Highly conserved from insects to mammals; LD-coating proteins	[56,57]

	in lipid droplets (LDs)	in lipid droplets (LDs)	(perilipins/Plin1 in mammals; Lsd-1/Lsd-2 in *Drosophila*) are functional orthologs.	
**Major circulating lipid**	Free fatty acids (FFA, albumin-bound); TAG transported in VLDL/chylomicrons	Diacylglycerol (DAG) transported by lipophorin (a reusable lipoprotein shuttle)	Different lipid carriers (lipophorin vs. lipoproteins), but the principle — mobilizing stored TAG as a transportable lipid form — is shared. Lipolysis is the common upstream step.	[56, 57]
**Key lipolytic enzymes**	ATGL (desnutrin), HSL, MGL	Brummer (ATGL ortholog), dHSL; a putative MGL homolog (CG8552/magro) also exists	Sequential TAG to DAG to MAG to glycerol hydrolysis is conserved. Brummer/ATGL catalyzes the rate-limiting first step in both lineages.	[56-58]
**Insulin/IGF signaling (IIS) pathway**	Insulin/IGF-1, IR/IGF-1R, IRS, PI3K, Akt, FOXO	DILPs (*Drosophila* insulin-like peptides), InR, Chico (IRS ortholog), PI3K, Akt, dFOXO	The entire IIS-PI3K-Akt-FOXO axis is conserved; this pathway coordinately regulates BOTH glucose uptake/glycogen synthesis AND lipid synthesis/storage, making it the central node linking carbohydrate and lipid metabolism.	[54]

**Counter-regulatory hormone (glucagon/AKH system)**	Glucagon (pancreatic alpha-cells) activates cAMP/PKA, triggers catabolic programs	AKH (adipokinetic hormone, corpus cardiacum) activates cAMP/PKA, triggers catabolic programs	AKH is the functional ortholog of glucagon; both mobilize glycogen AND lipid stores during fasting, demonstrating parallel carb-lipid catabolic control from a single hormone.	[51, 54]
**Lipid synthesis — transcriptional control**	SREBP-1c (sterol regulatory element-binding protein)	HLH106 / dSREBP	Conserved SREBP-dependent lipogenic program; dSREBP is processed by the same S1P/S2P protease cascade as mammalian SREBP.	[43,83]
**TOR nutrient-sensing pathway**	mTORC1, S6K, 4E-BP1; integrates amino acids, growth factors, energy status	dTORC1, dS6K, 4E-BP (Thor)	TOR is a master integrator: in both species it simultaneously promotes protein/lipid synthesis and suppresses autophagy, coupling carbohydrate sufficiency to anabolic lipid metabolism.	[59, 83]
**AMPK energy sensor**	AMPK inhibits mTORC1, activates catabolic pathways	AMPK (dAMPK-alpha) inhibits dTORC1	AMPK-TORC1 opposition is conserved; AMPK activation simultaneously suppresses gluconeogenesis and lipogenesis while	[59]

			promoting fatty acid oxidation, linking carbohydrate scarcity to lipid catabolism.	
**Brown/beige adipose tissue (adaptive thermogenesis)**	Present (UCP1-mediated non-shivering thermogenesis)	Absent	A genuine mammalian innovation; *Drosophila* lacks this thermogenic fat subtype entirely.	[52]
**Ketogenesis**	Present (hepatic mitochondrial beta-oxidation produces acetoacetate and beta-hydroxybutyrate during prolonged fasting)	Not well established; classical hepatic ketogenesis is absent; limited ketone body metabolism may occur but is poorly characterized	A major metabolic divergence: flies rely predominantly on trehalose mobilization and lipolysis rather than ketone bodies during starvation.	[60]
**Cholesterol / sterol metabolism**	Endogenous cholesterol synthesis via the mevalonate pathway	Cannot synthesize sterols de novo (sterol auxotroph); dietary cholesterol is essential	A critical difference for steroid hormone biology and membrane composition; *Drosophila* requires exogenous sterols.	[10]
**Brain fuel preference (glia-neuron metabolic coupling)**	Glucose is the primary neuronal fuel; the astrocyte-neuron lactate	Glia-derived lactate is a major neuronal fuel; the glia-lactate	Both organisms use glucose AND lactate as neuronal fuels, but their relative contribution differs; the underlying	[10]

	shuttle (ANLS) also operates, especially during high neuronal activity	shuttle plays a prominent role	monocarboxylate transporter machinery is conserved.	
**Response to high-fat diet (HFD)**	Obesity, insulin resistance, hepatic steatosis, lipotoxicity	Obesity, insulin resistance, cardiac dysfunction, ectopic fat deposition	Highly conserved HFD-induced pathophysiology; in both species, excess dietary fat disrupts IIS signaling and drives ectopic lipid storage.	[56-58, 61]
**Lipotoxicity target organs**	Lipid accumulation in liver (NAFLD), heart, pancreas, skeletal muscle	Ectopic lipid storage in fat body (functional equivalent of liver + adipose), heart	Same cellular mechanisms: DAG/PKC impairs insulin signaling; ceramide causes mitochondrial dysfunction; tissue targets differ because the fat body combines hepatic and adipose roles.	[56,57]
**Starvation response**	(1) Glycogenolysis, (2) Gluconeogenesis, (3) Lipolysis (FFA mobilization), (4) Ketogenesis	(1) Glycogenolysis, (2) Trehalose mobilization, (3) Lipolysis (DAG mobilization); gluconeogenesis exists but contributes	Conserved sequential fuel switching (carbohydrate then lipid); the same glucagon/AKH-PKA-ATGL/Brummer cascade triggers lipid mobilization in both lineages.	[51,54]

**Diabetes-like metabolic phenotype**	Insulin resistance, compensatory hyperinsulinemia, beta-cell failure, hyperglycemia + dyslipidemia	less than in mammals DILP imbalance/insulin resistance, hyperglycemia (elevated trehalose + glucose), obesity + dyslipidemia	Both develop insulin resistance, hyperglycemia, and lipid dysregulation through the same IIS-TORC1-FOXO network, validating *Drosophila* as a T2DM model.	[51,54, 62,63]

Table S2 Construction methods, strains, and evaluation indicators of *Drosophila* diabetes models.

Model Type	Construction Method	Experimental Procedure	Key Evaluation Indicators	Refs
HSD induced T2DM	30%–34% sucrose in standard medium 7–14 days	1.Collect 3–5d adults<br>2.Transfer to HSD medium<br>3. Feed 7–14 days<br>4. Change medium every 2–3d	Hemolymph glucose/trehalose ↑<br>Triglyceride ↑ Glycogen ↑<br>Climbing ↓<br>Lifespan ↓<br>SOD/CAT ↓<br>MDA ↑<br>p-Akt ↓<br>FOXO nuclear translocation ↑	[22,33,43]
HFD induced T2DM	20% coconut oil in standard medium<br>7–21 days	1. Collect 3–5d adults<br>2. Transfer to HFD medium<br>3. Feed 7–21 days	Triglyceride ↑ Body weight ↑<br>Insulin resistance<br>Lipid deposition ↑<br>Locomotor activity ↓	[44]

InR Dominant-Negative Mutation	GAL4/UAS-driven *InR^DN^/InR^K1409A^* tissue-specific expression	1. Construct transgenic lines 2. Cross with GAL4 lines 3. Assess progeny phenotype	Blood glucose ↑ Insulin resistance PI3K/Akt signaling ↓ Growth/development retardation	[43]
DILP Cluster Knockout (T1DM)	CRISPR/Cas9 knockout of DILP1-5 gene cluster	1. Design CRISPR sgRNAs 2. Screen mutant lines 3. Validate phenotypes	Hyperglycemia (trehalose ↑) Low insulin signaling Growth retardation Lifespan ↓	[22,44,43]
IPC Ablation (T1DM)	Dilp2-GAL4 driving reaper/hid apoptotic genes specific IPC ablation	1. Acquire lines 2. Cross for progeny 3. Verify IPC ablation efficiency	ILP secretion deficiency Extreme hyperglycemia Severe growth/development impairment	[43]
Chico Knockout/ Knockdown	chico mutant or GAL4/UAS-RNAi tissue-specific KD	1. Acquire mutant/RNAi lines 2. Tissue-specific KD 3. Metabolic phenotype assessment	IRS signaling deficiency Insulin resistance Glucolipid metabolic disorder	[22]
FOXO Over-expression	GAL4/UAS system tissue-specific *FOXO*	1. Construct transgenic lines	*FOXO* target gene activation Gluconeogenesis ↑	[43]

	overexpression or CA	2. Tissue-specific expression<br>3. Assess downstream targets	Insulin signaling inhibition

Table S3. Targets, molecular mechanisms, and experimental results of representative natural products in *Drosophila* diabetes models.

Natural Products	Target/Pathway	Molecular Mechanism	Key Experimental Results	Refs
P. discolor water extract	JAK/STAT<br>PI3K/Akt	Inhibits JAK/STAT pathway<br>Downregulates Imp-L2/Socs36E<br>Restores PI3K/Akt phosphorylation	Glucose/trehalose ↓<br>Lipid ↓<br>FOXO nuclear translocation ↓<br>Body weight/lifespan improved	[79]
20(S)-Ginsenoside Rg3	AMPK/FoxO3/Smad3	Protects mitochondrial function<br>Promotes myoblast differentiation<br>AMPK/FoxO3/Smad3 activation	Muscle atrophy reversed<br>Mitochondrial function restored<br>Myoblast differentiation ↑	[151]
Atractylodes Cuscuta extract	PI3K/Akt<br>GLUT1/3	Activates PI3K/Akt signaling<br>Promotes GLUT1/3 uptake	Insulin sensitivity ↑<br>Blood glucose ↓ Lipid ↓	[69]

		Inhibits FOXO nuclear translocation		
Sanghuang Tongxie Formula	PI3K/Akt	Regulates PI3K/Akt signaling Alleviates insulin resistance	Insulin resistance improved PI3K/Akt signaling restored	[70]
Artocarpus camansi extract	ILP-2/InR /Imp-L2	Modulates ILP-2/InR/Imp-L2 Inhibits α-amylase/ α-glucosidase	ILP-2/InR normalized Imp-L2 ↓ Blood glucose ↓	[71]
Lanhuashen extract	S1P signaling axis	Positively regulates glucolipid metabolism via S1P axis	Glucolipid metabolic disorder improved Insulin sensitivity ↑	[87]
Spondias mombin stem bark extract	ILP-2/InR /Imp-L2	Modulates IIS pathway Improves glucose metabolism	Blood glucose ↓ Glycogen ↓ *ILP-2/InR* normalized	[72]
Sesamin	Nrf2/CncC SOD/CAT	Activates Nrf2/CncC pathway Upregulates SOD/CAT Scavenges cardiac ROS	SOD/CAT ↑ Cardiac ROS ↓ Fractional shortening restored Arrhythmia improved	[103]
Acetobacter + *Lactobacillus*	JNK/JAK/STAT	Blocks JNK-JAK/STAT pathway activation Reduces Imp-L2	JNK phosphorylation ↓ *Imp-L2* ↓ *Socs36E* ↓ Insulin sensitivity restored	[157]
Tyramine	Ca^{2+} signaling	Gut microbiota metabolite Increases Ca^{2+} signaling	Insulin resistance ↓ Ca^{2+} signaling ↑ Metabolic improvement	[81]

Bayberry leaf proanthocyanidins	α-amylase/ α-glucosidase	Inhibits carbohydrate hydrolases Modulates insulin signaling	Blood glucose ↓ Lipid ↓ Antioxidant capacity ↑	[91]
Soy isoflavones	PI3K/Akt FOXO	Upregulates PI3K/Akt phosphorylation Inhibits FOXO target genes Reduces fat body lipid droplets	Glucose/trehalose ↓ PI3K/Akt ↑ Lipid droplets reduced	[76]
Caffeic acid	Nrf2 α-amylase/ α-glucosidase	Activates Nrf2 antioxidant pathway Inhibits carbohydrate hydrolases	Blood glucose ↓ Antioxidant enzymes ↑ Enzyme activity inhibited	[104]
Plumbagin	Nrf2 pathway	Exerts neuroprotection via Nrf2 pathway	Retinal neuron apoptosis ↓ ROS ↓ Visual protection	[103]
Metformin (positive control)	AMPK TORC1	AMPK activation → TORC1 ↓ PEPCK and ACC ↓ DILP2 ↑	Glucose/trehalose/glycogen/ triglyceride/cholesterol ↓ CAT/SOD/GSH ↑	

Table S4: Construction methods, strains, and evaluation indicators of *Drosophila* kidney stone models.

Model Type	Construction Method	Experimental Procedure	Key Evaluation Indicators	Refs
CaOx Stones (NaOx-induced)	0.05%–1% NaOx, 7–14 days	1. Collect 3–5d adults<br>2. NaOx food feeding<br>3. Dissect Malpighian tubules<br>4. Polarized microscopy quantification	Malpighian tubule CaOx crystal count<br>Crystal size (polarized)<br>Excreta crystal content<br>Survival rate	[123]
CaOx Stones (EG-induced)	0.5% ethylene glycol, 21 days	1. EG food feeding 21 days<br>2. Change every 2–3 days<br>3. Polarized/confocal observation	CaOx birefringent crystals<br>Crystal quantification<br>Lifespan (survival curve)<br>Locomotor ability	[114,131]
CaOx Stones (HYP-induced)	0.25%–1% HYP	1. HYP food feeding<br>2. Dissect Malpighian tubules<br>3. Metabolite analysis	CaOx crystal quantification<br>Hydroxyproline metabolites<br>Malpighian morphology	[123]
Uric Acid (UA) Stone Model	*Uro* gene KO+ high-purine diet	1. *Uro* KO lines<br>2. High-purine diet feeding<br>3. SEM/EDS/confocal verification	Malpighian tubule UA crystals<br>SEM/EDS elemental analysis<br>Confocal imaging<br>qRT-PCR validation	[123]

Endogenous Oxalate Accumulation	*dAGXT* RNAi (alanine-glyoxylate aminotransferase KD)	1. Malpighian tubule *dAGXT* KD<br>2. Oxalate detection<br>3. Crystal formation assessment	Endogenous oxalate ↑<br>CaOx spontaneous formation<br>Enzyme activity ↓	[123]

Table S5: Targets, molecular mechanisms, and experimental results of representative NPs in *Drosophila* kidney stone models.

Natural Products	Target/Pathway	Molecular Mechanism	Key Experimental Results	Refs
A. membranaceus extract	CaOx crystal formation<br>Oxidative stress	Preventively inhibits CaOx crystal nucleation (not dissolution)<br>Antioxidant protection	16 mg/mL CaOx crystals ↓<br>Lifespan shortening reversed<br>Cannot dissolve formed crystals in vitro	[131]
Tripotassium hydroxycitrate (K-HCA)	CaOx crystal nucleation/growth	Directly inhibits CaOx crystal nucleation and growth	Dose-dependent crystal ↓<br>Effective at 0.01%<br>Superior to equal conc. K-citrate	[115]
Salvianic acid A (SAA)	ROS/CaOx crystal	Inhibits CaOx crystal growth<br>Reduces ROS<br>Upregulates antioxidant enzymes	CaOx crystals ↓<br>ROS ↓ Antioxidant enzymes ↑<br>Validated in mouse model	[117]
Hydroxysafflor yellow A (HYA)	Oxidative stress<br>CaOx crystal	Inhibits CaOx crystal nucleation and growth	CaOx crystals ↓<br>ROS ↓	[117]

		Reduces epithelial cell ROS	Antioxidant enzymes ↑ Validated in mouse model	
Hesperidin	CaOx crystallization Antioxidant	Inhibits CaOx crystallization Antioxidant protection	CaOx crystals ↓ Mouse model validated in parallel	[237]
Kukoamine A	Endogenous oxalate synthesis	Inhibits endogenous oxalate synthesis pathway	Oxalate synthesis ↓ Crystal formation ↓	
Potassium citrate (positive control)	CaOx crystallization	Alkalinization equivalent Inhibits CaOx crystallization	Significantly inhibits EG-induced CaOx crystal formation Reference standard in multiple studies	[131]

Table S6: Construction methods, strains, and evaluation indicators of *Drosophila* IBD models.

Model Type	Construction Method	Experimental Procedure	Key Evaluation Indicators	Refs
DSS-Induced Acute Colitis	3%–7% DSS (w/v)<br>MW 36–50 kDa in 5% sucrose solution or standard food<br>48–72h to 5–7d	1. Collect 3–5d adults<br>2. DSS food feeding 48–72h to 5–7d<br>3. Phenotype assessment	Smurf positive rate (barrier ↓)<br>Intestinal length ↓<br>ISC proliferation (PH3+ ↑)<br>MDA ↑ ROS ↑<br>AMPs ↑ Apoptotic genes ↑	[28]
SDS-Induced Enteritis	0.5%–0.6% SDS in standard food<br>48–72 h	1. SDS food feeding<br>2. Intestinal morphology/barrier<br>3. Oxidative stress/inflammation	Intestinal edema<br>Epithelial apoptosis<br>Intestinal length ↓<br>Barrier permeability ↑<br>MDA/ROS ↑<br>SOD/CAT ↓	[28]
Paraquat-Induced Oxidative Stress IBD	Paraquat feeding	1. Paraquat food feeding<br>2. ROS/DHE staining<br>3. Antioxidant enzyme activity	Intestinal ROS excessive accumulation<br>Lipid peroxidation ↑<br>Antioxidant enzymes ↓	[28]
EBV-DNA Synergistic IBD Exacerbation	EBV viral DNA + DSS combined feeding	1. EBV-DNA pretreatment<br>2. DSS combined feeding<br>3. Hindgut inflammation assessment	Hindgut inflammation exacerbated<br>IMD pathway hyperactivation<br>AMPs significantly ↑	[142]
Atg16 Mutation Genetic IBD	*Atg16* WD40 domain	1. Acquire mutant lines	EE differentiation defect	[144]

	deletion mutants (*Atg16^{MI}*, *Atg16^{d67}*)	2. Intestinal phenotype assessment<br>3. EE differentiation/Slit detection	Slit/Robo signaling ↓<br>Spontaneous chronic intestinal inflammation<br>Enterobacteriaceae abnormal proliferation	
GlcAT-S Silencing Mucus Protection Defect	Intestine-specific *GlcAT-S* RNAi	1. *GlcAT-S* KD<br>2. Mucus layer assessment<br>3. Epithelial damage detection	M*uc68D*/*Mur29B* ↓<br>Mucus protective layer disrupted<br>Epithelial damage ↑<br>Inflammation ↑	[146]
Imd Pathway Activation Genetic IBD	Intestine-specific Imd overexpression or Diptericin activation	1. Intestinal Imd overexpression<br>2. AMP detection<br>3. Inflammation assessment	AMPs (Dpt/AttA/Mtk) ↑<br>NF-κB/IMD target gene activation<br>Chronic intestinal inflammation	[146]
Microbiota Dysbiosis Model (Antibiotic + Pathogen)	Broad-spectrum Abx clearance → colonize *P. aeruginosa*/ *E. faecalis*/Ecc15	1. Abx microbiota clearance<br>2. Pathogen colonization<br>3. Microbiota-inflammation interaction	Microbiota imbalance<br>Lipid droplet abnormal accumulation<br>AMP overexpression chronic persistent inflammation	[139]
Ara-C Chemotherapy Intestinal Injury	Cytarabine (Ara-C) 1–10 mM feeding	1. Ara-C food feeding<br>2. ROS/apoptosis detection<br>3. Imd/Toll pathway analysis	ROS burst<br>Imd/Toll dual-pathway activation<br>Reaper/Dcp-1/Drice ↑<br>Epithelial apoptosis ↑	[143]
Chronic Sleep Deprivation (CSD) IBD Model	Mechanical vibration sleep deprivation	1. Sleep deprivation treatment<br>2. Intestinal barrier/inflammation	IMD pathway activation<br>PGRP-SB1/Dpt/AttA ↑	[147]

+ intestinal assessment	3. Imd pathway activation assessment	Intestinal barrier damage "Gut-Sleep-Inflammation" axis

Table S7: Targets, molecular mechanisms, and experimental results of representative NPs in *Drosophila* IBD Models.

Natural Product	Target/Pathway	Molecular Mechanism	Key Experimental Results	Refs
Caffeic acid	IMD pathway NF-κB	Specifically inhibits IMD pathway Downregulates PGRP-SB1/Dpt/ AttA/Mtk Suppresses ISC hyperproliferation	AMPs ↓ ISC normalized Epithelial apoptosis ↓ CSD/DSS intestinal recovery "Gut-Sleep-Inflammation" axis	[147]
Bilberry anthocyanins	Nrf2/Keap1 Antioxidant	Activates Nrf2 pathway Upregulates GCL/GSTS/SOD Scavenges ROS Regulates microbiota	ROS ↓ MDA ↓ SOD/CAT ↑ Intestinal barrier repaired Microbiota dysbiosis regulated	[140]
Flos puerariae extract	Nrf2-Keap1 JAK-STAT Wnt	Simultaneously regulates three pathways Inhibits ISC hyperproliferation Restores cell differentiation lineage	ISC proliferation ↓ (PH3+ ↓) Antioxidant ↑ Cell differentiation normalized Wnt signaling rebalanced	[141]
A. membranaceus	JNK JAK-STAT Nrf2	Multiple bioactive compounds	JNK/JAK-STAT ↓	[149]

extract		(formononetin/isoliquiritigenin/ isorhamnetin/astragaloside/ vanillic acid/caffeic acid) Multi-target anti-inflammatory and antioxidant	Oxidative stress ↓ Intestinal morphology improved Barrier function ↑	
Total ginsenosides	MAPK (p-JNK/ p-ERK)	Downregulates p-JNK/p-ERK Inhibits abnormal MAPK activation	p-JNK ↓ p-ERK ↓ Intestinal inflammation repaired SDS damage alleviated	[151]
Hylotelephium Erythrostictum extract	ROS/Multiple pathways	Scavenges ROS Inhibits multiple inflammatory signaling pathways	ROS ↓ Multi-pathway inflammation ↓ Intestinal protection	[145]
Luteolin	IMD/NF-κB	Inhibits IMD/NF-κB pathway Reduces AMP expression	AMP dpta ↓ Intestinal inflammation ↓ Smurf permeability ↓	[30]
Ursolic acid	NF-κB (IMD) ISC proliferation	Inhibits NF-κB pathway Suppresses ISC hyperproliferation	PH3+ cells ↓ AMPs ↓ Intestinal morphology restored	[30]
Safranal	IMD/NF-κB Nrf2	Dual mechanism: IMD inhibition + Nrf2 antioxidant activation	Smurf positive rate ↓ Survival rate ↑ Inflammation ↓ Antioxidant ↑	[30]

Danggui Buxue Decoction (DBD)	Toll/IMD dual pathway	Resveratrol + magnolol simultaneously inhibit Toll/IMD	Toll/IMD ↓<br>Intestinal neuro-immune improvement<br>Sleep + intestinal inflammation ↓	[147]
GlcAT-S over-expression (mucus protection)	Mucin genes Muc68D/Mur29B	Upregulates mucin genes<br>Strengthens mucus protective layer	Muc68D/Mur29B ↑<br>Intestinal length maintained<br>ISC normalized<br>Inflammation ↓	[146]

Table S8. Herbal Composition of Traditional Chinese Medicine Compound Formulas in This Review

Formula Name / Herb (Chinese)	Chinese Name	Pinyin Name
**Xuanfei Baidu Decoction (XFBD)** 宣肺败毒方 (Xuān Fèi Bài Dú Fāng)	黄芪	Huang Qi
	防风	Fang Feng
	荆芥	Jing Jie
	苦参	Ku Shen
	金银花	Jin Yin Hua
	连翘	Lian Qiao
	桑白皮	Sang Bai Pi
	薄荷	Bo He
	甘草	Gan Cao
	苦杏仁	Ku Xing Ren
	大青叶	Da Qing Ye
	青蒿	Qing Hao
	灯芯草	Deng Xin Cco
**Danggui Buxue Decoction** 当归补血汤 (Dāng Guī Bǔ Xuè Tāng)	黄芪	Huang Qi
	当归	Dang Gui
**Xuefu Zhuyu Decoction** 血府逐瘀汤 (Xuè Fǔ Zhú Yū Tāng)	桃仁	Tao Ren
	红花	Hong Hua
	当归	Dang Gui
	生地黄	Sheng Di Huang
	牛膝	Niu Xi
	赤芍	Chi Shao
	枳壳	Zhi Qiao
	甘草	Gan Cco
	桔梗	Jie Geng
	川芎	Chuan Xiong

	柴胡	Chai Hu
**Sanghuang Tongxie Formula** 桑黄通泄方 (Sāng Huáng Tōng Xiè Fāng)	桑白皮	Sang Bai Pi
	黄连	Huang Lian
	厚朴	Hou Po
	葛根	Ge Gen
	黄芪	Huang Qi
	山萸肉	Shan Yu Rou
	莱菔子	Lai Fu Zi
	知母	Zhi Mu
	玉竹	Yu Zhu
	苍术	Cang Zhu

Table S9: Construction methods, strains, and evaluation indicators of *Drosophila* cancer models.

Model Type	Construction Method	Experimental Procedure	Key Evaluation Indicators	Refs
RasV12+scrib-/- Invasive Tumor	GAL4/UAS-driven *RasV12* overexpression + scrib KO	1. Construct multi-gene combination<br>2. Tissue-specific expression<br>3. Tumor growth/invasion assessment	Tumor size<br>Invasiveness<br>Metastasis<br>Lethality<br>Drug sensitivity	[167]
RAS/PTEN Lung Cancer	Tracheal btl-GAL4 driving *RASG12V+PTEN* RNAi	1. Construct dual-gene model<br>2. Drug administration larval/adult<br>3. Drug screening	Tracheal tumor growth<br>Larval lethality<br>Drug synergy effects	[168]
EGFR Lung Cancer	Constitutively active *EGFR* tracheal expression	1. *EGFR* activating expression	EGFR-induced lethality	[168]

		2. FDA drug library screening 3. Synergistic regimen validation	Tumor proliferation Signaling pathway inhibition	
MEN2B RET Medullary Thyroid Carcinoma	*dRet*^{MEN2B} transgene (M918T)	1. Construct transgenic line 2. Drug dose-response assessment 3. Phenotype rescue	Lethality Developmental defects RET signaling activity Drug dose optimization	[176]
PDAC Pancreatic Cancer "4-hit"	KRAS/TP53/ CDKN2A/SMAD4 four-gene mutation	1. Whole-kinome genetic screen 2. Combined inhibition validation 3. Xenograft confirmation	Tumor growth Lethality Kinome screen Human PDAC cell validation	[104][209]
Fly Avatar Colorectal Cancer	Patient-specific 9-gene mutation combination (KRAS/p53/PTEN/ APC/SMAD4 etc.)	1. Introduce patient mutations 2. FDA drug library screen (121 drugs) 3. Optimal regimen recommendation	Tumor burden ↓ (45%)	[102][217]
scrib Knockdown Epithelial Cancer	*scrib* gene RNAi KD (JNK-MMP1 pathway)	1. scrib KD 2. Tumor invasion assessment 3. Natural product screening	Epithelial structure disruption Invasive tumor JNK/MMP1 activation	[186]

RasV12-Driven Tumor Model (Vitamin B3 study)	RasV12 overexpression + Vitamin B3 intervention	1. RasV12 overexpression 2. Vitamin B3 intervention 3. Redox/autophagy detection	Tumor growth ↓ Redox regulation Mitochondrial function ↑ Autophagy activation	[184]

Table S10: Targets, molecular mechanisms, and experimental results of representative NPs in *Drosophila* cancer models.

Natural Product	Target/Pathway	Molecular Mechanism	Key Experimental Results	Refs
Trametinib + Fluvastatin	MEK HMG-CoA reductase	MEK inhibition + statin synergistically suppress RAS/PTEN tumors	Tracheal tumor ↓ Lethality ↓ A549 NSCLC proliferation ↓ 1,192 FDA drugs screened	[198]
Trametinib + Zoledronate	MEK + bone metabolism	Optimal combination from 121 FDA drugs Fly Avatar platform	Tumor burden ↓ 45%	[168]
Vandetanib	RET/VEGFR /EGFR	Multi-target TKI RET^{M918T} inhibition Dose-response relationship	Lethality ↓ Dev. defects ↓ Dose-dependent rescue Dosing regimen parameters optimized	[176]
Afatinib + Bazedoxifene	EGFR JAK/STAT	EGFR TKI + SERM (bazedoxifene) Synergy via JAK/STAT	~1,000 FDA drugs screened EGFR lethality ↓ JAK/STAT signaling ↓	[168]
Vitamin B3	Redox Mitochondria	Redox regulation	RasV12 tumor growth ↓ ROS ↓ Mitochondria ↑	[184]

	Autophagy	Mitochondrial function ↑<br>Autophagy activation	Autophagy markers ↑	
Physalin A	Tumor invasion<br>Notch	Inhibits tumor invasion<br>Modulates Notch signaling	Tumor invasion ↓<br>Notch signaling ↓<br>MDA-MB-231 cells	[185]
4H-chromene Derivatives (anti-metastatic)	JNK<br>MMP1	Targets JNK-MMP1<br>Inhibits tumor invasion<br>and metastasis	Tumor invasion ↓<br>JNK/MMP1 ↓<br>Metastasis ↓	[186]

Table S11: Construction methods, strains, and evaluation indicators of *Drosophila* AD models.

Model Type	Construction Method	Experimental Procedure	Key Evaluation Indicators	Refs
Aβ42 Neuronal Expression	*elav-GAL4* driving *UAS-Aβ42* (human) pan-neuronal expression	1. Construct/acquire transgenic lines<br>2. Cross for progeny<br>3. Continuous adult assessment<br>4. Drug intervention	Climbing ability ↓<br>Lifespan ↓<br>Brain Aβ42 deposition<br>Oxidative stress ↑<br>AChE activity ↑	[203]
Aβ42 Eye Expression	*GMR-GAL4* driving *UAS-Aβ42* eye expression	1. Eye-specific expression<br>2. Rough eye phenotype assessment<br>3. Rapid genetic/drug screening	Rough eye phenotype<br>Photoreceptor neuron degeneration<br>Rapid screening readout	[203,204]
Tau Protein Pathology	Neuronal expression human tau (R406W or P301L mutation)	1. *Tau* transgene expression<br>2. Tau phosphorylation detection<br>3. Microtubule function assessment	Tau hyperphosphorylation<br>Axonal transport defects<br>Neurodegeneration<br>Lifespan ↓	[205]

Aβ+tau Combined Model	*Aβ42+tau* dual transgene combined expression	1. Dual transgenic lines<br>2. Composite phenotype assessment<br>3. Multi-target drug screening	Aβ deposition + tau phosphorylation<br>Composite neurodegeneration<br>More comprehensive AD simulation	[207]
Aβ42 Gut Expression	Intestine-specific Aβ42 expression digestive tract proteotoxicity	1. Intestinal *Aβ42* expression<br>2. Digestive tract toxicity assessment<br>3. Gut-brain axis studies	Digestive tract Aβ proteotoxicity<br>Gut-brain axis communication abnormalities	[204]

Table S12: Construction methods, strains, and evaluation indicators of *Drosophila* PD models.

Model Type	Construction Method	Experimental Procedure	Key Evaluation Indicators	Refs
α-Synuclein Overexpression	Neuronal or DDC neuron-specific overexpression of human *α*Syn	1. *αSyn* transgene expression<br>2. DA neuron counting<br>3. Motor behavior tests	DA neuron degeneration/death<br>TH expression ↓<br>Locomotor ↓<br>Lewy body-like aggregates<br>Lifespan ↓	[208]
PINK1^{B9} Mutation	PINK1 loss-of-function mutation (PINK1^{B9}) mitophagy deficiency	1. Acquire *PINK1^{B9}* line<br>2. Motor/mitochondrial assessment<br>3. Drug intervention	Climbing ↓<br>Lifespan ↓<br>Mitochondrial morphology abnormal<br>ATP ↓ DA levels ↓<br>Muscle atrophy	[211,212]
Parkin LOF Mutation	parkin loss-of-function mutation (park^{25} etc.) mitophagy deficiency	1. Acquire parkin mutant line<br>2. Oxidative stress assessment<br>3. Mitochondrial function detection	Oxidative stress ↑<br>Mitochondrial dysfunction<br>Locomotor ↓<br>Lifespan ↓<br>H_2O_2/NO/MDA ↑	[211]
LRRK2 G2019S	Overexpression of G2019S mutant LRRK2 enhanced kinase activity	1. LRRK2 mutant expression<br>2. Kinase activity detection<br>3. DA neuron assessment	LRRK2 kinase activity ↑<br>DA neuron degeneration<br>Motor deficits	[210]
DJ-1 Double KO	*DJ-1α*/*DJ-1β* double gene KO	1. Double KO line<br>2. Oxidative stress sensitivity	Oxidative stress sensitivity ↑<br>Mitochondrial function ↓	[213]

	oxidative stress sensitive	3. Mitochondrial function	Motor deficits	
Rotenone-Induced Sporadic PD	Rotenone Complex I inhibitor in food	1. Rotenone food feeding<br>2. Age-dependent assessment<br>3. DA neuron detection	DA neuron selective death<br>Complex I activity ↓<br>Locomotor ↓<br>Oxidative stress ↑<br>Age-dependent	[216,217]
Paraquat-Induced Oxidative Stress PD	Paraquat oxidative stress inducer in food	1. Paraquat feeding<br>2. Oxidative stress assessment<br>3. Neuroprotective drug screening	ROS excessive production<br>DA neuron damage<br>Motor deficits<br>SOD/CAT ↓	[216]
MPTP-Induced PD Model	MPTP (mitochondrial toxin) treatment DA neuron selective damage	1. MPTP treatment<br>2. TH+ neuron counting<br>3. Motor/mitochondrial assessment	DA neuron degeneration<br>TH expression ↓<br>Locomotor ↓<br>Mitochondrial dysfunction	[218]
Synphilin-1 Overexpression	*Synphilin-1* overexpression Parkinsonian phenotype	1. Synphilin-1 expression<br>2. DA/5-HT system assessment<br>3. Nicotine intervention	Parkinsonian phenotype<br>DA/5-HT circuitry involvement<br>Nicotine suppresses phenotype	[209]

Table S13: Targets, molecular mechanisms, and experimental results of representative NPs in *Drosophila* AD models.

Natural Product	Target/Pathway	Molecular Mechanism	Key Experimental Results	Refs
Vitamin K2	Autophagy<br>Mitochondria	Activates autophagy (*LC3*↑/ *Beclin1*↑)<br>Improves mitochondrial function	Climbing ↑<br>Lifespan ↑<br>Brain Aβ42 ↓<br>NDUFS3/ATP ↑	[228]
Apigenin	*Aβ42* aggregation<br>AChE<br>Oxidative stress	Inhibits *Aβ42* aggregation (immunostaining + docking)<br>Inhibits *AChE* activity	Climbing decline delayed<br>Oxidative stress ↓<br>*AChE* activity ↓ (dose-dependent)	[137]
p-Coumaric acid	Aβ42 fibrillation<br>Cell death	Inhibits *Aβ42* fibrillation in vitro (ThT assay)<br>PC12 cell protection	*Aβ42* fibrillation ↓<br>Rough eye partially reversed<br>Lifespan ↑<br>Locomotor ↑ (sex-dependent)	[219]
Ethyl caffeate	*Aβ42* toxicity<br>Oxidative stress	Reduces *Aβ42* toxicity<br>PC12 cell protection	Rough eye improved<br>Lifespan ↑ Midlife locomotor ↑<br>PC12 cell death ↓	[220]
Fenugreek leaf extract	*AChE MAO caspase-3/9*<br>Oxidative stress<br>Metabolome	Dose-dependent multi-target<br>*AChE* ↓ *MAO* ↓ *caspase* ↓ Antioxidant<br>22 differential metabolites	Climbing decline delayed (dose-dep.)<br>Cognitive function ↑<br>Lifespan ↑ *AChE* ↓<br>*Aβ42* fiber deposition ↓	[221]
Quercetin	Cell cycle protein expression	Modulates cell cycle-related protein expression	Aβ toxicity ↓<br>Cell cycle proteins normalized	[222]

Methylene blue	MARK4/PAR1 Tau phosphorylation	Attenuates MARK4/PAR1-mediated tau phosphorylation	Tau phosphorylation ↓<br>Synaptic toxicity ↓	[223]
Jatamansinol	Aβ42/Tau toxicity	Protects against Aβ42 and tau-induced neurotoxicity	Aβ42 toxicity ↓<br>Tau toxicity ↓	[224]
Resveratrol + Sir2	Sir2<br>Sleep/memory	Activates *Sir2*<br>Ameliorates Aβ-induced<br>sleep and memory deficits	Sleep ↑ Memory ↑<br>Aβ pathology ↓	[235]

Table S14: Targets, molecular mechanisms, and experimental results of representative NPs in *Drosophila* PD models.

Natural Product	Target/Pathway	Molecular Mechanism	Key Experimental Results	Refs
Kaempferol	αSyn aggregation<br>TH expression<br>Oxidative stress	Directly binds αSyn (molecular docking)<br>Inhibits aggregation<br>Increases TH expression	Climbing decline delayed (dose-dep.)<br>Oxidative stress ↓<br>TH expression ↑	[148]
Resveratrol	Oxidative stress<br>TH/ple/Sod1<br>Mitochondria	H_2O_2/NO/MDA ↓<br>*ple*/*Sod1* mRNA ↑<br>Mitochondrial fluorescence ↑	Survival ↑ Climbing ↑<br>Oxidative stress ↓<br>TH restored<br>Mitochondria ↑	[235]
Resveratrol + Levodopa	MAO/GSK-3<br>αSyn binding	Network pharmacology + docking<br>Identified	Lifespan ↑ Locomotor ↑<br>AChE ↑ CAT ↑ Thiols ↑<br>NO/MDA/hydroperoxide ↓	[235]

		MAO/GSK-3 as core targets<br>Inhibits aggregation via SNCA binding	*Sod1* mRNA ↑	
Saffron/Crocetin	αSyn aggregation<br>Neuroprotection	Effective in both mutant models<br>Cytoprotective effects	*A30P*/*G51D* climbing ↑<br>G51D lifespan ↑<br>Rough eye ↓<br>Human neuron protection	[236]
Hesperidin	Aβ+αSyn dual protection	AD+PD dual model<br>Broad-spectrum neuroprotection	Fecundity ↑ Larval locomotion ↑<br>Climbing ↑ (dose-dependent)<br>Broad-spectrum neuroprotection	[237]
Propolis + Levodopa	Antioxidant<br>Synergistic enhancement	Propolis alone improves climbing/ antioxidant/lifespan<br>Combined high conc. synergistic	Climbing ↑ Antioxidant ↑<br>Lifespan ↑<br>Levodopa side effects ↓	[238]
Lycium barbarum fruit extract	Mitochondrial Function<br>ATP/DA<br>Neuroprotection	ATP/DA ↑ at all time points<br>SH-SY5Y cell MPP+ toxicity rescue	ATP ↑ DA ↑<br>Lifespan ↑ Locomotor ↑<br>Olfaction ↑ Muscle atrophy repair<br>Cytoprotection	[229]
Caffeine	Mitochondrial function<br>Complex I	Improves mitochondrial function<br>Complex I activity ↑	Mitochondrial function ↑<br>Motor improvement	[231]

Ginseng protein	*UPR^{mt}* Mitochondria	Induces mitochondrial unfolded protein response (*UPR^{mt}*)	Mitochondrial dysfunction ↓ Neuroprotection	[230]
Grape skin extract	Mitophagy Muscle function	Activates mitophagy Improves muscle function	Lifespan ↑ Muscle function ↑ Mitophagy ↑	[230,231]
Ellagic acid	Mitochondrial Function Oxidative stress	Modulates mitochondrial function Alleviates oxidative damage	Rotenone damage ↓ Mitochondrial function ↑	[239]
Naringenin	Antioxidant Mitochondrial protection	Antioxidant Mitochondrial protection Combined with computational biology	MPTP toxicity ↓ DA neuron protection	[218]
Calycosin	Mitochondrial Function Autophagy	Improves mitochondrial function Regulates autophagy	Paraquat neurodegeneration ↓ Mitochondria ↑ Autophagy ↑	[233]
Curcumin	Ubiquitination Oxidative stress	Reverses dUCH KD-induced PD-like phenotype Antioxidant and anti-inflammatory	PD-like phenotype reversed Oxidative stress ↓ Motor function ↑	[201]

Table S15. Comprehensive classification of natural products cited in this review: chemical classes, mechanisms of action, and disease associations.

Chemical Class / Subclass	Compound Name Structural & Formula	Disease(s)	Mechanism of Action & Molecular Target(s)
**Alkaloids**	Berberine<br>$C_{20}H_{18}NO_4^+$<br>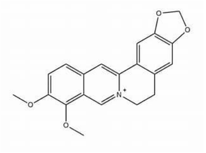	Diabetes,	Classic AMPK agonist → inhibits TORC1, represses pepck/acc transcription; relieves hyperglycemia, insulin resistance, and excessive lipogenesis in HSD flies. Also modulates IIS/PI3K/Akt/FOXO cascade[82].
**Alkaloids**	Piperine<br>$C_{17}H_{19}NO_3$<br>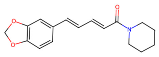	Cancer, IBD	Suppresses clonal tumor expansion in wts-mutant epithelia by antagonizing aberrant Yki (Hippo pathway) activity → reduces DIAP1 and stg (string) expression. Also modulates JNK/JAK and apoptosis pathways[190].
**Alkaloids**	4,5-Dihydroxypiperine<br>$C_{17}H_{19}NO_5$<br>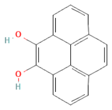	Cancer	Piperine derivative; antagonizes aberrant Yki activity, suppresses clonal tumor expansion in wts-mutant *Drosophila* epithelia[190].
**Alkaloids**	Caffeine<br>$C_8H_{10}N_4O_2$<br>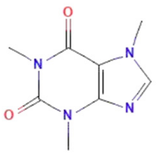	PD	Enhances mitochondrial complex activity, induces UPRmt, promotes mitophagy, and supports neuronal survival in PD fly models[231].
**Alkaloids**	Kukoamine A<br>$C_{28}H_{42}N_4O_6$<br>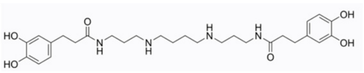	Nephrolithiasis	Downregulates DAO (D-amino acid oxidase) expression via IL-6/JAK/STAT3 activation → reduces endogenous oxalate synthesis. Also relieves oxidative tissue injury.

			Validated in both *Drosophila* nephrolithiasis and mouse models[118].
**Flavonoids**	Solanines<br>$C_{45}H_{73}NO_{15}$<br>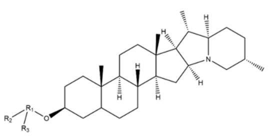	Diabetes	Exhibit hormetic profile: low doses trigger protective Nrf2-mediated antioxidant responses and reduce genotoxicity; high doses cause cytotoxicity and genotoxicity. *Drosophila* studies remain limited; conserved antioxidant and DNA damage pathways in flies support future investigation[105,106]
**Flavonoids**	Luteolin<br>$C_{15}H_{10}O_6$<br>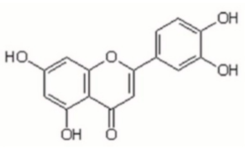	Diabetes, IBD	Diabetes: modulates JNK/JAK/STAT, Nrf2/Keap1, mTOR pathways; IBD: blocks IMD/NF-κB cascades, reduces antimicrobial peptides; Nephrolithiasis: reverses oxalate-induced ROS overproduction, restores redox homeostasis in Malpighian tubules [30,118].
**Flavonoids**	Apigenin<br>$C_{15}H_{10}O_5$<br>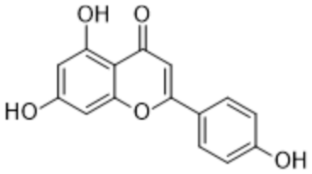	AD	Dose-dependently suppresses Aβ42 aggregation, reduces AChE activity, relieves oxidative stress, and mitigates locomotor decline in AD flies[137][162].
**Flavonoids**	Luteolin-7-O-diglucuronide<br>$C_{27}H_{26}O_{18}$<br>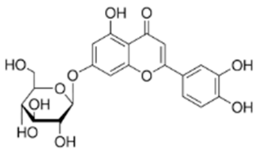	Diabetes	From Perilla frutescens var. acuta; demonstrated anti-adipogenic and thermogenic activities in vitro. In vivo validation in *Drosophila* pending[98].

**Flavonoids**	Apigenin-7-O-diglucuronide<br>$C_{27}H_{26}O_{17}$<br>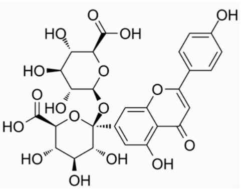	Diabetes	From Perilla frutescens var. acuta; demonstrated anti-adipogenic and thermogenic activities in vitro. In vivo validation in *Drosophila* pending[98].
**Flavonoids**	Quercetin<br>$C_{15}H_{10}O_7$<br>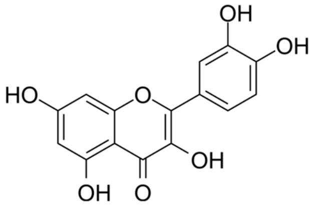	Diabetes, IBD, Nephrolithiasis, AD	Diabetes: IIS/PI3K/Akt, Nrf2/Keap1, mTOR/TORC1 regulation; IBD: reduces ROS, restores Atg16/Slit → stabilizes ISC lineage differentiation; Nephrolithiasis: reverses oxalate-induced ROS; AD: relieves $A\beta$ toxicity via Cyclin B regulation; cholinesterase modulation [135,162,163].
**Flavonoids**	Kaempferol<br>$C_{15}H_{10}O_6$<br>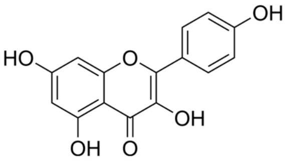	Cancer, PD	Cancer: modulates mTOR/TORC1, apoptosis pathway; PD: directly binds α-synuclein → inhibits aggregation, reduces oxidative stress, restores TH expression, improves locomotor deficits[148][226].
**Flavonoids**	Naringenin<br>$C_{15}H_{12}O_5$<br>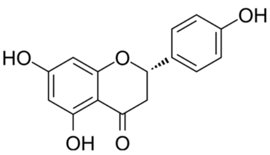	PD	PI3K/Akt and Nrf2/Keap1 modulation; broad neuroprotection via ROS scavenging, mitochondrial function maintenance, and autophagic flux regulation in PD models[218].
**Flavonoids**	Hesperidin<br>$C_{28}H_{34}O_{15}$<br>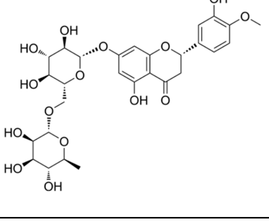	PD	Broad neuroprotection in toxin-induced PD models: ROS scavenging, mitochondrial function maintenance, autophagic flux regulation, and inhibition of neuronal apoptosis[237,163].

**Flavonoids**	Genistein<br>$C_{15}H_{10}O_5$<br>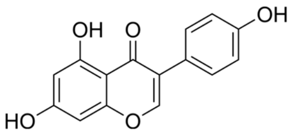	Diabetes,	Insulin-sensitizing via PI3K/AKT pathway modulation; also modulates mTOR, JNK/JAK/STAT pathways. Soy isoflavone fraction with hydroxylated derivatives confirmed antidiabetic in *Drosophila* HSD model[74].
**Flavonoids**	Daidzein<br>$C_{15}H_{10}O_4$<br>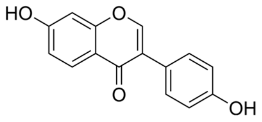	Diabetes	Soy isoflavone with insulin-sensitizing properties via PI3K/AKT pathway modulation in mammalian models. *Drosophila*-specific data pending[73-75].
**Flavonoids**	Biochanin A<br>$C_{16}H_{12}O_5$<br>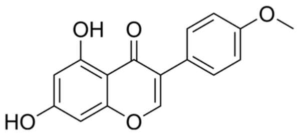	Diabetes	Soy-derived O-methylated isoflavone; insulin-sensitizing effects through PI3K/AKT signaling pathway modulation in mammalian models[73].
**Flavonoids**	Glycitein<br>$C_{16}H_{12}O_5$<br>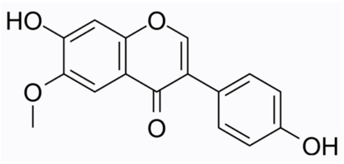	Diabetes	Soy isoflavone with insulin-sensitizing properties via PI3K/AKT pathway; validated in mammalian models[73-75].
**Flavonoids**	Formononetin<br>$C_{16}H_{12}O_4$<br>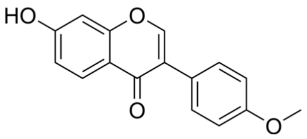	IBD	Bioactive constituent of Astragalus membranaceus; synergistically inhibits JNK and JAK/STAT signaling along with other Astragalus compounds (isoliquiritigenin, astragalosides, caffeic acid)[149].
**Flavonoids**	Calycosin<br>$C_{16}H_{12}O_5$<br>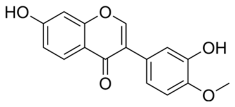	PD	Inhibits dopaminergic neuron death via enhancing mitophagy and autophagic flux; suppresses mTOR/S6K/4EBP1 signaling; restores

			energy and catabolic homeostasis in paraquat-induced PD flies[233].
**Flavonoids**	Calycosin-7-O-β-D-glucoside<br>$C_{22}H_{22}O_{10}$<br>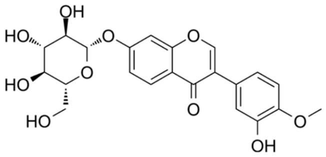	Nephrolithiasis	Suppresses CaOx crystal formation, modulates crystal growth, and alleviates oxidative stress in *Drosophila* Malpighian tubules. Efficacy validated in both fly and mouse nephrolithiasis models[118].
**Flavonoids**	Epigallocatechin gallate<br>$C_{22}H_{18}O_{11}$<br>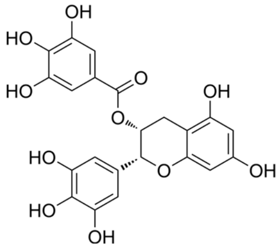	Cancer, AD	Cancer: targets glycolytic regulators HK II and PKM2 in mammalian models; Neurodegeneration: Nrf2/Keap1 activation, mTOR/TORC1 modulation; Epigenetic: influences DNA methylation, histone acetylation in oncogenesis[201].
**Flavonoids**	Isoliquiritigenin<br>$C_{15}H_{12}O_4$<br>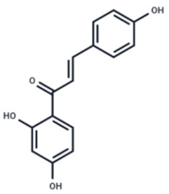	IBD	Bioactive constituent of Astragalus membranaceus; synergistically inhibits JNK and JAK/STAT signaling[149].
**Flavonoids**	Hydroxysafflower Yellow A<br>$C_{27}H_{32}O_{16}$<br>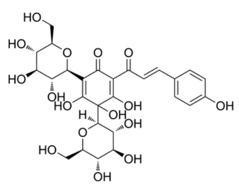	Nephrolithiasis	Suppresses CaOx crystal formation and modulates crystal growth in *Drosophila* Malpighian tubules; alleviates oxidative stress. Efficacy validated in both fly and mouse nephrolithiasis models[119,128].
**Phenolic acids**	Caffeic acid<br>$C_9H_8O_4$<br>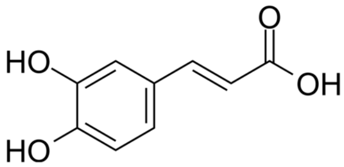	Diabetes, IBD, Cancer	Diabetes: activates Nrf2-dependent antioxidant defenses, inhibits α-amylase/α-glucosidase; IBD: suppresses excessive IMD/NF-κB activation, inhibits abnormal ISC

			proliferation and epithelial apoptosis; Cancer: anti-inflammatory activity[104,147,155,156].
**Phenolic acids**	Rosmarinic acid<br>$C_{18}H_{16}O_8$<br>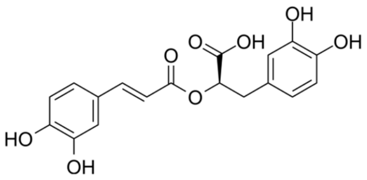 	Diabetes, Nephrolithiasis	Diabetes: anti-adipogenic and thermogenic activity (in vitro) from Perilla frutescens; Nephrolithiasis: Ca^{2+} chelation via carboxylate/hydroxyl groups reduces free Ca^{2+} for CaOx nucleation and growth[126].
**Phenolic acids**	Chlorogenic acid<br>$C_{16}H_{18}O_9$<br>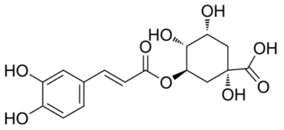 	Nephrolithiasis	Phenolic acid with strong Ca^{2+}-chelating ability → sequesters free Ca^{2+}, reduces CaOx nucleation and crystal growth[126].
**Phenolic acids**	p-Coumaric acid<br>$C_9H_8O_3$<br>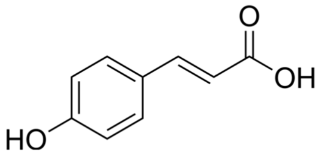 	AD	Blocks Aβ42 fibrillation in vitro, mitigates Aβ42 toxicity, and extends lifespan in *Drosophila* Aβ42 models[219].
**Phenolic acids**	Salvianolic acid B<br>$C_{36}H_{30}O_{16}$<br>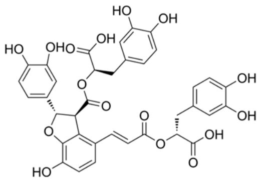	AD	Alleviates Aβ42 toxicity in *Drosophila*; transcriptomic analysis reveals modulation of multiple stress-response pathways[226].
**Phenolic acids**	Gallic acid<br>$C_7H_6O_5$<br>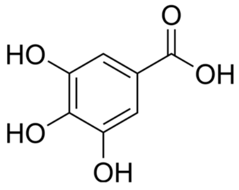 	AD	Dietary polyphenol; modulates cholinesterase activity in *Drosophila* with structure-dependent inhibitory profile and hormetic dose responses[227].
**Phenolic acids**	Ethyl caffeate<br>$C_{11}H_{12}O_4$	AD	Ameliorates Aβ42-induced rough-eye phenotype, prolongs lifespan in

	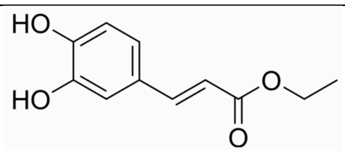		*Drosophila* Aβ42 models, and attenuates Aβ42-induced PC12 cell death[220].
**Phenolic acids**	Ellagic acid<br>$C_{14}H_6O_8$<br>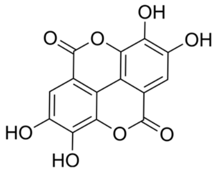	PD	Broad neuroprotection in transgenic and toxin-induced PD models: ROS scavenging, mitochondrial function maintenance, autophagic flux regulation, and inhibition of neuronal apoptosis[239].
**Terpenoids**	Safranal<br>$C_{10}H_{14}O$<br>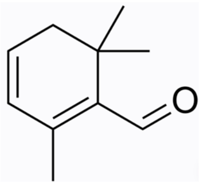	IBD	Dual inhibitor: suppresses IMD/NF-κB pathway AND activates Nrf2 antioxidant pathway in *Drosophila* IBD models[30].
**Terpenoids**	Perillartine<br>$C_{10}H_{15}NO$<br>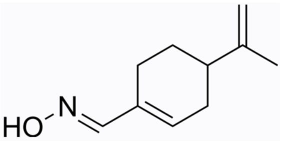	Diabetes	Natural terpenoid sweetener ~2000× sweeter than sucrose; validated in vivo for alleviating HFD-induced lipid metabolism disorders in broiler chickens. Whether it regulates gut microbiota and metabolic signaling in *Drosophila* diabetic models remains unexplored — a promising research gap[99].
**Terpenoids**	Thymoquinone<br>$C_{10}H_{12}O_2$<br>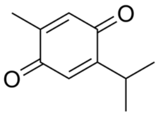	IBD	Downregulates pro-apoptotic genes, mitigates chemotherapy (Ara-C)-induced intestinal barrier damage in *Drosophila*[156].
**Terpenoids**	Physalin A<br>$C_{28}H_{30}O_{10}$	Cancer	Blocks upstream Grb2/RAS node of MAPK signaling; suppresses PI3K/Akt, MMP/uPA, and JNK/p38/AP1 pathways; dose-dependently inhibits tumor formation

	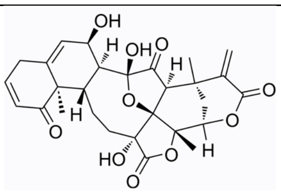		and distant metastasis in RasV12/$scrib^{-/-}$ *Drosophila*[185].
**Triterpenes**	Ursolic acid<br>$C_{30}H_{48}O_3$<br>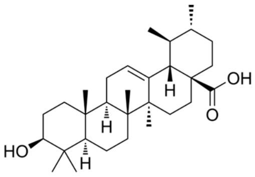	IBD	Inhibits NF-κB signaling and normalizes ISC proliferation in *Drosophila* IBD models[30].
**Terpenoids**	Crocetin<br>$C_{20}H_{24}O_4$<br>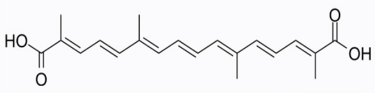	PD	Broad neuroprotection: ROS scavenging, mitochondrial function maintenance, autophagic flux regulation in PD fly models[218,236-233,238,239].
**Coumarins**	Psoralen<br>$C_{11}H_6O_3$<br>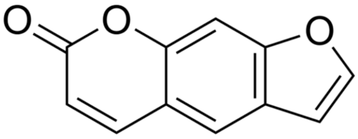	Cancer	Reported as an EGFR-targeting compound (from Psoralea Fructus); *Drosophila* model verification and RET-related validation remain absent — identified as a research gap for dual EGFR/RET screening in flies[191].
**Lignans**	Sesamin<br>$C_{20}H_{18}O_6$<br>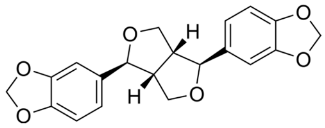	Diabetes	Upregulates SOD and catalase, reduces cardiac ROS, and ameliorates diabetic cardiomyopathy in *Drosophila* HSD models via Nrf2/CncC pathway[104].
**Lignans**	Magnolol<br>$C_{18}H_{18}O_2$<br>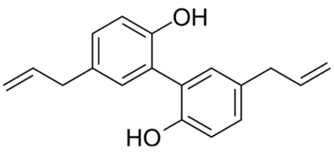	IBD	Active component of Danggui Buxue Decoction; co-suppresses Toll and IMD innate immune pathways to relieve intestinal inflammation[147].

**Stilbenoids**	Resveratrol<br>$C_{14}H_{12}O_3$<br>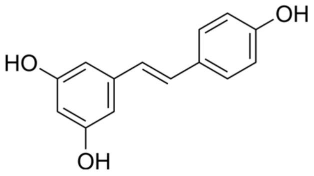	IBD, Cancer, AD, PD	IBD: co-suppresses Toll and IMD pathways; Cancer: targets glycolytic regulators HK II, PKM2 → suppresses Warburg effect in mammalian models; AD/PD: lowers oxidative stress, restores TH and SOD1, enhances L-DOPA efficacy while reducing side effects; Epigenetic: influences DNA methylation, histone acetylation in oncogenesis[147,194-197].
**Diarylheptanoids**	Curcumin<br>$C_{21}H_{20}O_6$<br>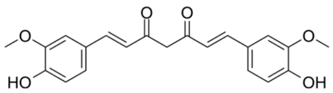	IBD, Cancer, AD	IBD: downregulates pro-apoptotic genes, mitigates chemotherapy-induced intestinal barrier damage; restructures gut microbiota; Cancer: targets glycolysis; epigenetic modulation (DNA methylation, histone acetylation, non-coding RNA); AD: NF-κB, JNK/JAK/STAT, Nrf2/Keap1 modulation[155,157].
**Phenolic alcohols**	3-Hydroxytyrosol<br>$C_8H_{10}O_3$<br>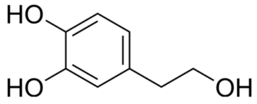	AD	Dietary phenolic compound; modulates cholinesterase activity in *Drosophila* with structure-dependent inhibitory profile and hormetic dose responses[227].
**Quinones (Naphthoquinones)**	Vitamin K2<br>$C_{41}H_{64}O_2$<br>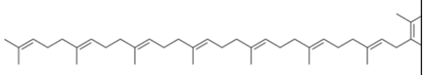	AD	Attenuates Aβ42 neurotoxicity by activating autophagy (upregulating Atg8a and Atg6a) and improving mitochondrial respiratory function.
**Polyphenols (Sesquiterpene dimer)**	Gossypol<br>$C_{30}H_{30}O_8$	Cancer	Targets key glycolytic regulators (HK II, PKM2) in mammalian cancer models to suppress Warburg effect. *Drosophila*-specific data pending[228].

	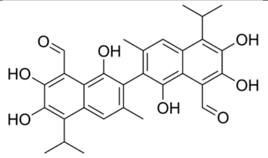		
**Glycoside**	Arbutin<br>$C_{12}H_{16}O_7$<br>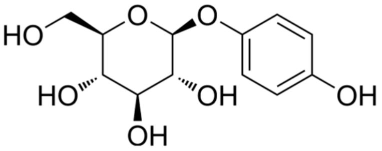	Nephrolithiasis	Chelates both Ca^{2+} and oxalate; at 1 mM nearly abolishes CaOx crystallization and disrupts crystal morphology; oxalate-binding activity is pH-independent, overcoming the pH limitation of potassium citrate[123].
**Flavonols**	Silibinin<br>$C_{25}H_{22}O_{10}$<br>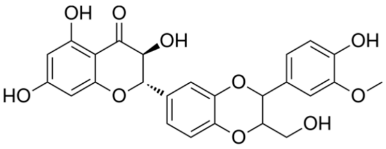	IBD	Inhibits JNK-mediated inflammation, oxidative stress, and epithelial apoptosis in *Drosophila* IBD models; protects intestinal barrier function[153,154].

Table S16. Comparison of the Cytochrome P450 (CYP) Drug-Metabolism System Between *Drosophila melanogaster* and Humans

Feature	*Drosophila melanogaster*	Humans (Homo sapiens)	Translational Implication
**Total number of CYP genes**	~85–90 functional CYP genes; one of the largest insect CYP complements [250,251].	57 functional CYP genes; 58 pseudogenes [252,253].	Despite having more CYP genes, the fly CYP repertoire is evolutionarily specialized for insect-specific functions (ecdysone metabolism, cuticular hydrocarbon synthesis, insecticide resistance), NOT for processing human-like pharmaceuticals [250,251].

**Major xenobiotic-metabolizing CYP families**	CYP4, CYP6, CYP9, CYP12, CYP28, CYP301–318 (insect-specific clans); CYP6 and CYP12 families dominate xenobiotic responses [250,251].	CYP1, CYP2, CYP3, CYP4 families; CYP3A4/5 alone metabolize ~50% of clinical drugs [252,253].	The major human drug-metabolizing families (CYP1–3) belong to clans that diverged from insect CYP lineages >500 million years ago. *Drosophila* has NO orthologs of these families [250,2252].
**Direct orthologs of key human drug-metabolizing CYPs**	NONE for CYP3A4, CYP2D6, CYP2C9, CYP2C19, CYP1A2. The closest insect P450s (CYP6 family) are functionally analogous but NOT orthologous [250,251].	CYP3A4, CYP2D6, CYP2C9, CYP2C19, CYP1A2, CYP2E1 — the major Phase I drug-metabolizing isoforms[248,249].	CRITICAL GAP: The absence of direct CYP3A4/2D6/2C9 orthologs means *Drosophila* cannot recapitulate human-like Phase I metabolite profiles for most pharmaceuticals. This is the single most important reason why drug metabolic fate in flies does not predict human pharmacokinetics[250,252,253].
**Characterized xenobiotic-metabolizing CYP enzymes**	Cyp6g1 (DDT/neonicotinoid resistance [255,257]), Cyp6a2 (broad-spectrum xenobiotic metabolism[251]), Cyp6a8, Cyp6d2, Cyp12d1, Cyp4p1, Cyp4e2[250,251].	CYP3A4, CYP2D6, CYP2C9, CYP2C19, CYP1A2, CYP2E1, CYP2C8, CYP2B6 [252,253].	*Drosophila* CYPs are well-characterized for insecticide metabolism but poorly characterized for natural-product/drug biotransformation. In contrast, human CYP substrate profiles are extensively documented[252,253].
**Substrate specificity &**	Broad substrate range shaped by	Broad, overlapping, well-characterized:	Even when a *Drosophila* CYP can metabolize a given drug,

**catalytic diversity**	ecological exposure (plant allelochemicals, environmental toxins); generally lower catalytic efficiency toward human drugs; some Cyp6 family members show remarkable substrate promiscuity.	CYP3A4 alone metabolizes substrates spanning >30 chemical classes; clinical drug–drug interactions (DDIs) are extensively documented	the resulting metabolites, kinetics (Km, Vmax), and regioselectivity differ from human CYPs, potentially leading to false-positive efficacy or false-negative toxicity results [1, 12].
**Regulation & inducibility**	Constitutively expressed + highly inducible via xenobiotic sensors; induction primarily through insect-specific nuclear receptors, e.g., DHR96 (a CAR/PXR-like receptor, but structurally distinct); no direct PXR or AhR orthologs [1, 11,57,61].	Inducible via PXR (CYP3A), CAR (CYP2B), AhR (CYP1A1/1A2), PPARα; CYP induction is a major source of clinical drug–drug interactions[252,253].	Since the regulatory machinery (nuclear receptors) differs fundamentally, CYP induction/repression data from flies cannot be extrapolated to predict human drug–drug interactions [250,252,256].
**Phase II conjugating enzymes**	Present but with narrower isoform diversity: ~35–40 GSTs, ~30 UGTs, limited SULTs;	Extensive UGT (UGT1A, UGT2B), GST (GSTA, GSTM, GSTP, GSTT), SULT, NAT, and	Phase II metabolism in *Drosophila* is less comprehensively characterized than in humans. Cumulative Phase I

	functional overlap with mammalian Phase II but different substrate preferences; UGT family expansion is less extensive than in mammals [250,256].	methyltransferase families; critical for drug clearance and detoxification [250].	+ Phase II differences mean that both the primary metabolite profile AND the clearance rate of a test compound in flies diverge substantially from the human situation[250,254,256].
**Overall drug metabolic clearance**	Limited ability to recapitulate human-like drug clearance rates; metabolic half-life in flies rarely correlates with human clearance [254].	Well-predicted by human liver microsome/hepatocyte assays and PBPK modeling [253].	Compounds that appear effective in flies may do so because of slower metabolic inactivation (false positives), or conversely, may be rapidly cleared by insect-specific CYPs (false negatives). This is a major translational gap [254].
**Strategies to bridge the translational gap**	① Humanized *Drosophila*: transgenic flies expressing human CYP3A4, CYP2D6, etc. in metabolic tissues (fat body, midgut) to partially recapitulate human-like metabolite profiles [254]; ② Co-testing with human liver microsomes or	Gold-standard: human hepatocyte assays, recombinant CYP panels, CYP-transgenic mouse models, clinical DDI studies [252,253].	Combining *Drosophila* phenotypic screening (for bioactivity) with orthogonal human CYP metabolism assays (for pharmacokinetic relevance) provides the most practical workflow. Humanized CYP-transgenic fly lines can reduce—but not eliminate—the metabolic translational gap [254]. (See main text Section 5, Lines 976–983.)

HepG2 cells for metabolite identification ; ③ Complementary validation in human CYP-transgenic mice [252].

Table S17. Comparison of Blood–Brain Barrier (BBB) Structure and Permeability Between *Drosophila melanogaster* and Humans

Feature	*Drosophila melanogaster*	Humans (Homo sapiens)	Translational Implication
**Anatomical location & cell type**	Formed by subperineurial glia (SPG) and perineurial glia that ensheath the entire CNS; a glial-based barrier fundamentally different from the vertebrate endothelial BBB [258,259]	Brain microvascular endothelial cells (BMECs) lining cerebral capillaries, supported by pericytes and astrocytic end-feet[260].	The different cellular origin (glial vs. endothelial) means BBB transport machinery, receptor expression, and regulation differ at the most basic level [260].
**Paracellular barrier — junction type**	Septate junctions (SJs) between subperineurial glial cells form the paracellular diffusion barrier. SJs are functionally analogous to vertebrate tight junctions but are	Tight junctions (TJs) between endothelial cells: claudins, occludin, and JAMs seal the paracellular space; astrocyte end-feet provide additional barrier support [258].	Septate and tight junctions share the same barrier FUNCTION but use different protein families. This means paracellular permeability to a given drug cannot be directly extrapolated

	molecularly distinct [259].		between species [258,259].
**Key junctional/barrier proteins**	Septate junction structural proteins: Neurexin IV (NrxIV, Caspr/paranodin homolog), Contactin, Neuroglian (NF155 homolog), Coracle (band 4.1 homolog); Claudin-like SJ proteins: Megatrachea (Mega), Sinuous (Sinu), Kune-kune (Kune); GPCR regulators of SJ formation: Moody, Loco (RGS protein)[258,259,261].	Tight junction proteins: Claudin-5 (endothelial-specific), Claudin-3, Occludin, ZO-1 (TJP1), ZO-2, ZO-3, JAM-A/B/C, ESAM, Tricellulin [259].	Although core protein families differ (claudins vs. claudin-like SJ proteins), the general molecular logic—transmembrane adhesion proteins + cytoskeletal adaptors + signaling regulators—is conserved. Claudin-5-like functional equivalents (Mega, Sinu) exist in flies [260,261].
**Efflux transporters at the BBB**	ABC transporters: Mdr65 and Mdr49 (P-glycoprotein/P-gp orthologs), Mdr50; additional ABC-B and ABC-G family transporters at glial membranes; OATPs also present[259-261]	P-glycoprotein (ABCB1/MDR1), BCRP (ABCG2), MRP family (ABCC1/4/5); the major "gate-keepers" limiting brain penetration of lipophilic drugs [258].	P-gp orthologs exist in *Drosophila* (Mdr65 is the functional counterpart of human MDR1), so efflux-based drug exclusion is partially conserved. However, substrate specificity of fly vs. human P-gp differs, and BCRP/MRP contributions are less well characterized in flies [260].

**Influx transporters & nutrient carriers**	Solute carriers (SLC) present: glucose transporters, amino acid transporters, monocarboxylate transporters (MCTs); overall transporter diversity is less extensively mapped than in humans [261].	GLUT1 (SLC2A1), LAT1 (SLC7A5), MCT1 (SLC16A1), OATP1A2, ENT1; well-characterized transporter expression and substrate profiles [260].	Differences in carrier expression and substrate affinity mean CNS bioavailability of a drug cannot be inferred from *Drosophila* brain exposure data. A drug crossing the *Drosophila* BBB may still be excluded by human BBB transporters [258,260,261].
**Relative permeability to small molecules**	The *Drosophila* BBB forms an effective, selective barrier with size- and charge-dependent permeability characteristics. While some small lipophilic molecules penetrate, the barrier actively excludes many xenobiotics. Direct quantitative permeability comparisons with the human BBB are sparse [258,259].	Well-characterized permeability profiles; Lipinski's "rule of 5" and CNS MPO scores provide predictive frameworks; extensive in vitro (PAMPA-BBB, hCMEC/D3 monolayers) and in vivo (rodent brain/plasma ratio, Kp,uu) data available [258].	The *Drosophila* BBB is a real, functional barrier—it is NOT simply "leaky." However, quantitative permeability rank-ordering between fly and human BBB for a given compound is rarely concordant. Fly CNS exposure data should be treated as qualitative screening readouts, not quantitative predictors of human brain penetration [258].

**Conservation of BBB signaling pathways**	Partly conserved: key developmental pathways (GPCR signaling via Moody/Loco, integrin-mediated adhesion, conserved polarity complexes) govern SJ formation; however, inflammatory mediators (cytokines, MMPs) that regulate human BBB permeability in disease have limited functional equivalents in flies[258].	Highly studied: Wnt/β-catenin, Hedgehog, VEGF, inflammatory cytokines (TNF-α, IL-1β, IL-6), MMP-2/9 signaling regulate TJ integrity and BBB permeability in health and disease [258]	Developmental genetic programs of BBB formation show meaningful conservation, supporting *Drosophila* as a model for BBB development. However, disease-relevant BBB disruption mechanisms (neuroinflammation, ischemia-reperfusion) involve pathways with less conservation [258].
**Predictive value for CNS drug screening**	Useful for: (a) high-throughput phenotypic CNS screening of NPs; (b) identifying neuroactive compounds in whole-organism behavioral assays; (c) genetic dissection of conserved BBB development genes NOT suitable for: predicting human BBB penetration, CNS pharmacokinetics, or	The gold standard for CNS drug development: human in vitro BBB models, rodent brain/plasma ratio (Kp,uu), CSF sampling, and clinical PET imaging [258].	*Drosophila* CNS screening should be positioned as an early-discovery triage step (does the compound show neuroactivity in vivo?), followed by orthogonal mammalian BBB permeability assays before lead optimization. Direct fly-to-human extrapolation of CNS exposure is not

	brain free fraction for lead optimization.		supported by current evidence [258,261].

References

1. Li, J.; Pandian, V.; Davidson, P.M.; Song, Y.; Chen, N.; Fong, D.Y.T. Burden and attributable risk factors of non-communicable diseases and subtypes in 204 countries and territories, 1990–2021: A systematic analysis for the global burden of disease study 2021. *Int. J. Surg.* **2025**, *111*, 2385–2397. https://doi.org/10.1097/JS9.0000000000002260.
2. Hu, W.; Gu, M.; Li, H.; Li, H.; Kan, J.; Zhang, L.; Ding, Q.; Zhu, Y.Z. Emerging natural products against obesity and metabolic dysfunction-associated steatotic liver disease/metabolic dysfunction-associated steatohepatitis: Direct target discovery and mechanistic insights. *Pharmacol. Rev.* **2026**, *78*, 100125. https://doi.org/10.1016/j.pharmr.2026.100125.
3. Cai, Z.; Wang, S.; Li, J. Treatment of Inflammatory Bowel Disease: A Comprehensive Review. *Front. Med.* **2021**, *8*, 2681. https://doi.org/10.3389/fmed.2021.765474.
4. Mahore, A.; Kamboj, P.; Kaleem, M.; Amir, M. Therapeutic management of arthritis: A review on structural and target-based approaches. *Arch. Der Pharm.* **2022**, *355*, 2200182. https://doi.org/10.1002/ardp.202200182.
5. López-Otín, C.; Blasco, M.A.; Partridge, L.; Serrano, M.; Kroemer, G. Hallmarks of aging: An expanding universe. *Cell* **2023**, *186*, 243–278. https://doi.org/10.1016/j.cell.2022.11.001.
6. Furman, D.; Campisi, J.; Verdin, E.; Carrera-Bastos, P.; Targ, S.; Franceschi, C.; Ferrucci, L.; Gilroy, D.W.; Fasano, A.; Miller, G.W.; et al. Chronic inflammation in the etiology of disease across the life span. *Nat. Med.* **2019**, *25*, 1822–1832. https://doi.org/10.1038/s41591-019-0675-0.
7. Tsurumi, A.; Li, W. Aging mechanisms—A perspective mostly from Drosophila. *Genet. Genom. Next* **2020**, *1*, e10026. https://doi.org/10.1002/ggn2.10026.
8. Latif, R.; Nawaz, T. Medicinal plants and human health: A comprehensive review of bioactive compounds, therapeutic effects, and applications. *Phytochem. Rev.* **2026**, *25*, 2299–2342. https://doi.org/10.1007/s11101-025-10194-7.
9. Scannell, J.W.; Bosley, J. When Quality Beats Quantity: Decision Theory, Drug Discovery, and the Reproducibility Crisis. *PLoS ONE* **2016**, *11*, e0147215. https://doi.org/10.1371/journal.pone.0147215.
10. Pandey, U.B.; Nichols, C.D. Human disease models in Drosophila melanogaster and the role of the fly in therapeutic drug discovery. *Pharmacol. Rev.* **2011**, *63*, 411–436. https://doi.org/10.1124/pr.110.003293.
11. Panossian, A. Challenges in phytotherapy research. *Front. Pharmacol.* **2023**, *14*, 1199516. https://doi.org/10.3389/fphar.2023.1199516.
12. Atanasov, A.G.; Zotchev, S.B.; Dirsch, V.M.; Orhan, I.E.; Banach, M.; Rollinger, J.M.; Barreca, D.; Weckwerth, W.; Bauer, R.; Bayer, E.A.; et al. Natural products in drug discovery: Advances and opportunities. *Nat. Rev. Drug Discov.* **2021**, *20*, 200–216. https://doi.org/10.1038/s41573-020-00114-z.
13. Ye, H.; Tao, X.; Zhang, W.; Chen, Y.; Yu, Q.; Xie, J. Food-derived bioactive peptides: Production, biological activities, opportunities and challenges. *J. Future Foods* **2022**, *2*, 294–306. https://doi.org/10.1016/j.jfutfo.2022.08.002.
14. Gonçalves, A.C.; Pinto, A.R.; Cima, A.; Olo-Fontinha, E.; Martins, J.C.L.; Garcia, J.; Lemos, A.; Saavedra, M.J.; Pintado, M.M.; Alves, M.J. Plant-Derived Bioactive Compounds: One Health Perspective. *Appl. Sci.* **2026**, *16*, 327.
15. Lin, S.; Li, L.; Zhuo, S.; Yu, L.; Huang, Y.; Chen, X.; Ti, H. Multi-pathway targeting by natural products in chronic respiratory diseases: Opportunities, challenges, and future perspectives. *Chin. Herbal. Med.* **2026**, *18*, 638–642. https://doi.org/10.1016/j.chmed.2026.05.003.

16. Morimoto, J.; Pietras, Z. Natural history of model organisms: The secret (group) life of Drosophila melanogaster larvae and why it matters to developmental ecology. *Ecol. Evol.* **2020**, *10*, 13593–13601. https://doi.org/10.1002/ece3.7003.
17. O'Grady, P.; Desalle, R. Phylogeny of the Genus Drosophila. *Genetics* **2018**, *209*, 1–25. https://doi.org/10.1534/genetics.117.300583.
18. Reiter, L.T.; Potocki, L.; Chien, S.; Gribskov, M.; Bier, E. A systematic analysis of human disease-associated gene sequences in Drosophila melanogaster. *Genome Res.* **2001**, *11*, 1114–1125. https://doi.org/10.1101/gr.169101.
19. Ugur, B.; Chen, K.; Bellen, H.J. Drosophila tools and assays for the study of human diseases. *Dis. Model. Mech.* **2016**, *9*, 235–244. https://doi.org/10.1242/dmm.023762.
20. Bellen, H.J.; Tong, C.; Tsuda, H. 100 years of Drosophila research and its impact on vertebrate neuroscience: A history lesson for the future. *Nat. Rev. Neurosci.* **2010**, *11*, 514–522. https://doi.org/10.1038/nrn2839.
21. Moraes, K.C.M.; Montagne, J. Drosophila melanogaster: A Powerful Tiny Animal Model for the Study of Metabolic Hepatic Diseases. *Front. Physiol.* **2021**, *12*, 728407. https://doi.org/10.3389/fphys.2021.728407.
22. Chatterjee, N.; Perrimon, N. What fuels the fly: Energy metabolism in Drosophila and its application to the study of obesity and diabetes. *Sci. Adv.* **2021**, *7*, eabg4336. https://doi.org/10.1126/sciadv.abg4336.
23. Ren, X.; Holsteens, K.; Li, H.; Sun, J.; Zhang, Y.; Liu, L.P.; Liu, Q.; Ni, J.Q. Genome editing in Drosophila melanogaster: From basic genome engineering to the multipurpose CRISPR-Cas9 system. *Sci. China Life Sci.* **2017**, *60*, 476–489. https://doi.org/10.1007/s11427-017-9029-9.
24. Lopez-Ortiz, C.; Gracia-Rodriguez, C.; Belcher, S.; Flores-Iga, G.; Das, A.; Nimmakayala, P.; Balagurusamy, N.; Reddy, U.K. Drosophila melanogaster as a Translational Model System to Explore the Impact of Phytochemicals on Human Health. *Int. J. Mol. Sci.* **2023**, *24*, 13365.
25. Mudjahid, M.; Latada, N.P.; Ophinni, Y.; Nainu, F. Drosophila-based screening of herbal compounds targeting FOXO signaling pathway in type 2 diabetes mellitus. *Front. Pharmacol.* **2025**, *16*, 1621414. https://doi.org/10.3389/fphar.2025.1621414.
26. Sheng, X.; He, J.; Zhang, L. Unlocking nature's medicine cabinet through Drosophila-Driven exploration. *Phytomedicine* **2025**, *146*, 157123. https://doi.org/10.1016/j.phymed.2025.157123.
27. Victor Atoki, A.; Aja, P.M.; Shinkafi, T.S.; Ondari, E.N.; Adeniyi, A.I.; Fasogbon, I.V.; Dangana, R.S.; Shehu, U.U.; Akin-Adewumi, A. Exploring the versatility of Drosophila melanogaster as a model organism in biomedical research: A comprehensive review. *Fly* **2025**, *19*, 2420453. https://doi.org/10.1080/19336934.2024.2420453.
28. Li, X.; Sun, S.; Liu, X.; Meng, Q.; Tian, M.; Li, J.; Ren, S.; Huang, Z.; Wang, Y.; Du, S. Drosophila melanogaster models for investigating inflammatory bowel disease: Methods, pathology, mechanisms, and therapeutic approaches. *Biomol. Biomed.* **2025**, *26*, 186–199. https://doi.org/10.17305/bb.2025.12656.
29. Mohamed, S.A.; Badr, O.A. Modeling human neurodegenerative disorders in Drosophila: Strategies and translational opportunities. *Mol. Biol. Rep.* **2026**, *53*, 745. https://doi.org/10.1007/s11033-026-11844-5.
30. Xiu, M.; Wang, Y.; Yang, D.; Zhang, X.; Dai, Y.; Liu, Y.; Lin, X.; Li, B.; He, J. Using Drosophila melanogaster as a suitable platform for drug discovery from natural products in inflammatory bowel disease. *Front. Pharmacol.* **2022**, *13*, 1072715. https://doi.org/10.3389/fphar.2022.1072715.
31. Maitra, U.; Ciesla, L. Using Drosophila as a platform for drug discovery from natural products in Parkinson's disease. *Medchemcomm* **2019**, *10*, 867–879. https://doi.org/10.1039/c9md00099b.
32. Nathan, D.M. The diabetes control and complications trial/epidemiology of diabetes interventions and complications study at 30 years: Overview. *Diabetes Care* **2014**, *37*, 9–16. https://doi.org/10.2337/dc13-2112.

33. Einarson, T.R.; Acs, A.; Ludwig, C.; Panton, U.H. Prevalence of cardiovascular disease in type 2 diabetes: A systematic literature review of scientific evidence from across the world in 2007-2017. *Cardiovasc. Diabetol.* **2018**, *17*, 83. https://doi.org/10.1186/s12933-018-0728-6.
34. Miloudi, K.; Oubaha, M.; Ménard, C.; Dejda, A.; Guber, V.; Cagnone, G.; Wilson, A.M.; Tétreault, N.; Mawambo, G.; Binet, F.; et al. NOTCH1 signaling induces pathological vascular permeability in diabetic retinopathy. *Proc. Natl. Acad. Sci. USA* **2019**, *116*, 4538–4547. https://doi.org/10.1073/pnas.1814711116.
35. Zhao, Q.; Liu, J.; Deng, H.; Ma, R.; Liao, J.-Y.; Liang, H.; Hu, J.; Li, J.; Guo, Z.; Cai, J.; et al. Targeting Mitochondria-Located circRNA SCAR Alleviates NASH via Reducing mROS Output. *Cell* **2020**, *183*, 76–93. https://doi.org/10.1016/j.cell.2020.08.009.
36. Zhao, P.; Sun, X.; Chaggan, C.; Liao, Z.; In Wong, K.; He, F.; Singh, S.; Loomba, R.; Karin, M.; Witztum, J.L.; et al. An AMPK-caspase-6 axis controls liver damage in nonalcoholic steatohepatitis. *Science* **2020**, *367*, 652–660. https://doi.org/10.1126/science.aay0542.
37. Demir, S.; Nawroth, P.P.; Herzig, S.; Ekim Üstünel, B. Emerging Targets in Type 2 Diabetes and Diabetic Complications. *Adv. Sci.* **2021**, *8*, e2100275. https://doi.org/10.1002/advs.202100275.
38. Nathan, D.M.; Genuth, S.; Lachin, J.; Cleary, P.; Crofford, O.; Davis, M.; Rand, L.; Siebert, C. The effect of intensive treatment of diabetes on the development and progression of long-term complications in insulin-dependent diabetes mellitus. *N. Engl. J. Med.* **1993**, *329*, 977–986.
39. Kalyani, R.R.; Neumiller, J.J.; Maruthur, N.M.; Wexler, D.J. Diagnosis and Treatment of Type 2 Diabetes in Adults: A Review. *JAMA* **2025**, *334*, 984–1002. https://doi.org/10.1001/jama.2025.5956.
40. Eizirik, D.L.; Pasquali, L.; Cnop, M. Pancreatic β-cells in type 1 and type 2 diabetes mellitus: Different pathways to failure. *Nat. Rev. Endocrinol.* **2020**, *16*, 349–362. https://doi.org/10.1038/s41574-020-0355-7.
41. Elkhalifa, A.M.E.; Nazar, M.; Ali, S.I.; Khursheed, I.; Taifa, S.; Ahmad Mir, M.; Shah, I.H.; Malik, M.; Ramzan, Z.; Ahad, S.; et al. Novel Therapeutic Agents for Management of Diabetes Mellitus: A Hope for Drug Designing against Diabetes Mellitus. *Life* **2024**, *14*, 99. https://doi.org/10.3390/life14010099.
42. Rulifson, E.J.; Kim, S.K.; Nusse, R. Ablation of insulin-producing neurons in flies: Growth and diabetic phenotypes. *Science* **2002**, *296*, 1118–1120.
43. Liguori, F.; Mascolo, E.; Vernì, F. The Genetics of Diabetes: What We Can Learn from Drosophila. *Int. J. Mol. Sci.* **2021**, *22*, 11295. https://doi.org/10.3390/ijms222011295.
44. Salgado-Canales, D.; Quenti, D.; Lourido, F.; Cifuentes, M.; Tobar, N. "Effect of time-restricted feeding on high-fat diet-induced metabolic dysfunction in Drosophila melanogaster". *Biochim. Biophys. Acta Mol. Basis Dis.* **2023**, *1869*, 166749. https://doi.org/10.1016/j.bbadis.2023.166749.
45. Bisen, R.S.; Iqbal, F.M.; Cascino-Milani, F.; Bockemühl, T.; Ache, J.M. Nutritional state-dependent modulation of insulin-producing cells in Drosophila. *eLife* **2025**, *13*, RP98514. https://doi.org/10.7554/eLife.98514.
46. Baenas, N.; Wagner, A.E. Drosophila melanogaster as a Model Organism for Obesity and Type-2 Diabetes Mellitus by Applying High-Sugar and High-Fat Diets. *Biomolecules* **2022**, *12*, 307. https://doi.org/10.3390/biom12020307.
47. Hong, S.-H.; Kang, M.; Lee, K.-S.; Yu, K. High fat diet-induced TGF-β/Gbb signaling provokes insulin resistance through the tribbles expression. *Sci. Rep.* **2016**, *6*, 30265. https://doi.org/10.1038/srep30265.
48. Singh, R.; Gholipourmalekabadi, M.; Shafikhani, S.H. Animal models for type 1 and type 2 diabetes: Advantages and limitations. *Front. Endocrinol.* **2024**, *15*, 1359685. https://doi.org/10.3389/fendo.2024.1359685.
49. Fang, J.Y.; Lin, C.H.; Huang, T.H.; Chuang, S.Y. In Vivo Rodent Models of Type 2 Diabetes and Their Usefulness for Evaluating Flavonoid Bioactivity. *Nutrients* **2019**, *11*, 530. https://doi.org/10.3390/nu11030530.

50. Adams, M.D.; Celniker, S.E.; Holt, R.A.; Evans, C.A.; Gocayne, J.D.; Amanatides, P.G.; Scherer, S.E.; Li, P.W.; Hoskins, R.A.; Galle, R.F.; et al. The Genome Sequence of Drosophila melanogaster. Science 2000, 287, 2185–2195. https://doi.org/10.1126/science.287.5461.2185.
51. Nässel, D.R.; Liu, Y.; Luo, J. Insulin/IGF signaling and its regulation in Drosophila. *Gen. Comp. Endocrinol.* **2015**, *221*, 255–266. https://doi.org/10.1016/j.ygcen.2014.11.021.
52. Skowronek, P.; Wójcik, Ł.; Strachecka, A. Fat Body—Multifunctional Insect Tissue. *Insects* **2021**, *12*, 547. https://doi.org/10.3390/insects12060547.
53. Zeng, Y.; Yang, Y.; Zhu, Y.; Xu, P.; Cheong, C.; Zhao, Y.J.; Baeg, G.H. Altered mitochondrial functionality in metabolic disorders: Insights from Drosophila studies. *Front. Endocrinol.* **2026**, *17*, 1817121. https://doi.org/10.3389/fendo.2026.1817121.
54. Hoffmann, J.; Romey, R.; Fink, C.; Roeder, T. Drosophila as a Model to Study Metabolic Disorders. In *Yellow Biotechnology I: Insect Biotechnologie in Drug Discovery and Preclinical Research*; Vilcinskas, A., Ed.; Springer: Berlin/Heidelberg, Germany, 2013; pp. 41–61. https://doi.org/10.1007/10_2013_196.
55. Semaniuk, U.; Piskovatska, V.; Strilbytska, O.; Strutynska, T.; Burdyliuk, N.; Vaiserman, A.; Bubalo, V.; Storey, K.B.; Lushchak, O. Drosophila insulin-like peptides: From expression to functions—A review. *Entomol. Exp. Appl.* **2021**, *169*, 195–208. https://doi.org/10.1111/eea.12981.
56. Birse, R.T.; Choi, J.; Reardon, K.; Rodriguez, J.; Graham, S.; Diop, S.; Ocorr, K.; Bodmer, R.; Oldham, S. High-Fat-Diet-Induced Obesity and Heart Dysfunction Are Regulated by the TOR Pathway in *Drosophila*. *Cell Metab.* **2010**, *12*, 533–544. https://doi.org/10.1016/j.cmet.2010.09.014.
57. Liu, Z.; Huang, X. Lipid metabolism in Drosophila: Development and disease. *Acta Biochim. Biophys. Sin.* **2013**, *45*, 44–50. https://doi.org/10.1093/abbs/gms105.
58. Cormier, R.P.J.; Champigny, C.M.; Simard, C.J.; St-Coeur, P.-D.; Pichaud, N. Dynamic mitochondrial responses to a high-fat diet in Drosophila melanogaster. *Sci. Rep.* **2019**, *9*, 4531. https://doi.org/10.1038/s41598-018-36060-5.
59. Phogat, A.; Singh, J.; Malik, V. Pharmacological effects of Berberine—A Chinese medicine, against xenobiotics toxicity. *Pharmacol. Res.-Mod. Chin. Med.* **2024**, *13*, 100507. https://doi.org/10.1016/j.prmcm.2024.100507.
60. Ruppert, P.M.M.; Kersten, S. Mechanisms of hepatic fatty acid oxidation and ketogenesis during fasting. *Trends Endocrinol. Metab.* **2024**, *35*, 107–124. https://doi.org/10.1016/j.tem.2023.10.002.
61. Yue, T.; Jiang, M.; Onuki, K.; Itoh, M.; Tonoki, A. High-fat diet impairs intermediate-term memory by autophagic-lysosomal dysfunction in Drosophila. *PLoS Genet.* **2025**, *21*, e1011818. https://doi.org/10.1371/journal.pgen.1011818.
62. Honegger, B.; Galic, M.; Köhler, K.; Wittwer, F.; Brogiolo, W.; Hafen, E.; Stocker, H. Imp-L2, a putative homolog of vertebrate IGF-binding protein 7, counteracts insulin signaling in Drosophila and is essential for starvation resistance. *J. Biol.* **2008**, *7*, 10. https://doi.org/10.1186/jbiol72.
63. Takeuchi, T.; Kubota, T.; Nakanishi, Y.; Tsugawa, H.; Suda, W.; Kwon, A.T.-J.; Yazaki, J.; Ikeda, K.; Nemoto, S.; Mochizuki, Y.; et al. Gut microbial carbohydrate metabolism contributes to insulin resistance. *Nature* **2023**, *621*, 389–395. https://doi.org/10.1038/s41586-023-06466-x.
64. Bevinakoppamath, S.; Srinivas, A.; Chandan Gowda, U.; Prashant, A.; Singh, A.; Rao, V.K. Understanding diabetes and its complications through the lens of Drosophila. *Exp. Mol. Pathol.* **2026**, *145*, 105017. https://doi.org/10.1016/j.yexmp.2025.105017.
65. Ikeya, T.; Galic, M.; Belawat, P.; Nairz, K.; Hafen, E. Nutrient-Dependent Expression of Insulin-like Peptides from Neuroendocrine Cells in the CNS Contributes to Growth Regulation in Drosophila. *Curr. Biol.* **2002**, *12*, 1293–1300. https://doi.org/10.1016/S0960-9822(02)01043-6.
66. Álvarez-Rendón, J.P.; Salceda, R.; Riesgo-Escovar, J.R. Drosophila melanogaster as a Model for Diabetes Type 2 Progression. *BioMed. Res. Int.* **2018**, *2018*, 1417528. https://doi.org/10.1155/2018/1417528.

67. Böhni, R.; Riesgo-Escovar, J.; Oldham, S.; Brogiolo, W.; Stocker, H.; Andruss, B.F.; Beckingham, K.; Hafen, E. Autonomous control of cell and organ size by CHICO, a Drosophila homolog of vertebrate IRS1-4. *Cell* **1999**, *97*, 865–875.
68. Zhang, R.-X.; Li, A.-Q.; Zhao, X.-Y.; Wang, B.; Yang, Z.-C.; Liu, Z.-Y.; Ji, C.; Shi, Y.-C.; Neely, G.G.; Wang, Q.-P. Identification of SEC16B as a novel regulator of glucose homeostasis. *Diabetologia* **2025**, *68*, 2247–2262. https://doi.org/10.1007/s00125-025-06501-8.
69. Li, Y.; Xu, Y.; Zhang, B.; Wang, Z.; Ma, L.; Sun, L.; Wang, X.; Lin, Y.; Li, J.-A.; Wu, C. Atractylodes macrocephala Koidz. and Cuscuta chinensis Lam. extract relieves insulin resistance via PI3K/Akt signalling in diabetic Drosophila. *J. Tradit. Complement. Med.* **2024**, *14*, 424–434. https://doi.org/10.1016/j.jtcme.2024.01.010.
70. Cao, X.; La, X.; Zhang, B.; Wang, Z.; Li, Y.; Bo, Y.; Chang, H.; Gao, X.; Tian, C.; Wu, C.; et al. Sanghuang Tongxie Formula Ameliorates Insulin Resistance in Drosophila Through Regulating PI3K/Akt Signaling. *Front. Pharmacol.* **2022**, *13*, 874180. https://doi.org/10.3389/fphar.2022.874180.
71. Saliu, J.A.; Olajuyin, A.M.; Akinnubi, A. Modulatory effect of Artocarpus camansi on ILP-2, InR, and Imp-L2 genes of sucrose–induced diabetes mellitus in Drosophila melanogaster. *Comp. Biochem. Physiol. Part C Toxicol. Pharmacol.* **2021**, *246*, 109041. https://doi.org/10.1016/j.cbpc.2021.109041.
72. Omoboyowa, D.A.; Agoi, M.D.; Shodehinde, S.A.; Saibu, O.A.; Saliu, J.A. Antidiabetes study of Spondias mombin (Linn) stem bark fractions in high-sucrose diet-induced diabetes in Drosophila melanogaster. *J. Taibah Univ. Med. Sci.* **2023**, *18*, 663–675. https://doi.org/10.1016/j.jtumed.2023.01.011.
73. Arjunan, S.; Thangaiyan, R.; Balaraman, D. Biochanin A, a soy isoflavone, diminishes insulin resistance by modulating insulin-signalling pathway in high-fat diet-induced diabetic mice. *Arch. Physiol. Biochem.* **2020**, *129*, 316–322. https://doi.org/10.1080/13813455.2020.1820525.
74. Zhang, N.; Zhang, W.; Guo, X.; Liu, J.; Li, S.; Zhang, H.; Fan, B. Genistein protects against hyperglycemia and fatty liver disease in diet-induced prediabetes mice via activating hepatic insulin signaling pathway. *Front. Nutr.* **2022**, *9*, 1072044. https://doi.org/10.3389/fnut.2022.1072044.
75. Yang, X.; Dai, K.; Wang, S. Genistein Enhances GLUT4 Expression and Translocation in the Gastrocnemius Muscle and Improves Systemic Glucose Metabolism in Ovariectomized Mice. *Nutrients* **2025**, *17*, 2811. https://doi.org/10.3390/nu17172811.
76. Lüersen, K.; Fischer, A.; Bauer, I.; Huebbe, P.; Uekaji, Y.; Chikamoto, K.; Nakata, D.; Hiramatsu, N.; Terao, K.; Rimbach, G. Soy Extract, Rich in Hydroxylated Isoflavones, Exhibits Antidiabetic Properties In Vitro and in Drosophila melanogaster In Vivo. *Nutrients* **2023**, *15*, 1392. https://doi.org/10.3390/nu15061392.
77. Yu, S.; Lei, X.; Meng, S.; Teng, X.; Yue, G.; Zhu, J.; Bi, B.; Jin, L.H. Crocin alleviates high-sugar diet-induced type 2 diabetes model in Drosophila. *Food Biosci.* **2025**, *71*, 107265. https://doi.org/10.1016/j.fbio.2025.107265.
78. Li, Y.; Wang, J.; Xu, Y.; Meng, Q.; Wu, M.; Su, Y.; Miao, Y.; Wang, Y. The water extract of Potentilla discolor Bunge (PDW) ameliorates high-sugar diet-induced type II diabetes model in Drosophila melanogaster via JAK/STAT signaling. *J. Ethnopharmacol.* **2023**, *316*, 116760. https://doi.org/10.1016/j.jep.2023.116760.
79. Li, Y.; Wu, F.; Zhang, J.; Xu, Y.; Chang, H.; Yu, Y.; Jiang, C.; Gao, X.; Liu, H.; Chen, Z.; et al. Mechanisms of Action of Potentilla discolor Bunge in Type 2 Diabetes Mellitus Based on Network Pharmacology and Experimental Verification in Drosophila. *Drug Des. Dev. Ther.* **2024**, *18*, 747–766. https://doi.org/10.2147/dddt.S439876.
80. Meng, Q.; Li, Y.; Xu, Y.; Wang, Y. Acetobacter and lactobacillus alleviate the symptom of insulin resistance by blocking the JNK-JAK/STAT pathway in Drosophila melanogaster. *Biochim. Biophys. Acta Mol. Basis Dis.* **2024**, *1870*, 166901. https://doi.org/10.1016/j.bbadis.2023.166901.
81. Ma, P.; Zhang, Y.; Yin, Y.; Wang, S.; Chen, S.; Liang, X.; Li, Z.; Deng, H. Gut microbiota metabolite tyramine ameliorates high-fat diet-induced insulin resistance via increased Ca(2+) signaling. *Embo J.* **2024**, *43*, 3466–3493. https://doi.org/10.1038/s44318-024-00162-w.

82. Xu, Z.; Feng, W.; Shen, Q.; Yu, N.; Yu, K.; Wang, S.; Chen, Z.; Shioda, S.; Guo, Y. Rhizoma Coptidis and Berberine as a Natural Drug to Combat Aging and Aging-Related Diseases via Anti-Oxidation and AMPK Activation. *Aging Dis.* **2017**, *8*, 760–777. https://doi.org/10.14336/AD.2016.0620.
83. Montaser, O.; El-Aasr, M.; Tawfik, H.O.; Meshrif, W.S.; Elbrense, H. Drosophila melanogaster as a model organism for diabetes II treatment by the ethyl acetate fraction of Atriplex halimus L. *J. Exp. Zool. A Ecol. Integr. Physiol.* **2024**, *341*, 702–716. https://doi.org/10.1002/jez.2812.
84. Xiao, S.; Peng, K.; Li, C.; Long, Y.; Yu, Q. The role of sphingosine-1-phosphate in autophagy and related disorders. *Cell Death Discov.* **2023**, *9*, 380. https://doi.org/10.1038/s41420-023-01681-x.
85. Shen, Y.; Zhao, S.; Wang, S.; Pan, X.; Zhang, Y.; Xu, J.; Jiang, Y.; Li, H.; Zhang, Q.; Gao, J.; et al. S1P/S1PR3 axis promotes aerobic glycolysis by YAP/c-MYC/PGAM1 axis in osteosarcoma. *EBioMedicine* **2019**, *40*, 210–223. https://doi.org/10.1016/j.ebiom.2018.12.038.
86. Park, W.-J.; Park, I.; Jung, Y.; Ahn, J.-H.; Shin, S.-H.; Kim, S.; Lee, E.-J.; Park, J.-W.; Kim, M.I.N. C16-ceramide and sphingosine 1-phosphate/S1PR2 have opposite effects on cell growth through mTOR signaling pathway regulation. *Oncol. Rep.* **2018**, *40*, 2977–2987. https://doi.org/10.3892/or.2018.6689.
87. Yu, G.; Sun, M.; Zhang, T.; Xu, H.; Wang, J.; Ye, W.; Wang, P.; Zhang, S.; Zhang, C.; Sun, Y. Lanhuashen stimulates the positive cross-regulation mediated by the S1P axis to ameliorate the disorder of glucolipid metabolism induced by the high sucrose diet in Drosophila melanogaster. *J. Ethnopharmacol.* **2024**, *319*, 117248. https://doi.org/10.1016/j.jep.2023.117248.
88. Barchetta, I.; Cimini, F.A.; Dule, S.; Cavallo, M.G. Dipeptidyl Peptidase 4 (DPP4) as A Novel Adipokine: Role in Metabolism and Fat Homeostasis. *Biomedicines* **2022**, *10*. 2306. https://doi.org/10.3390/biomedicines10092306.
89. Lagunas-Rangel, F.A.; Liao, S.; Williams, M.J.; Trukhan, V.; Fredriksson, R.; Schiöth, H.B. Drosophila as a Rapid Screening Model to Evaluate the Hypoglycemic Effects of Dipeptidyl Peptidase 4 (DPP4) Inhibitors: High Evolutionary Conservation of DPP4. *Biomedicines* **2023**, *11*, 3032. https://doi.org/10.3390/biomedicines11113032.
90. Chen, S.; Pan, H.; Huan, P.; Wang, S.; Sun, L.; Ning, D.; Ma, T.; Zhuang, Y. Exploration of novel dipeptidyl peptidase-IV inhibitory tetrapeptide from walnut (Juglans sigillata) protein and its effect on diabetes-like phenotypes induced by high-sugar diet in Drosophila melanogaster. *Food Res. Int.* **2025**, *211*, 116487. https://doi.org/10.1016/j.foodres.2025.116487.
91. Wang, M.; Mao, H.; Chen, J.; Qi, L.; Wang, J. Ameliorative effect of bayberry leaves proanthocyanidins on high sugar diet induced Drosophila melanogaster. *Front. Pharmacol.* **2022**, *13*, 1008580. https://doi.org/10.3389/fphar.2022.1008580.
92. Musselman, L.P.; Fink, J.L.; Narzinski, K.; Ramachandran, P.V.; Hathiramani, S.S.; Cagan, R.L.; Baranski, T.J. A high-sugar diet produces obesity and insulin resistance in wild-type Drosophila. *Dis. Model. Mech.* **2011**, *4*, 842–849. https://doi.org/10.1242/dmm.007948.
93. Nwanna, E.; Ojo, R.; Shafiq, N.; Ali, A.; Okello, E.; Oboh, G. An In Silico In Vitro and In Vivo Study on the Influence of an Eggplant Fruit (Solanum anguivi Lam) Diet on Metabolic Dysfunction in the Sucrose-Induced Diabetic-like Fruit Fly (Drosophila melanogaster). *Foods* **2024**, *13*, 559. https://doi.org/10.3390/foods13040559.
94. Oloyede, O.I.; Ibrahim, S.J.; Anadozie, S.O.; Aytar, E.C.; Akawa, O.B.; Adu, I.A.; Omodaratan, O.S.; Obafemi, O.T.; Adewale, O.B. In Silico evaluation of phytoconstituents from Carica Papaya and its anti-hyperglycemic activities on high sucrose-induced oxidative stress in Drosophila melanogaster. *Sci. Rep.* **2025**, *15*, 29834. https://doi.org/10.1038/s41598-025-13246-2.
95. Dechakhamphu, A.; Wongchum, N.; Chumroenphat, T.; Tanomtong, A.; Pinlaor, S.; Siriamornpun, S. In Vitro and In Vivo Evaluation for Antioxidant and Anti-Diabetic Properties of Cyperus rotundus L. Kombucha. *Foods* **2023**, *12*, 4059. https://doi.org/10.3390/foods12224059.
96. Dong, Z.; Sun, Y.; Wei, G.; Li, S.; Zhao, Z. Ergosterol Ameliorates Diabetic Nephropathy by Attenuating Mesangial Cell Proliferation and Extracellular Matrix Deposition via the TGF-β1/Smad2 Signaling Pathway. *Nutrients* **2019**, *11*, 483. https://doi.org/10.3390/nu11020483.

97. Wang, L.; Yang, Z.; Pang, Y.; Wu, X.; Zhang, X.; Zhu, T.; Ding, X.; Liu, W.; Zhou, Y.; Zhang, P.; et al. Gut Microbial Genetic Variation Prolongs Host Healthy Longevity and Remodels Metabolome and Proteome in Drosophila Melanogaster. *Adv. Sci.* **2025**, *12*, e05469. https://doi.org/10.1002/advs.202505469.
98. Youn, I.; Piao, D.; Park, J.; Ock, S.A.; Han, S.; Han, A.-R.; Shin, S.; Seo, E.K. Anti-Obesity Activities of the Compounds from Perilla frutescens var. acuta and Chemical Profiling of the Extract. *Molecules* **2024**, *29*, 2465.
99. Xiao, Y.; Jia, M.; Jiang, T.; Zhang, C.; Qi, X.; Sun, Y.; Gao, J.; Zhou, L.; Li, Y. Dietary supplementation with perillartine ameliorates lipid metabolism disorder induced by a high-fat diet in broiler chickens. *Biochem. Biophys. Res. Commun.* **2022**, *625*, 66–74. https://doi.org/10.1016/j.bbrc.2022.07.116.
100. Plaza-Diaz, J.; Pastor-Villaescusa, B.; Rueda-Robles, A.; Abadia-Molina, F.; Ruiz-Ojeda, F.J. Plausible Biological Interactions of Low- and Non-Calorie Sweeteners with the Intestinal Microbiota: An Update of Recent Studies. *Nutrients* **2020**, *12*, 1153.
101. Huang, J.H.; Douglas, A.E. Consumption of dietary sugar by gut bacteria determines Drosophila lipid content. *Biol. Lett.* **2015**, *11*, 20150469. https://doi.org/10.1098/rsbl.2015.0469.
102. Hubrecht, I.; Baenas, N.; Sina, C.; Wagner, A. Effects of non-caloric artificial sweeteners on naïve and dextran sodium sulfate-exposed Drosophila melanogaster. *Food Front.* **2022**, *3*, 728–735. https://doi.org/10.1002/fft2.147.
103. Catalani, E.; Del Quondam, S.; Brunetti, K.; Cherubini, A.; Bongiorni, S.; Taddei, A.R.; Zecchini, S.; Giovarelli, M.; De Palma, C.; Perrotta, C.; et al. Neuroprotective role of plumbagin on eye damage induced by high-sucrose diet in adult fruit fly Drosophila melanogaster. *Biomed. Pharmacother.* **2023**, *166*, 115298. https://doi.org/10.1016/j.biopha.2023.115298.
104. Ratnawati, R.; Aswad, M.; Jumriani, J.; Nurhidayah, A.; Azmin, M.R.; Filmaharani, F.; Roosevelt, A.; Hardiyanti, W.; Latada, N.P.; Mudjahid, M.; et al. In Silico and In Vivo Investigation of the Anti-Hyperglycemic Effects of Caffeic Acid. *ACS Omega* **2025**, *10*, 14052–14062. https://doi.org/10.1021/acsomega.4c11062.
105. Carocho, M.; Ferreira, I.C.F.R. A review on antioxidants, prooxidants and related controversy: Natural and synthetic compounds, screening and analysis methodologies and future perspectives. *Food Chem. Toxicol.* **2013**, *51*, 15–25. https://doi.org/10.1016/j.fct.2012.09.021.
106. Suchá, L.; Tomsik, P. The Steroidal Glycoalkaloids from Solanaceae: Toxic Effect, Antitumour Activity and Mechanism of Action. *Planta Medica* **2016**, *82*, 379–387. https://doi.org/10.1055/s-0042-100810.
107. Pitchakarn, P.; Inthachat, W.; Karinchai, J.; Temviriyanukul, P. Human Hazard Assessment Using Drosophila Wing Spot Test as an Alternative In Vivo Model for Genotoxicity Testing—A Review. *Int. J. Mol. Sci.* **2021**, *22*, 9932. https://doi.org/10.3390/ijms22189932.
108. Savić, A.; Šipovac, M.; Novaković, A.; Vasiljević, L.; Palangetić, M. Antioxidant enzymes, oxidative stress, and physiological aging markers in Drosophila melanogaster (Diptera: Drosophilidae): A systematic review with translational perspectives. *J. Insect Sci.* **2026**, *26*, ieag020. https://doi.org/10.1093/jisesa/ieag020.
109. Franceschi, V.; Nakata, P. Calcium oxalate in Plants: Formation and Function. *Annu. Rev. Plant Biol.* **2005**, *56*, 41–71. https://doi.org/10.1146/annurev.arplant.56.032604.144106.
110. Hirata, T.; Cabrero, P.; Berkholz, D.S.; Bondeson, D.P.; Ritman, E.L.; Thompson, J.R.; Dow, J.A.T.; Romero, M.F. In vivo Drosophilia genetic model for calcium oxalate nephrolithiasis. *Am. J. Physiol.-Ren. Physiol.* **2012**, *303*, F1555–F1562. https://doi.org/10.1152/ajprenal.00074.2012.
111. Wang, S.; Ju, Y.; Gao, L.; Miao, Y.; Qiao, H.; Wang, Y. The fruit fly kidney stone models and their application in drug development. *Heliyon* **2022**, *8*, e09232. https://doi.org/10.1016/j.heliyon.2022.e09232.
112. Ren, Y.; Li, Y.; Ju, Y.; Zhang, W.; Wang, Y. Insect cuticle and insecticide development. *Arch. Insect Biochem. Physiol.* **2023**, *114*, e22057. https://doi.org/10.1002/arch.22057.

113. Lu, Y.; Wu, Z.; Du, Z.; Lin, X.; Tian, E.; Zhang, F.; Chao, Z. The anti-urolithiasis activity and safety of strangury-relieving herbs: A comparative study based on fruit fly kidney stone model. *J. Ethnopharmacol.* **2024**, *326*, 117968. https://doi.org/10.1016/j.jep.2024.117968.
114. Yang, H.; Male, M.; Li, Y.; Wang, N.; Zhao, C.; Jin, S.; Hu, J.; Chen, Z.; Ye, Z.; Xu, H. Efficacy of Hydroxy-L-proline (HYP) analogs in the treatment of primary hyperoxaluria in Drosophila Melanogaster. *BMC Nephrol.* **2018**, *19*, 167. https://doi.org/10.1186/s12882-018-0980-8.
115. Han, S.; Zhao, C.; Pokhrel, G.; Sun, X.; Chen, Z.; Xu, H. Hydroxycitric Acid Tripotassium Inhibits Calcium Oxalate Crystal Formation in the Drosophila Melanogaster Model of Hyperoxaluria. *Med. Sci. Monit.* **2019**, *25*, 3662–3667. https://doi.org/10.12659/msm.913637.
116. Moe, O.W. Kidney stones: Pathophysiology and medical management. *Lancet* **2006**, *367*, 333–344. https://doi.org/10.1016/S0140-6736(06)68071-9.
117. Holmes, R.P.; Assimos, D.G. The impact of dietary oxalate on kidney stone formation. *Urol. Res.* **2004**, *32*, 311–316. https://doi.org/10.1007/s00240-004-0437-3.
118. Ju, Y.; Du, M.; Wang, Z.; Mu, X.; Miao, Y.; Guo, Z.; Wang, D.; Wang, S.; Ding, J.; Jin, G.; et al. Kukoamine A alleviates nephrolithiasis by inhibiting endogenous oxalate synthesis via the IL-6/JAK/STAT3/DAO signaling pathway. *Phytomedicine* **2024**, *135*, 156145. https://doi.org/10.1016/j.phymed.2024.156145.
119. Zhang, W.; Ren, Y.; Liu, X.; Liu, X.; Tang, W.; Ju, Y.; Yu, W.; Guo, Z.; Qiao, H.; Yu, X.; et al. The Medicine-Food Homology Fruit Gouqi (Lycium barbarum L.) Mitigates Kidney Stone via Multiple Bioactivities. *Nat. Product. Commun.* **2026**, *21,* 1-14 https://doi.org/10.1177/1934578X261451343.
120. Miller, A.W.; Dearing, D. The metabolic and ecological interactions of oxalate-degrading bacteria in the Mammalian gut. *Pathogens* **2013**, *2*, 636–652. https://doi.org/10.3390/pathogens2040636.
121. Li, S.; Wu, W.; Zhou, Y.; Zhang, S.; Wei, D.; Zhu, M.; Ying, X.; Sun, X.; Liu, H.; Zhu, W.; et al. Gut microbiota-regulated unconjugated bilirubin metabolism drives renal calcium oxalate crystal deposition. *Gut Microbes* **2025**, *17*, 2546158. https://doi.org/10.1080/19490976.2025.2546158.
122. Khan, S.R.; Kok, D.J. Modulators of urinary stone formation. *Front. Biosci.* **2004**, *9*, 1450–1482. https://doi.org/10.2741/1347.
123. Ali, S.N.; Dayarathna, T.K.; Ali, A.N.; Osumah, T.; Ahmed, M.; Cooper, T.T.; Power, N.E.; Zhang, D.; Kim, D.; Kim, R.; et al. Drosophila melanogaster as a function-based high-throughput screening model for antinephrolithiasis agents in kidney stone patients. *Dis. Model. Mech.* **2018**, *11*, dmm035873. https://doi.org/10.1242/dmm.035873.
124. Sun, X.Y.; Ouyang, J.M.; Liu, A.J.; Ding, Y.M.; Gan, Q.Z. Preparation, characterization, and in vitro cytotoxicity of COM and COD crystals with various sizes. *Mater. Sci. Eng. C Mater. Biol. Appl.* **2015**, *57*, 147–156. https://doi.org/10.1016/j.msec.2015.07.032.
125. Sun, P.; Liao, S.G.; Yang, R.Q.; Lu, C.L.; Ji, K.L.; Cao, D.H.; Hu, H.B.; Lu, J.M.; Song, X.Z.; Wu, M.; et al. Aspidopterys obcordata vine inulin fructan affects urolithiasis by modifying calcium oxalate crystallization. *Carbohydr. Polym.* **2022**, *294*, 119777. https://doi.org/10.1016/j.carbpol.2022.119777.
126. Palierse, E.; Przybylski, C.; Brouri, D.; Jolivalt, C.; Coradin, T. Interactions of Calcium with Chlorogenic and Rosmarinic Acids: An Experimental and Theoretical Approach. *Int. J. Mol. Sci.* **2020**, *21*, 4948.
127. Hong, S.-Y.; Qin, B.-L. The Protective Role of Dietary Polyphenols in Urolithiasis: Insights into Antioxidant Effects and Mechanisms of Action. *Nutrients* **2023**, *15*, 3753.
128. Ren, Y.; Zhang, W.; Li, Y.; Wang, D.; Tang, W.; Wang, L.; Jin, X.; Xia, D.; Yu, W.; Miao, Y.; et al. Streamlined strategy for discovering active compounds for nephrolithiasis treatment from herbal medicines using the fruit fly model. *J. Ethnopharmacol.* **2025**, *348*, 119846. https://doi.org/10.1016/j.jep.2025.119846.
129. Cao, L.C.; Honeyman, T.W.; Cooney, R.; Kennington, L.; Scheid, C.R.; Jonassen, J.A. Mitochondrial dysfunction is a primary event in renal cell oxalate toxicity. *Kidney Int.* **2004**, *66*, 1890–1900. https://doi.org/10.1111/j.1523-1755.2004.00963.x.

130. Albert, A.; Paul, E.; Rajakumar, S.; Saso, L. Oxidative stress and endoplasmic stress in calcium oxalate stone disease: The chicken or the egg? *Free Radic. Res.* **2020**, *54*, 244–253. https://doi.org/10.1080/10715762.2020.1751835.
131. Chen, S.J.; Dalanbaatar, S.; Chen, H.Y.; Wang, S.J.; Lin, W.Y.; Liu, P.L.; Tsai, M.Y.; Chen, D.C.; Chen, Y.H.; Chen, W.C. Astragalus membranaceus Extract Prevents Calcium Oxalate Crystallization and Extends Lifespan in a Drosophila Urolithiasis Model. *Life* **2022**, *12*, 1250. https://doi.org/10.3390/life12081250.
132. Huang, F.; Sun, X.Y.; Ouyang, J.M. Preparation and characterization of selenized Astragalus polysaccharide and its inhibitory effect on kidney stones. *Mater. Sci. Eng. C Mater. Biol. Appl.* **2020**, *110*, 110732. https://doi.org/10.1016/j.msec.2020.110732.
133. Sheng, B.W.; Chen, X.F.; Zhao, J.; He, D.L.; Nan, X.Y. Astragalus membranaceus reduces free radical-mediated injury to renal tubules in rabbits receiving high-energy shock waves. *Chin. Med. J.* **2005**, *118*, 43–49.
134. Liu, P.; Zhao, H.; Luo, Y. Anti-Aging Implications of Astragalus Membranaceus (Huangqi): A Well-Known Chinese Tonic. *Aging Dis.* **2017**, *8*, 868–886. https://doi.org/10.14336/ad.2017.0816.
135. Zhang, S.; Zhu, J.; Ju, Y.; Lv, M.; Yang, R.; Li, Y.; Miao, Y.; Wang, Y. Drosophila model and network pharmacology to explore novel targets and novel active components of chinese traditional medications for treating kidney stones. *Pharmacol. Res.-Mod. Chin. Med.* **2023**, *6*, 100220. https://doi.org/10.1016/j.prmcm.2023.100220.
136. Yi, Q.; Wang, J.; Song, Y.; Guo, Z.; Lei, S.; Yang, X.; Li, L.; Gao, C.; Zhou, Z. Ascl2 facilitates IL-10 production in Th17 cells to restrain their pathogenicity in inflammatory bowel disease. *Biochem. Biophys. Res. Commun.* **2019**, *510*, 435–441. https://doi.org/10.1016/j.bbrc.2019.01.123.
137. Xiu, M.; Li, B.; He, L.; Shi, Y.; Zhang, Y.; Zhou, S.; Liu, Y.; Wang, N.; He, J. Caffeic Acid Protects Against Ulcerative Colitis via Inhibiting Mitochondrial Apoptosis and Immune Overactivation in Drosophila. *Drug Des. Dev. Ther.* **2025**, *19*, 2157–2172. https://doi.org/10.2147/dddt.S499284.
138. Inohara, N.; Ogura, Y.; Fontalba, A.; Gutierrez, O.; Pons, F.; Crespo, J.; Fukase, K.; Inamura, S.; Kusumoto, S.; Hashimoto, M.; et al. Host Recognition of Bacterial Muramyl Dipeptide Mediated through NOD2: Implications for Crohn's Disease. *J. Biol. Chem.* **2003**, *278*, 5509–5512. https://doi.org/10.1074/jbc.C200673200.
139. Zeng, C.; Liu, F.; Huang, Y.; Liang, Q.; He, X.; Li, L.; Xie, Y. Drosophila: An Important Model for Exploring the Pathways of Inflammatory Bowel Disease (IBD) in the Intestinal Tract. *Int. J. Mol. Sci.* **2024**, *25*, 12742. https://doi.org/10.3390/ijms252312742.
140. Zhang, G.; Gu, Y.; Dai, X. Protective Effect of Bilberry Anthocyanin Extracts on Dextran Sulfate Sodium-Induced Intestinal Damage in Drosophila melanogaster. *Nutrients* **2022**, *14*, 2875. https://doi.org/10.3390/nu14142875.
141. Yang, S.; Li, X.; Xiu, M.; Dai, Y.; Wan, S.; Shi, Y.; Liu, Y.; He, J. Flos puerariae ameliorates the intestinal inflammation of Drosophila via modulating the Nrf2/Keap1, JAK-STAT and Wnt signaling. *Front. Pharmacol.* **2022**, *13*, 893758. https://doi.org/10.3389/fphar.2022.893758.
142. Madi, J.R.; Outa, A.A.; Ghannam, M.; Hussein, H.M.; Shehab, M.; Hasan, Z.A.K.H.; Fayad, A.A.; Shirinian, M.; Rahal, E.A. Drosophila melanogaster as a Model System to Assess the Effect of Epstein-Barr Virus DNA on Inflammatory Gut Diseases. *Front. Immunol.* **2021**, *12*, 586930. https://doi.org/10.3389/fimmu.2021.586930.
143. Han, S.; Xiu, M.; Li, S.; Shi, Y.; Wang, X.; Lin, X.; Cai, H.; Liu, Y.; He, J. Exposure to cytarabine causes side effects on adult development and physiology and induces intestinal damage via apoptosis in Drosophila. *Biomed. Pharmacother.* **2023**, *159*, 114265. https://doi.org/10.1016/j.biopha.2023.114265.
144. Nagy, P.; Szatmári, Z.; Sándor, G.O.; Lippai, M.; Hegedűs, K.; Juhász, G. Drosophila Atg16 promotes enteroendocrine cell differentiation via regulation of intestinal Slit/Robo signaling. *Development* **2017**, *144*, 3990–4001. https://doi.org/10.1242/dev.147033.

145. Kim, H.; Yi, X.; Xue, H.; Yue, G.; Zhu, J.; Eh, T.; Wang, S.; Jin, L.H. Extracts ofHylotelephiumerythrostictum (miq.) H. Ohba ameliorate intestinal injury by scavenging ROS and inhibiting multiple signaling pathways in Drosophila. *BMC Complement. Med. Ther.* **2024**, *24*, 397. https://doi.org/10.1186/s12906-024-04686-w.
146. Lee, S.H.; Hwang, D.; Lee, J.W.; Goo, T.W.; Yun, E.Y. The Identification of a Glucuronyltransferase-Related Gene, GlcAT-S, with Putative Mucus Protection and Anti-Inflammatory Effects from Gut-Damaged Drosophila by Dextran Sulfate Sodium (DSS). *Biology* **2025**, *14*, 513. https://doi.org/10.3390/biology14050513.
147. Yang, D.; Xiu, M.; Jiang, X.; Kang, Q.; Fu, J.; Zhou, S.; Liu, Y.; He, J. Caffeic Acid Alleviates Chronic Sleep Deprivation-Induced Intestinal Damage by Inhibiting the IMD Pathway in Drosophila. *J. Inflamm. Res.* **2025**, *18*, 3485–3498. https://doi.org/10.2147/jir.S500892.
148. Xu, Y.; Li, X.; Miao, Y.; Liu, X.; Zang, X.; Meng, Q.; Li, Y.; Yang, F.; Yuchi, Z.; Wang, Y. Xuanfei Baidu decoction alleviates intestinal inflammation and modulates microbiota distribution by suppressing IMD/NF-κB and JNK signaling pathways. *Front. Microbiol.* **2026**, *17*, 1853715. https://doi.org/10.3389/fmicb.2026.1853715.
149. He, J.; Li, X.; Yang, S.; Shi, Y.; Dai, Y.; Han, S.; Wang, Y.; Lin, X.; Wei, B.; Liu, Y.; et al. Protective effect of astragalus membranaceus and its bioactive compounds against the intestinal inflammation in Drosophila. *Front. Pharmacol.* **2022**, *13*, 1019594. https://doi.org/10.3389/fphar.2022.1019594.
150. Zhou, S.; Chen, D.; Wang, S.; Jiang, X.; Li, J.; He, J.; Xiu, M. Danggui Buxue Decoction Alleviates Acute Hypoxia-Induced Sleep Disorders: Insights from in vivo and in silico Analyses. *Drug Des. Dev. Ther.* **2025**, *19*, 6925–6942. https://doi.org/10.2147/dddt.S525366.
151. Su, H.; Tan, Y.; Zhou, Z.; Wang, C.; Chen, W.; Wang, J.; Sun, H. Effect and mechanism of total ginsenosides repairing SDS-induced Drosophila enteritis model based on MAPK pathway. *Exp. Ther. Med.* **2023**, *26*, 369. https://doi.org/10.3892/etm.2023.12068.
152. Yan, L.; Guo, X.; Zhou, J.; Zhu, Y.; Zhang, Z.; Chen, H. Quercetin Prevents Intestinal Stem Cell Aging via Scavenging ROS and Inhibiting Insulin Signaling in Drosophila. *Antioxidants* **2022**, *12*, 59. https://doi.org/10.3390/antiox12010059.
153. Sheng, X.; Zhu, Y.; Zhou, J.; Yan, L.; Du, G.; Liu, Z.; Chen, H. Antioxidant Effects of Caffeic Acid Lead to Protection of Drosophila Intestinal Stem Cell Aging. *Front. Cell Dev. Biol.* **2021**, *9*, 735483. https://doi.org/10.3389/fcell.2021.735483.
154. Yan, L.; Zhou, J.; Yuan, L.; Ye, J.; Zhao, X.; Ren, G.; Chen, H. Silibinin alleviates intestinal inflammation via inhibiting JNK signaling in Drosophila. *Front. Pharmacol.* **2023**, *14*, 1246960. https://doi.org/10.3389/fphar.2023.1246960.
155. Khaerani, M.; Chaeratunnisa, R.; Salsabila, A.; Asbah, A.; Asri, R.M.; Shiratsuchi, A.; Nainu, F. Curcumin-mediated alleviation of dextran-induced leaky gut in Drosophila melanogaster. *Narra J.* **2024**, *4*, e743. https://doi.org/10.52225/narra.v4i1.743.
156. Jia, K.; Wu, L.; Li, Z.; Wei, T.; Fan, T.; Xiao, G. Thymoquinone Ameliorates Gut Epithelial Injury by Suppressing the JNK Signaling Pathway Based on Its Anti-Oxidant Property. *Food Sci. Nutr.* **2025**, *13*, e70113. https://doi.org/10.1002/fsn3.70113.
157. Cho, K.H.; Kang, S.O. The Gut Microbiota of Drosophila melanogaster: A Model for Host-Microbe Interactions in Metabolism, Immunity, Behavior, and Disease. *Microorganisms* **2025**, *13*, 2515. https://doi.org/10.3390/microorganisms13112515.
158. Shin, S.C.; Kim, S.-H.; You, H.; Kim, B.; Kim, A.C.; Lee, K.-A.; Yoon, J.-H.; Ryu, J.-H.; Lee, W.-J. Drosophila microbiome modulates host developmental and metabolic homeostasis via insulin signaling. *Science.* **2011**, *334*, 670–674. https://doi.org/10.1126/science.1212782.
159. Wang, D.; Zhang, Y.; Xu, M.; Sun, X.; Cui, X.; Wang, X.; Liu, D. Dietary Bacillus licheniformis improves the effect of Astragalus membranaceus extract on blood glucose by regulating antioxidation activity and intestinal microbiota in InR[E19]/TM2 Drosophila melanogaster. *PLoS ONE* **2022**, *17*, e0271177. https://doi.org/10.1371/journal.pone.0271177.

160. Liang, H.; Tao, S.; Wang, Y.; Zhao, J.; Yan, C.; Wu, Y.; Liu, N.; Qin, Y. Astragalus polysaccharide: Implication for intestinal barrier, anti-inflammation, and animal production. *Front. Nutr.* **2024**, *11*, 1364739. https://doi.org/10.3389/fnut.2024.1364739.
161. Wang, B.; Yan, L.; Guo, S.; Wen, L.; Yu, M.; Feng, L.; Jia, X. Structural Elucidation, Modification, and Structure-Activity Relationship of Polysaccharides in Chinese Herbs: A Review. *Front. Nutr.* **2022**, *9*, 908175. https://doi.org/10.3389/fnut.2022.908175.
162. Wang, L.; Gao, M.; Kang, G.; Huang, H. The Potential Role of Phytonutrients Flavonoids Influencing Gut Microbiota in the Prophylaxis and Treatment of Inflammatory Bowel Disease. *Front. Nutr.* **2021**, *8*, 798038. https://doi.org/10.3389/fnut.2021.798038.
163. Balaji, S.; Jeyaraman, N.; Jeyaraman, M.; Ramasubramanian, S.; Muthu, S.; Santos, G.S.; da Fonseca, L.F.; Lana, J.F. Impact of curcumin on gut microbiome. *World J. Exp. Med.* **2025**, *15*, 100275. https://doi.org/10.5493/wjem.v15.i1.100275.
164. Hanahan, D.; Weinberg, R.A. Hallmarks of cancer: The next generation. *Cell.* **2011**, *144*, 646–674. https://doi.org/10.1016/j.cell.2011.02.013.
165. Gong, S.; Zhang, Y.; Tian, A.; Deng, W.-M. Tumor models in various Drosophila tissues. *WIREs Mech. Dis.* **2021**, *13*, e1525. https://doi.org/10.1002/wsbm.1525.
166. Weina, T.; Ying, L.; Yiwen, W.; Huan-Huan, Q. What we have learnt from Drosophila model organism: The coordination between insulin signaling pathway and tumor cells. *Heliyon* **2022**, *8*, e09957. https://doi.org/10.1016/j.heliyon.2022.e09957.
167. Kamdem, J.P.; Duarte, A.E.; Ibrahim, M.; Lukong, K.E.; Barros, L.M.; Roeder, T. Bibliometric analysis of personalized humanized mouse and Drosophila models for effective combinational therapy in cancer patients. *Biochim. Biophys. Acta Mol. Basis Dis.* **2020**, *1866*, 165880. https://doi.org/10.1016/j.bbadis.2020.165880.
168. Bossen, J.; Uliczka, K.; Steen, L.; Pfefferkorn, R.; Mai, M.M.-Q.; Burkhardt, L.; Spohn, M.; Bruchhaus, I.; Fink, C.; Heine, H.; et al. An EGFR-Induced Drosophila Lung Tumor Model Identifies Alternative Combination Treatments. *Mol. Cancer Ther.* **2019**, *18*, 1659–1668. https://doi.org/10.1158/1535-7163.MCT-19-0168.
169. Brand, A.H.; Perrimon, N. Targeted gene expression as a means of altering cell fates and generating dominant phenotypes. *Development* **1993**, *118*, 401–415.
170. Brumby, A.M.; Richardson, H.E. Using Drosophila melanogaster to map human cancer pathways. *Nat. Rev. Cancer* **2005**, *5*, 626–639.
171. van Neerven, S.M.; Vermeulen, L. Cell competition in development, homeostasis and cancer. *Nat. Rev. Mol. Cell Biol.* **2022**, *24*, 221–236. https://doi.org/10.1038/s41580-022-00538-y.
172. Lee, T.; Luo, L. Mosaic analysis with a repressible cell marker (MARCM) for Drosophila neural development. *Trends Neurosci.* **2001**, *24*, 251–254. https://doi.org/10.1016/S0166-2236(00)01791-4.
173. Lee, T.; Luo, L. Mosaic analysis with a repressible cell marker for studies of gene function in neuronal morphogenesis. *Neuron* **1999**, *22*, 451–461.
174. Brumby, A.M.; Richardson, H.E. scribble mutants cooperate with oncogenic Ras or Notch to cause neoplastic overgrowth in Drosophila. *EMBO J.* **2003**, *22*, 5769–5779.
175. Pagliarini, R.A.; Xu, T. A genetic screen in Drosophila for metastatic behavior. *Science* **2003**, *302*, 1227–1231.
176. Abdullateef, R.; Ibekwe, J.P.; Oyoyo, H.; Ogbodo, S. Applications and challenges of Drosophila melanogaster as a laboratory model in human cancer research: A narrative review. *Discov. Oncol.* **2025**, *17*, 132. https://doi.org/10.1007/s12672-025-04248-7.
177. Zirin, J.; Bosch, J.; Viswanatha, R.; Mohr, S.E.; Perrimon, N. State-of-the-art CRISPR for in vivo and cell-based studies in Drosophila. *Trends Genet.* **2021**, *38*, 437–453. https://doi.org/10.1016/j.tig.2021.11.006.
178. Viswanatha, R.; Entwisle, S.; Hu, Y.; Kim, A.-R.; Reap, K.; Butnaru, M.; Qadiri, M.; Mohr, S.E.; Perrimon, N. Higher resolution pooled genome-wide CRISPR knockout screening in Drosophila

cells using integration and anti-CRISPR (IntAC). *Nat. Commun.* **2025**, *16*, 6498. https://doi.org/10.1038/s41467-025-61692-3.
179. Gaivão, I.; Sierra, L.M.; Comendador, M.A. The w/w+ SMART assay of Drosophila melanogaster detects the genotoxic effects of reactive oxygen species inducing compounds. *Mutat. Res./Genet. Toxicol. Environ. Mutagen.* **1999**, *440*, 139–145. https://doi.org/10.1016/S1383-5718(99)00020-0.
180. Heres-Pulido, M. Genotoxicity of tamoxifen citrate and 4-nitroquinoline-1-oxide in the wing spot test of Drosophila melanogaster. *Mutagenesis* **2004**, *19*, 187–193. https://doi.org/10.1093/mutage/geh020.
181. Gaivão, I.; Sierra, M.; Ferreira, J. The *w*/*w* + Somatic Mutation and Recombination Test (SMART) of *Drosophila melanogaster* for Detecting Antigenotoxic Activity. In *Genotoxicity and Mutagenicity—Mechanisms and Test Methods*; Soloneski, S., Larramendy, M.L., Eds.; IntechOpen: London, UK, 2020. https://doi.org/10.5772/intechopen.91630.
182. Sakane, F.; Hoshino, F.; Ebina, M.; Sakai, H.; Takahashi, D. The Roles of Diacylglycerol Kinase α in Cancer Cell Proliferation and Apoptosis. *Cancers*. **2021**, *13*, 5190. https://doi.org/10.3390/cancers13205190.
183. Sun, K.; Jin, L.; Karolová, J.; Vorwerk, J.; Hailfinger, S.; Opalka, B.; Zapukhlyak, M.; Lenz, G.; Khandanpour, C. Combination Treatment Targeting mTOR and MAPK Pathways Has Synergistic Activity in Multiple Myeloma. *Cancers*. **2023**, *15*, 2373. https://doi.org/10.3390/cancers15082373.
184. Shen, Y.; Tang, Y.; Fan, X.; Qin, X.; Wang, W.; Li, Q. Vitamin B3 suppresses RasV12 -driven tumor growth in Drosophila via redox regulation, mitochondrial function, and autophagy activation. *Transl. Cancer Res.* **2026**, *15*, 207. https://doi.org/10.21037/tcr-2025-aw-2397.
185. Ma, Y.-S.; Chueh, F.-S.; Kuo, Y.-H.; Hsieh, Y.-S.; Yu, S.-N.; Chen, J.-Y.; Lin, W.-Y.; Chen, J.-C.; Chen, C.-Y.; Hsieh, W.-T.; et al. Physalin A Suppresses Human Oral Squamous Carcinoma Cell Migration and Invasion Through Inhibiting Grb2/Ras and MMP/uPA Signaling Pathways. *In Vivo* **2026**, *40*, 826–845. https://doi.org/10.21873/invivo.14240.
186. Raghuvanshi, V.; Singh, J.; Sharma, S.; Butcher, R.; Srikrishna, S.; Katiyar, D. Anti-metastatic cancer potential of 4H-chromene derivatives targeting JNK in Scribble knockdown induced epithelial cancer model: In vivo and in silico studies. *Bioorg Chem.* **2025**, *163*, 108741. https://doi.org/10.1016/j.bioorg.2025.108741.
187. Harvey, K.F.; Pfleger, C.M.; Hariharan, I.K. The Drosophila Mst ortholog, hippo, restricts growth and cell proliferation and promotes apoptosis. *Cell* **2003**, *114*, 457–467.
188. Wu, S.; Huang, J.; Dong, J.; Pan, D. hippo Encodes a Ste-20 Family Protein Kinase that Restricts Cell Proliferation and Promotes Apoptosis in Conjunction with salvador and warts. *Cell* **2003**, *114*, 445–456. https://doi.org/10.1016/S0092-8674(03)00549-X.
189. Dey, A.; Varelas, X.; Guan, K.-L. Targeting the Hippo pathway in cancer, fibrosis, wound healing and regenerative medicine. *Nat. Rev. Drug Discov.* **2020**, *19*, 480–494. https://doi.org/10.1038/s41573-020-0070-z.
190. Aguiar, W.S.; Pires, I.V.A.; Barros, L.M.L.; Almeida, P.M.; Sá, J.L.S.; Lima-Neto, B.S.; Martins, F.A. The Anticarcinogenicity Effects of Piperine and 4,5-Dihydroxypiperine in Drosophila melanogaster Somatic Cells. *ACS Omega* **2026**, *11*, 22072–22081. https://doi.org/10.1021/acsomega.5c13279.
191. Jiang, B.; Chen, S.; Qian, W.; Yan, X.; Li, Y. Screening of effective components of Psoralea Fructus for endometrial cancer by affinity ultrafiltration chromatography-mass spectrometry. *Chin. Tradit. Herbal. Drugs* **2024**, *55*, 4663–4669.
192. Dar, A.C.; Das, T.K.; Shokat, K.M.; Cagan, R.L. Chemical genetic discovery of targets and anti-targets for cancer polypharmacology. *Nature* **2012**, *486*, 80–84. https://doi.org/10.1038/nature11127.
193. Jiang, H.; Kimura, T.; Hai, H.; Yamamura, R.; Sonoshita, M. Drosophila as a toolkit to tackle cancer and its metabolism. *Front. Oncol.* **2022**, *12*, 982751. https://doi.org/10.3389/fonc.2022.982751.
194. Lee, Y.; Lee, S.-H.; Choi, D.; Nam, H.-S.; Kim, K.D.; Choi, M.H.; Cho, M.-K.; Lee, Y.-J. Resveratrol Targets Glycolytic Enzymes HK II and PKM2 to Promote Concurrent Apoptotic and Necrotic Cell

Death in Malignant Melanoma. *Curr. Issues Mol. Biol.* **2025**, *47*, 1006. https://doi.org/10.3390/cimb47121006.
195. Gao, Y.; Wang, Y.Y.; Wang, B.D.; Hu, Q.Y.; Jiang, J.R.; Feng, B.; Gao, X.L.; Liu, L.K.; Zhu, W.B.; Yue, L.L. Mechanism of Action of Resveratrol Affecting the Biological Function of Breast Cancer Through the Glycolytic Pathway. *World J. Oncol.* **2025**, *16*, 375–387. https://doi.org/10.14740/wjon2586.
196. Wei, R.; Hackman, R.M.; Wang, Y.; Mackenzie, G.G. Targeting Glycolysis with Epigallocatechin-3-Gallate Enhances the Efficacy of Chemotherapeutics in Pancreatic Cancer Cells and Xenografts. *Cancers*. **2019**, *11*, 1496. https://doi.org/10.3390/cancers11101496.
197. Wei, R.; Mao, L.; Xu, P.; Zheng, X.; Hackman, R.M.; Mackenzie, G.G.; Wang, Y. Suppressing glucose metabolism with epigallocatechin-3-gallate (EGCG) reduces breast cancer cell growth in preclinical models. *Food Funct.* **2018**, *9*, 5682–5696. https://doi.org/10.1039/c8fo01397g.
198. Jin, L.; Gao, F.; Li, P.; Yu, C.; Xiao, G. Genetic dissection of signalling pathways that mediate iron-related tumor growth in a Drosophila model. *PLoS Genet.* **2026**, *22*, e1012017. https://doi.org/10.1371/journal.pgen.1012017.
199. Bakopoulos, D.; Golenkina, S.; Dark, C.; Christie, E.L.; Sánchez-Sánchez, B.J.; Stramer, B.M.; Cheng, L.Y. Convergent insulin and TGF-β signalling drives cancer cachexia by promoting aberrant fat body ECM accumulation in a Drosophila tumour model. *EMBO Rep.* **2023**, *24*, e57695. https://doi.org/10.15252/embr.202357695.
200. Khezri, R.; Holland, P.; Schoborg, T.A.; Abramovich, I.; Takáts, S.; Dillard, C.; Jain, A.; O'Farrell, F.; Schultz, S.W.; Hagopian, W.M.; et al. Host autophagy mediates organ wasting and nutrient mobilization for tumor growth. *EMBO J.* **2021**, *40*, e107336. https://doi.org/10.15252/embj.2020107336.
201. Singaravelan, N.; Tollefsbol, T.O. Polyphenol-Based Prevention and Treatment of Cancer Through Epigenetic and Combinatorial Mechanisms. *Nutrients* **2025**, *17*, 616.
202. Altomare, C.; Macrì, R.; Serra, M.; Ussia, S.; Ritorto, G.; Maiuolo, J.; Muscoli, C.; Perri, E.; Mollace, V. The Potential of Nutraceutical Supplementation in Counteracting Cancer Development and Progression: A Pathophysiological Perspective. *Nutrients* **2025**, *17*, 2354.
203. Elovsson, G.; Bergkvist, L.; Brorsson, A.-C. Exploring Aβ Proteotoxicity and Therapeutic Candidates Using Drosophila melanogaster. *Int. J. Mol. Sci.* **2021**, *22*, 10448. https://doi.org/10.3390/ijms221910448.
204. Elovsson, G.; Klingstedt, T.; Brown, M.; Nilsson, K.P.R.; Brorsson, A.-C. A Novel Drosophila Model of Alzheimer's Disease to Study Aβ Proteotoxicity in the Digestive Tract. *Int. J. Mol. Sci.* **2024**, *25*, 2105. https://doi.org/10.3390/ijms25042105.
205. Tsintzas, E.; Niccoli, T. Using Drosophila amyloid toxicity models to study Alzheimer's disease. *Ann. Human. Genet.* **2024**, *88*, 349–363. https://doi.org/10.1111/ahg.12554.
206. Aggidis, A.; Devitt, G.; Zhang, Y.; Chatterjee, S.; Townsend, D.; Fullwood, N.J.; Ortega, E.R.; Tarutani, A.; Hasegawa, M.; Cooper, A.; et al. A novel peptide-based tau aggregation inhibitor as a potential therapeutic for Alzheimer's disease and other tauopathies. *Alzheimers Dement.* **2024**, *20*, 7788–7804. https://doi.org/10.1002/alz.14246.
207. Gandini, A.; Gonçalves, A.E.; Strocchi, S.; Albertini, C.; Janočková, J.; Tramarin, A.; Grifoni, D.; Poeta, E.; Soukup, O.; Muñoz-Torrero, D.; et al. Discovery of Dual Aβ/Tau Inhibitors and Evaluation of Their Therapeutic Effect on a Drosophila Model of Alzheimer's Disease. *ACS Chem. Neurosci.* **2022**, *13*, 3314–3329. https://doi.org/10.1021/acschemneuro.2c00357.
208. Cesur, M.F.; Strazdins, R.; Sarkar, S.; Çakır, T. In vivo and in silico models of Drosophila for Parkinson's disease. *FEBS J.* **2025**, *292*, 4753–4773. https://doi.org/10.1111/febs.70140.
209. Skoulakis, E.M.C.; Carvajal-Oliveros, A.; Dominguez-Baleón, C.; Sánchez-Díaz, I.; Zambrano-Tipan, D.; Hernández-Vargas, R.; Campusano, J.M.; Narváez-Padilla, V.; Reynaud, E. Parkinsonian phenotypes induced by Synphilin-1 expression are differentially contributed by serotonergic and

dopaminergic circuits and suppressed by nicotine treatment. *PLoS ONE* **2023**, *18*, , e0282348. https://doi.org/10.1371/journal.pone.0282348.
210. Schaffner, A.; Li, X.; Gomez-Llorente, Y.; Leandrou, E.; Memou, A.; Clemente, N.; Yao, C.; Afsari, F.; Zhi, L.; Pan, N.; et al. Vitamin B12 modulates Parkinson's disease LRRK2 kinase activity through allosteric regulation and confers neuroprotection. *Cell Res.* **2019**, *29*, 313–329. https://doi.org/10.1038/s41422-019-0153-8.
211. Imai, Y. PINK1-Parkin signaling in Parkinson's disease: Lessons from Drosophila. *Neurosci. Res.* **2020**, *159*, 40–46. https://doi.org/10.1016/j.neures.2020.01.016.
212. Von Stockum, S.; Nardin, A.; Schrepfer, E.; Ziviani, E. Mitochondrial dynamics and mitophagy in Parkinson's disease: A fly point of view. *Neurobiol. Dis.* **2015**, *90*, 58–67. https://doi.org/10.1016/j.nbd.2015.11.002.
213. Yang, J.; Kim, M.J.; Yoon, W.; Kim, E.Y.; Kim, H.; Lee, Y.; Min, B.; Kang, K.S.; Son, J.H.; Park, H.T.; et al. Isocitrate protects DJ-1 null dopaminergic cells from oxidative stress through NADP+-dependent isocitrate dehydrogenase (IDH). *PLoS Genet.* **2017**, *13*, e1006975. https://doi.org/10.1371/journal.pgen.1006975.
214. Joshi, D.; Kulkarni, M.; Parekh, P.; Shah, S.; Greig, N.H.; Acharya, S. Targeting protein kinases in Parkinson's disease: The emerging role of phytoconstituents. *Nutr. Neurosci.* **2025**, *28*, 1532–1563. https://doi.org/10.1080/1028415X.2025.2531356.
215. Nallusamy, S.; Selvamani, S.B.; Sathyaseelan, C.; Selvakumar, D.; Panigrahi, R. Targeting PINK1 using phytochemicals: Exploring molecular insights into Parkinson's disease therapeutics. *Biochem. Cell Biol.* **2025**, *103*, 1–55. https://doi.org/10.1139/bcb-2024-0280.
216. Fathy, M.; Seif, A.; El Kholy, S. Antioxidants and Parkinson's Disease: A Systematic Review and Meta-Analysis of Research Evidence and Prospectives Using the Drosophila Model. *J. Exp. Zool. A Ecol. Integr. Physiol.* **2025**, *345*, 85–104. https://doi.org/10.1002/jez.70042.
217. Li, W.; Pan, X.; Li, M.; Ling, L.; Zhang, M.; Liu, Z.; Zhang, K.; Guo, J.; Wang, H. Impact of age on the rotenone-induced sporadic Parkinson's disease model using Drosophila melanogaster. *Neurosci. Lett.* **2023**, *805*, 137187. https://doi.org/10.1016/j.neulet.2023.137187.
218. Okonta, C.; Ogunyemi, O.M.; Olabuntu, B.; Abolaji, A.O. Ameliorative role of naringenin in MPTP-induced Parkinsonism: Insights from Drosophila melanogaster experimental model combined with computational biology. *Toxicol. Rep.* **2025**, *14*, 102004. https://doi.org/10.1016/j.toxrep.2025.102004.
219. Poleto, K.H.; Janner, D.E.; Dahleh, M.M.M.; Poetini, M.R.; Fernandes, E.J.; Musachio, E.A.S.; de Almeida, F.P.; Amador, E.C.d.M.; Reginaldo, J.C.; Carriço, M.R.S.; et al. p-Coumaric acid potential in restoring neuromotor function and oxidative balance through the Parkin pathway in a Parkinson disease-like model in Drosophila melanogaster. *Food Chem. Toxicol.* **2024**, *193*, 115002. https://doi.org/10.1016/j.fct.2024.115002.
220. Tan, F.H.P.; Hadri, N.A.B.; Najimudin, N.; Watanabe, N.; Azzam, G. Ethyl caffeate ameliorated amyloid-beta42 protein-associated toxicity in PC12 cells and Drosophila melanogaster. *Geriatr. Gerontol. Int.* **2021**, *21*, 1125–1130. https://doi.org/10.1111/ggi.14296.
221. Varshney, H.; Gaur, K.; Subhan, I.; Fatima, J.; Jyoti, S.; Mantasha, I.; Shahid, M.; Rahul; Siddique, Y.H. Neuroprotective Effects of Fenugreek Leaf Extract in a Drosophila Model of Alzheimer's Disease Expressing Human Aβ-42. *Curr. Alzheimer Res.* **2025**, *22*, 587–612. https://doi.org/10.2174/0115672050385870250721072643.
222. Kong, Y.; Li, K.; Fu, T.; Wan, C.; Zhang, D.; Song, H.; Zhang, Y.; Liu, N.; Gan, Z.; Yuan, L. Quercetin ameliorates Aβ toxicity in Drosophila AD model by modulating cell cycle-related protein expression. *Oncotarget* **2016**, *7*, 67716–67731. https://doi.org/10.18632/oncotarget.11963.
223. Sun, W.; Lee, S.; Huang, X.; Liu, S.; Inayathullah, M.; Kim, K.-M.; Tang, H.; Ashford, J.W.; Rajadas, J. Attenuation of synaptic toxicity and MARK4/PAR1-mediated Tau phosphorylation by methylene blue for Alzheimer's disease treatment. *Sci. Rep.* **2016**, *6*, 34784. https://doi.org/10.1038/srep34784.

224. Anupama, K.P.; Shilpa, O.; Antony, A.; Raghu, S.V.; Gurushankara, H.P. Jatamansinol from Nardostachys jatamansi (D.Don) DC. Protects Aβ42-Induced Neurotoxicity in Alzheimer's Disease Drosophila Model. *Neurotoxicology* **2022**, *90*, 62–78. https://doi.org/10.1016/j.neuro.2022.02.011.
225. Zhang, Y.; Deng, L.; Wei, J.; Huang, L.; Gao, F.; Yu, L.; Zhu, F.; Mi, J.; Wu, J.; Ren, F.; et al. Gaultheria leucocarpa inhibits Aβ fibrillization and enhances mitophagy-mediated degradation of pathogenic proteins. *Neurotherapeutics* **2025**, *22*, e00721. https://doi.org/10.1016/j.neurot.2025.e00721.
226. Tan, F.H.P.; Azzam, G.; Najimudin, N.; Shamsuddin, S.; Zainuddin, A.; Kasihmuddin, M.S.M. Salvianolic acid B ameliorates Aβ42 toxicity in Aβ42-expressing Drosophila model: Behavioral and transcriptomic profiling. *Metab. Brain Dis.* **2025**, *40*, 204. https://doi.org/10.1007/s11011-025-01625-7.
227. Ucar Akyurek, T.; Senol Deniz, F.S.; Erdogan Orhan, I.; Gozuboyuk, M.; Erdemli, G.I.; Emecen, G. Gallic Acid, 3-Hydroxytyrosol, and Quercetin Modulate Cholinesterase Activity in Drosophila melanogaster. *Int. J. Mol. Sci.* **2026**, *27*, 859.
228. Lin, X.; Wen, X.; Wei, Z.; Guo, K.; Shi, F.; Huang, T.; Wang, W.; Zheng, J. Vitamin K2 protects against Aβ42-induced neurotoxicity by activating autophagy and improving mitochondrial function in Drosophila. *NeuroReport* **2021**, *32*, 431–437. https://doi.org/10.1097/wnr.0000000000001599.
229. Bai, X.-L.; Luo, Y.-J.; Fan, W.-Q.; Zhang, Y.-M.; Liao, X. Neuroprotective Effects of Lycium Barbarum Fruit Extract on Pink1B9Drosophila Melanogaster Genetic Model of Parkinson's Disease. *Plant Foods Human. Nutr.* **2022**, *78*, 68–75. https://doi.org/10.1007/s11130-022-01016-8.
230. Liu, M.; Yu, S.; Wang, J.; Qiao, J.; Liu, Y.; Wang, S.; Zhao, Y. Ginseng protein protects against mitochondrial dysfunction and neurodegeneration by inducing mitochondrial unfolded protein response in Drosophila melanogaster PINK1 model of Parkinson's disease. *J. Ethnopharmacol.* **2019**, *247*, 112213. https://doi.org/10.1016/j.jep.2019.112213.
231. Gonçalves, D.F.; Senger, L.R.; Foletto, J.V.P.; Michelotti, P.; Soares, F.A.A.; Dalla Corte, C.L. Caffeine improves mitochondrial function in PINK1B9-null mutant Drosophila melanogaster. *J. Bioenerg. Biomembr.* **2022**, *55*, 1–13. https://doi.org/10.1007/s10863-022-09952-5.
232. Wu, Z.; Wu, A.; Dong, J.; Sigears, A.; Lu, B. Grape skin extract improves muscle function and extends lifespan of a Drosophila model of Parkinson's disease through activation of mitophagy. *Exp. Gerontol.* **2018**, *113*, 10–17. https://doi.org/10.1016/j.exger.2018.09.014.
233. Chaouhan, H.S.; Li, X.; Sun, K.-T.; Wang, I.K.; Yu, T.-M.; Yu, S.-H.; Chen, K.-B.; Lin, W.-Y.; Li, C.-Y. Calycosin Alleviates Paraquat-Induced Neurodegeneration by Improving Mitochondrial Functions and Regulating Autophagy in a Drosophila Model of Parkinson's Disease. *Antioxidants* **2022**, *11*, 222. https://doi.org/10.3390/antiox11020222.
234. Hartman, R.; Lopez Rodriguez, Z.; Morgan, M. Polyphenol diet and exercise as neuroprotective factors in a Drosophila model of Alzheimer's disease. *Anim. Models Exp. Med.* **2026**, *9*, 217–236. https://doi.org/10.1002/ame2.70106.
235. Adedara, A.O.; Babalola, A.D.; Stephano, F.; Awogbindin, I.O.; Olopade, J.O.; Rocha, J.B.T.; Whitworth, A.J.; Abolaji, A.O. An assessment of the rescue action of resveratrol in parkin loss of function-induced oxidative stress in Drosophila melanogaster. *Sci. Rep.* **2022**, *12*, 3922. https://doi.org/10.1038/s41598-022-07909-7.
236. Inoue, E.; Suzuki, T.; Shimizu, Y.; Sudo, K.; Kawasaki, H.; Ishida, N. Saffron ameliorated motor symptoms, short life span and retinal degeneration in Parkinson's disease fly models. *Gene* **2021**, *799*, 145811. https://doi.org/10.1016/j.gene.2021.145811.
237. Ishola, I.O.; Afolayan, O.; Odutola, I.; Faniyan, O.; Adeyemi, O. Therapeutic potential of hesperidin in Parkinson's disease with dementia: Inhibition of alpha synuclein and amyloid beta in Drosophila melanogaster. *Niger. J. Physiol. Sci.* **2021**, *36*, 43–48.
238. Ayikobua, E.T.; Kasolo, J.; Kasozi, K.I.; Eze, E.D.; Safiriyu, A.; Ninsiima, H.I.; Kiyimba, K.; Namulema, J.; Jjesero, E.; Ssempijja, F.; et al. Synergistic action of propolis with levodopa in the management of Parkinsonism in Drosophila melanogaster. *J. Complement. Integr. Med.* **2020**, *17*, 20190136. https://doi.org/10.1515/jcim-2019-0136.

239. Adedara, A.O.; Otenaike, T.A.; Farodoye, O.M.; Abolaji, A.O. Ellagic acid mitigates rotenone-induced damage via modulating mitochondria function in Drosophila melanogaster. *J. Biochem. Mol. Toxicol.* **2023**, *37*, e23332. https://doi.org/10.1002/jbt.23332.
240. Ortega-Regules, A.E.; Martínez-Thomas, J.A.; Schürenkämper-Carrillo, K.; de Parrodi, C.A.; López-Mena, E.R.; Mejía-Méndez, J.L.; Lozada-Ramírez, J.D. Recent Advances in the Therapeutic Potential of Carotenoids in Preventing and Managing Metabolic Disorders. *Plants*. **2024**, *13*, 1584. https://doi.org/10.3390/plants13121584.
241. Chaves, N.S.G.; Janner, D.E.; Poetini, M.R.; Fernandes, E.J.; de Almeida, F.P.; Musachio, E.A.S.; Reginaldo, J.C.; Dahleh, M.M.M.; de Carvalho, A.S.; Leimann, F.V.; et al. β-carotene-loaded nanoparticles protect against neuromotor damage, oxidative stress, and dopamine deficits in a model of Parkinson's disease in Drosophila melanogaster. *Comp. Biochem. Physiol. Part C Toxicol. Pharmacol.* **2023**, *268*, 109615. https://doi.org/10.1016/j.cbpc.2023.109615.
242. Lee, D.H.; Kim, C.S.; Lee, Y.J. Astaxanthin protects against MPTP/MPP+-induced mitochondrial dysfunction and ROS production in vivo and in vitro. *Food Chem. Toxicol.* **2011**, *49*, 271–280. https://doi.org/10.1016/j.fct.2010.10.029.
243. Luo, S.; Zhang, L.; Wu, X.; Wang, J.; Li, Q.; Chen, W.; Li, R.; Zhou, L.; Zhou, N.; Chen, R.; et al. Therapeutic potential of neuroprotective plant extracts in Parkinson's disease. *Front. Neurosci.* **2026**, *20*, 1813133. https://doi.org/10.3389/fnins.2026.1813133.
244. Cord, D.; Rîmbu, M.C.; Iordache, M.P.; Albulescu, R.; Pop, S.; Tanase, C.; Popa, M.-L. Phytochemicals as Epigenetic Modulators in Chronic Diseases: Molecular Mechanisms. *Molecules* **2025**, *30*, 4317.
245. Agrawal, P.; Chung, P.; Heberlein, U.; Kent, C. Enabling cell-type-specific behavioral epigenetics in Drosophila: A modified high-yield INTACT method reveals the impact of social environment on the epigenetic landscape in dopaminergic neurons. *BMC Biol.* **2019**, *17*, 30. https://doi.org/10.1186/s12915-019-0646-4.
246. Chattopadhyay, D.; Chitnis, A.; Talekar, A.; Mulay, P.; Makkar, M.; James, J.; Thirumurugan, K. Hormetic efficacy of rutin to promote longevity in Drosophila melanogaster. *Biogerontology* **2017**, *18*, 397–411. https://doi.org/10.1007/s10522-017-9700-1.
247. De Lazzari, F.; Sandrelli, F.; Whitworth, A.J.; Bisaglia, M. Antioxidant Therapy in Parkinson's Disease: Insights from Drosophila melanogaster. *Antioxidants* **2020**, *9*, 52.
248. Cho, K.; Myeongcheol, S.; Kim, S.; Lee, S. Recent Advances in Studies on the Therapeutic Potential of Dietary Carotenoids in Neurodegenerative Diseases. *Oxidative Med. Cell. Longev.* **2018**, *2018*, 4120458. https://doi.org/10.1155/2018/4120458.
249. Papanikolopoulou, K.; Mudher, A.; Skoulakis, E. An assessment of the translational relevance of Drosophila in drug discovery. *Expert. Opin. Drug Discov.* **2019**, *14*, 303–313. https://doi.org/10.1080/17460441.2019.1569624.
250. Feyereisen, R. 8—Insect CYP Genes and P450 Enzymes. In Insect Molecular Biology and Biochemistry; Gilbert, L.I., Ed.; Academic Press: San Diego, CA, USA, 2012; pp. 236–316. https://doi.org/10.1016/B978-0-12-384747-8.10008-X.
251. Tijet, N.; Helvig, C.; Feyereisen, R. The cytochrome P450 gene superfamily in Drosophila melanogaster: Annotation, intron-exon organization and phylogeny. *Gene* **2001**, *262*, 189–198. https://doi.org/10.1016/S0378-1119(00)00533-3.
252. Guengerich, F.P. Cytochrome P450 and Chemical Toxicology. *Chem. Res. Toxicol.* **2007**, *21*, 70–83. https://doi.org/10.1021/tx700079z.
253. Zanger, U.M.; Schwab, M. Cytochrome P450 enzymes in drug metabolism: Regulation of gene expression, enzyme activities, and impact of genetic variation. *Pharmacol. Ther.* **2013**, *138*, 103–141. https://doi.org/10.1016/j.pharmthera.2012.12.007.

254. Jowett, T.; Wajidi, M.F.; Oxtoby, E.; Wolf, C.R. Mammalian genes expressed in Drosophila: A transgenic model for the study of mechanisms of chemical mutagenesis and metabolism. *EMBO J.* **1991**, *10*, 1075–1081. https://doi.org/10.1002/j.1460-2075.1991.tb08047.x.
255. Cheesman, M.J.; Traylor, M.J.; Hilton, M.E.; Richards, K.E.; Taylor, M.C.; Daborn, P.J.; Russell, R.J.; Gillam, E.M.J.; Oakeshott, J.G. Soluble and membrane-bound Drosophila melanogaster CYP6G1 expressed in Escherichia coli: Purification, activity, and binding properties toward multiple pesticides. *Insect Biochem. Mol. Biol.* **2013**, *43*, 455–465. https://doi.org/10.1016/j.ibmb.2013.02.003.
256. King-Jones, K.; Horner, M.A.; Lam, G.; Thummel, C.S. The DHR96 nuclear receptor regulates xenobiotic responses in Drosophila. *Cell Metab.* **2006**, *4*, 37–48. https://doi.org/10.1016/j.cmet.2006.06.006.
257. Chung, H.; Bogwitz, M.R.; McCart, C.; Andrianopoulos, A.; Ffrench-Constant, R.H.; Batterham, P.; Daborn, P.J. Cis-regulatory elements in the Accord retrotransposon result in tissue-specific expression of the Drosophila melanogaster insecticide resistance gene Cyp6g1. *Genetics* **2007**, *175*, 1071–1077. https://doi.org/10.1534/genetics.106.066597.
258. Hindle, S.J.; Bainton, R.J. Barrier mechanisms in the Drosophila blood-brain barrier. *Front. Neurosci.* **2014**, *8*, 414. https://doi.org/10.3389/fnins.2014.00414.
259. Stork, T.; Engelen, D.; Krudewig, A.; Silies, M.; Bainton, R.J.; Klämbt, C. Organization and function of the blood-brain barrier in Drosophila. *J. Neurosci.* **2008**, *28*, 587–597. https://doi.org/10.1523/jneurosci.4367-07.2008.
260. Limmer, S.; Weiler, A.; Volkenhoff, A.; Babatz, F.; Klämbt, C. The Drosophila blood-brain barrier: Development and function of a glial endothelium. *Front. Neurosci.* **2014**, *8*, 365. https://doi.org/10.3389/fnins.2014.00365.
261. DeSalvo, M.K.; Mayer, N.; Mayer, F.; Bainton, R.J. Physiologic and anatomic characterization of the brain surface glia barrier of Drosophila. *Glia* **2011**, *59*, 1322–1340. https://doi.org/10.1002/glia.21147.

Disclaimer/Publisher's Note: The statements, opinions and data contained in all publications are solely those of the individual author(s) and contributor(s) and not of MDPI and/or the editor(s). MDPI and/or the editor(s) disclaim responsibility for any injury to people or property resulting from any ideas, methods, instructions or products referred to in the content.
